# Ribosome-mediated polymerization of long chain carbon and cyclic amino acids into peptides in vitro

**DOI:** 10.1038/s41467-020-18001-x

**Published:** 2020-08-27

**Authors:** Joongoo Lee, Kevin J. Schwarz, Do Soon Kim, Jeffrey S. Moore, Michael C. Jewett

**Affiliations:** 1 Department of Chemical and Biological Engineering and Center for Synthetic Biology, Northwestern University, Evanston, IL 60208 USA; 2 Department of Chemistry, University of Illinois at Urbana-Champaign, Urbana, IL 61801 USA; 3 The Beckman Institute for Advanced Science and Technology, University of Illinois at Urbana-Champaign, Urbana, IL 61801 USA

**Keywords:** Synthetic biology, Peptides, Protein design

## Abstract

Ribosome-mediated polymerization of backbone-extended monomers into polypeptides is challenging due to their poor compatibility with the translation apparatus, which evolved to use α-L-amino acids. Moreover, mechanisms to acylate (or charge) these monomers to transfer RNAs (tRNAs) to make aminoacyl-tRNA substrates is a bottleneck. Here, we rationally design non-canonical amino acid analogs with extended carbon chains (γ-, δ-, ε-, and ζ-) or cyclic structures (cyclobutane, cyclopentane, and cyclohexane) to improve tRNA charging. We then demonstrate site-specific incorporation of these non-canonical, backbone-extended monomers at the N- and C- terminus of peptides using wild-type and engineered ribosomes. This work expands the scope of ribosome-mediated polymerization, setting the stage for new medicines and materials.

## Abstract (web summary)

Backbone extended monomers are poorly compatible with the natural ribosomes, impeding their polymerization into polypeptides. Here the authors design non-canonical amino acid analogs with cyclic structures or extended carbon chains and used an engineered ribosome to improve tRNA-charging and incorporation into peptides.

## Introduction

**Fig. 1:**
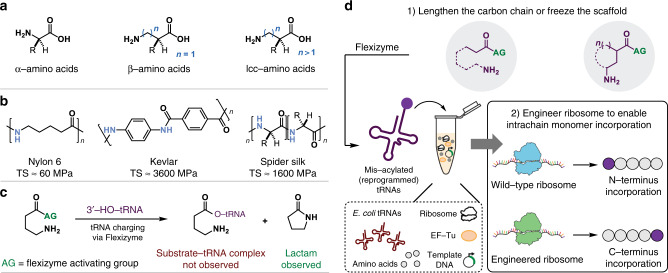
Expanding the chemical substrate scope of the translation apparatus to include long chain carbon and cyclic amino acids. **a** Substrates for translation compatible with the flexizyme (Fx) and cell-free protein synthesis (CFPS) platforms. Long chain carbon (lcc) amino acid incorporation into peptides has proved challenging. **b** Examples of prominent polyamide polymers that possess significantly different properties, such as tensile strength (TS), based on backbone length, monomer functionality, and/or monomer sequence. **c** tRNA charging of lcc amino acids by the Fx system has remained challenging due to the resulting intramolecular lactam formation. **d** Strategy for incorporation of long chain carbon amino acids via Fx and in vitro translation.

The cellular translation system (the ribosome and associated factors for protein biosynthesis) catalyzes the synthesis of sequence-defined polymers (polypeptides) using a set of amino-acylated transfer RNA (tRNA) substrates and a defined coding template (messenger RNA). In nature, only a limited set of α-l-amino acid monomers are utilized by this system, thereby limiting the potential diversity of polymers that can be synthesized. Over the past two decades, however, efforts to expand the genetic code have shown that the natural translation system is capable of selectively incorporating a wide range of non-canonical monomers^1–5^. These monomers include α-^6^, β-^7–9^, γ-^10–12^, D-^13,14^, aromatic^15–17^, aliphatic^15,18^, malonyl^16^, N-alkylated^19^, and oligomeric amino acid analogs^10,20,21^, among others (Fig. 1a).

Site-specific incorporation of such diverse chemistries into peptides and proteins has led to a wave of exciting applications. For example, foldamers incorporated into the N-terminus of a peptide have created macrocyclic foldamer–peptide hybrids with unique bioactivity^22^. In addition, benzoic acids and 1,3-dicarbonyl substrates have been incorporated into diverse aramid–peptide and polyketide–peptide hybrid molecules^15,16^, which may enable new classes of functional materials and polyketide natural products. Furthermore, β-amino acid peptides have made possible new protease resistant, peptidomimetic drugs^23–27^.

Having access to an even broader repertoire of monomers for ribosome-mediated polymerization holds promise to further increase the number of polymers that could be synthesized in a sequence-defined manner, which has been called the next “Holy Grail” of polymer science^28^. For example, polyamides (outside of polypeptides) make use of a key set of privileged molecular architectures to obtain exceptional polymer properties, such as improved thermal stability, elastic modulus, and tensile strength, based on polymer backbone and chain microstructure (i.e., Nylon-6 versus Kevlar^29,30^, Fig. 1b). The ability to introduce these architectures into polypeptides and modulate their properties could open new opportunities at the intersection of materials science and synthetic biology. However, direct incorporation of these monomers—such as long chain carbon amino acids (≥γ-)—has proved challenging for two key reasons. First, natural ribosomes have been evolutionarily optimized to polymerize α-l-amino acids, leading to poor compatibility with backbone-extended monomers. Second, acylating (or charging) these monomers to tRNAs to make aminoacyl-tRNA substrates is difficult. Chemical aminoacylation is technically difficult and laborious, aminoacyl-tRNA synthetases have not been evolved for these long chain carbon monomers, and efforts to use the flexizyme system (Fx, an aminoacyl tRNA synthetase-like ribozyme)^23,31^ have been unsuccessful, due to intramolecular lactam formation after the tRNA charging reaction (Fig. 1c)^10,12,25,32,33^. Taken together, these limitations have restricted the scope of long chain carbon (or backbone-extended) amino acid monomers incorporated into sequence-defined polyamides by the ribosome.

Here, we set out to address these limitations by investigating the Fx-catalyzed tRNA charging of γ-, δ-, ε-, and ζ-amino acids containing long  chain carbon structures and demonstrating subsequent in vitro incorporation of such amino acid derivatives into peptides by the ribosome. This stands distinct from our recent work to study flexizyme design rules associated with four chemically diverse scaffolds (phenylalanine, benzoic acid, heteroaromatic, and aliphatic monomers) with different electronic and steric factors^15^. Here, we consider how to avoid intramolecular nucleophilic attack of the monomer amino group of backbone-extended monomers to facilitate tRNA charging. In addition, we focus on long chain carbon and cyclic monomers, which is unique from many works showing the incorporation of a variety of non-canonical α-^6^ and β-amino acids^7,8,25,34^. We first confirm through NMR and LC–MS analysis that tRNA charging of linear γ-amino acids via flexizyme fails due to deleterious lactam formation (Fig. 1c and Supplementary Fig. 1). Next, we circumvent this limitation of Fx-catalyzed tRNA-charging by designing amino acid substrate architectures that control the intramolecular reaction kinetics of the tRNA:substrate complex by lengthening the carbon chain and/or introducing a rigid central architecture (Fig. 1d, top panel) such that lactam formation is reduced or avoided altogether. Then, we demonstrate incorporation of backbone-extended monomers into the N-terminus of peptides using wild-type ribosomes. Finally, we use a previously engineered ribosome^24,27,34^ with mutations in the peptidyl transferase center (PTC) to enable C-terminal incorporation of these non-canonical amino acids into a peptide (Fig. 1d, bottom panel).

## Results

### Long chain carbon and cyclic amino acid flexizyme charging

**Fig. 2:**
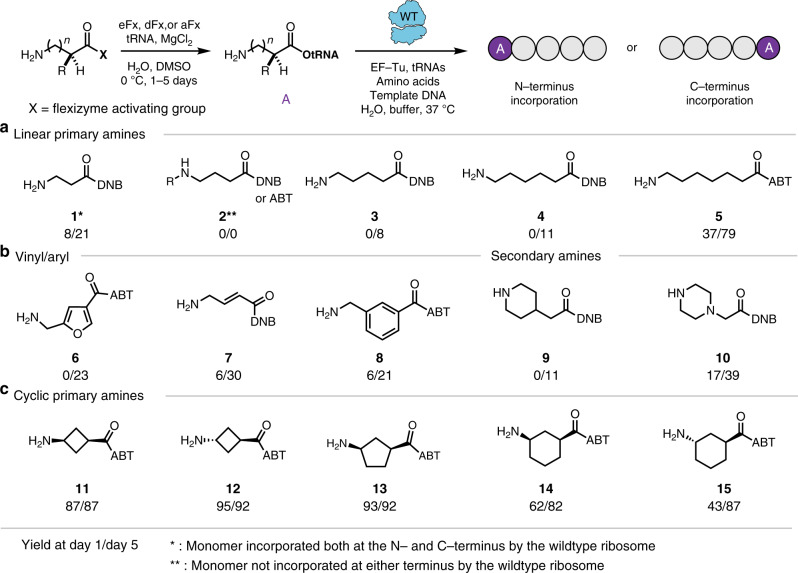
Systematic design of long chain carbon and cyclic amino acids. **a** The range of amino acids bearing a linear carbon chain was extended to γ-, δ-, ε-, and ζ-amino acids. Higher acylation yields by Fx were observed as the amino acid chain length increased, presumably because larger (>5-membered) ring formation via lactamization is kinetically less favorable than 5-membered ring formation. **b** Introducing cyclic and rigid bonds into substrates helps increase Fx acylation yields. **c** An increased acylation yield (from ~6% for **7** up to ~95% for **12**) was obtained for the γ-amino acids with a rigid bond (**7**) or cyclic structure (**11**–**15**). These data suggest the rigid carbon scaffold efficiently inhibits the intramolecular 5-membered lactam formation reaction. The acylation yield of each substrate represents the percent yield of a microhelix tRNA observed at 24 h/120 h (see Supplementary Information for gels). Data are representative of three independent experiments.

To gain insights about possible constraints for using Fx to charge long chain carbon amino acid substrates onto tRNAs, 10 substrates (**1**–**5** in Fig. 2a and **2i**–**2v** in the characterization section in Supplementary Information) were examined with increasing numbers of carbons in the monomer backbone. Dinitrobenzyl ester (DNB)-derivatized or amino-derivatized benzyl thioester (ABT)-activated forms of 3-aminopropanoic acid (**1**, β-alanine) and 4-aminobutyric acid (**2** and **2i**) were synthesized for Fx-mediated charging. We used a tRNA mimic, microhelix tRNA (mihx), to determine the yields of the Fx-mediated acylation reaction using the conventional Fx reaction condition^20^. Aminoacylation efficiency was estimated by acid-denaturing polyacrylamide gel electrophoresis (PAGE, Supplementary Fig. 1). We found that **1** was successfully charged, while **2** was not as previously reported^10,25^ (Fig. 2a and Supplementary Fig. 1). We tested four additional γ-amino acid substrates (4-methylaminobutyric acid (**2ii**) and 2,2-dimethylaminobutyric acid (**2iii**), *cis*- (**2iv**), and *trans*-2-aminocyclopropane-1-carboxylic acid (**2v**)) for Fx-mediated tRNA charging, but no γ-amino acid substrates (**2** and **2i**–**v**, see characterization section in Supplementary Information) were found to be charged (Supplementary Fig. 1), indicating our results are consistent with previous literature and that Fx-mediated charging of γ-amino acid analogs with a linear carbon chain is indeed challenging.

**Fig. 3:**
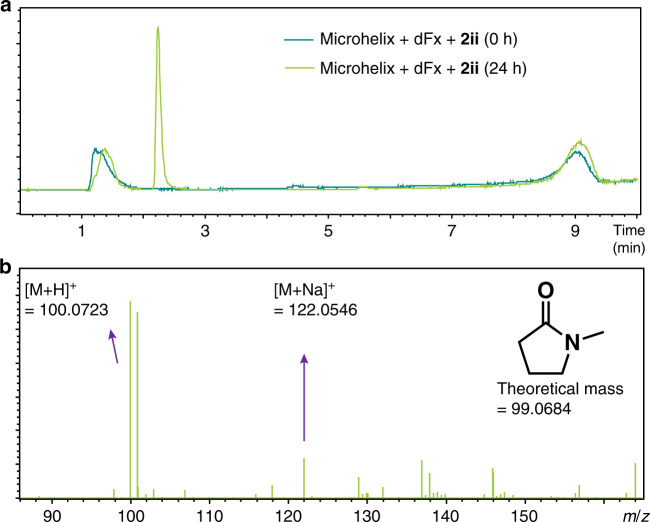
Observation of lactam in Fx-mediated acylation of γ-amino acid. The lactam produced (light green) in the Fx-mediated acylation of substrate **2ii** is observed. The extracted ion chromatogram **a** for the mixture of Fx reaction incubated for 24 h on ice showed a new peak corresponding to a theoretical mass of a lactam **b**. Data are representative of three independent experiments.

To confirm the hypothesis that lactam formation is the cause of poor tRNA charging results, we next investigated whether a lactam is observed in the Fx-catalyzed reaction. A Fx-catalyzed acylation reaction of 4-methylaminobutyric acid (**2ii**) with mihx was set up and monitored over 24 h. Notably, analysis by LC–MS of the reaction mixture incubated for 24 h yielded a single new peak (2.3 min, light green, Fig. 3a). The ESI-MS generated by combining mass spectra obtained across the peak at 2.3 min showed an accurate mass corresponding to the theoretical mass of the lactam, 1-methylpyrrolidin-2-one (Fig. 3b). Furthermore, a lactam is only observed when both Fx and mihx are present in the reaction mixture, suggesting that lactam formation is catalyzed by these species.

Next, we synthesized long chain carbon derivatives 5-aminopentanoic acid (**3**), 6-aminohexanoic acid (**4**), and 7-aminoheptanoic acid (**5**), to further support our hypothesis that acylation yields of tRNA would increase because the formation of larger rings (>5-membered) is less kinetically favorable than 5-membered ring formation. As expected, we observed higher acylation yields for increasing lengths of the carbon chain in the amino acid derivatives (Fig. 2a and Supplementary Fig. 1), further suggesting the deficiency of linear γ-amino acids in the genetic code reprogramming is due to the propensity for lactam formation amongst these substrates using Fx-mediated catalysis. Of note, this result is in a good agreement with a general rule for ring closure reactions^35,36^ that shows the rate constant for the 5-membered ring self-cyclization is the largest. The rate constant decreases by 1–2 orders of magnitude (i.e., self-cyclization slows) as the ring size increases from 5-members to 10-members^35^.

Based on these results, we sought to design molecular architectures that would circumvent intramolecular lactam formation by steric restriction of the amino and activated ester functionalities. We synthesized five substrates (**6**–**10** in Fig. 2b) containing a rigid spacer (cyclic, aryl, or vinyl) and tested acylation. Notably, all of the substrates (**6**–**10**), which are γ-amino acid and δ-amino acid, were successfully charged to tRNA using flexizymes. To further expand the range of the monomers for diverse polyamides, we synthesized five additional amino acids (**11**–**15** in Fig. 2c) containing a cyclic structure in the central region of amino acid. When these substrates were charged to tRNAs, we found the acylation yield was dramatically increased compared to the other γ-type amino acids, suggesting that the rigid cyclic carbon scaffold efficiently prevents the intramolecular 5-membered lactam formation reaction. This observation is consistent with our recently described design rules for flexizyme-catalyzed acylation^15^, as well as another recent report that showed incorporation of cyclic-gamma amino acids into peptides^12^. In short, the cyclic structures contain less steric hindrance about the carbonyl relative to structures (**1**–**5**) and increased electrophilicity relative to the conjugated structures (**6**–**8**) allowing for efficient tRNA attack^15^. Overall, we found that 13 non-canonical monomers were charged, with efficiencies of 6–95%, with (E)-4-aminobut-2-enoic acid (**7**) as the lowest and *trans*-3-aminocyclobutane-1-carboxylic acid (**12**) as the highest yield, respectively.

### Ribosomal polymerization of backbone-extended monomers

Next, we investigated whether the newly found flexizyme substrates charged to tRNAs are accepted by the natural protein translation machinery. The goal was to demonstrate that the ribosome was compatible with these substrates, rather than focus on a specific application. We performed the Fx-catalyzed acylation reaction for tRNAs under the same reaction conditions obtained from the acylation reaction of mihx (Supplementary Fig. 1). Previous works have shown that the acylation reaction yield and kinetics between in vitro-transcribed tRNA mimics (e.g., mihx or microhelix) and tRNAs are comparable^37–41^. After the Fx-mediated tRNA acylation, unreacted monomers were separated from the tRNAs using ethanol precipitation^20^ and the resulting tRNA fraction that includes the tRNA-substrates was supplemented as a mixture into a cell-free protein synthesis^42^ reaction containing a minimal set of components required for protein translation (PURExpress^TM^)^43^. We then determined incorporation of the non-canonical substrates into either the N- or C-terminus of a small model Streptavidin tag by MALDI mass spectrometry.

As the initiator tRNA, tRNA^fMet^ was selected for N-terminal incorporation studies. For C-terminal incorporation, we assessed several tRNAs (fMet, Pro1E2, GluE2, and AsnE2)^44^ previously engineered to efficiently incorporate non-canonical amino acids into polypeptides by the ribosome. We observed no significant difference in incorporation efficiency, depending on the codon variations. As such, Pro1E2^44^ was selected because it has an engineered D-arm and T-stem interacting with other protein translation factors such as EF-Tu and EF-P that can be additionally supplemented into the cell-free translation reaction when it is necessary to promote the incorporation of charged substrate^8,25,45^. For the codons, we used AUG (CAU anticodon), as it is the canonical start codon for N-terminal incorporation. For C-terminal incorporation, we selected the ACC codon (GGU anticodon), which decodes the Thr(ACC) codon on mRNA. This was selected because threonine is excluded from the polypeptide Streptavidin tag (WSHPQFEK) that was used for our study. This prevented corresponding endogenous tRNAs in the PURExpress^TM^ reaction from being aminoacylated and used in the translation reaction.

**Fig. 4:**
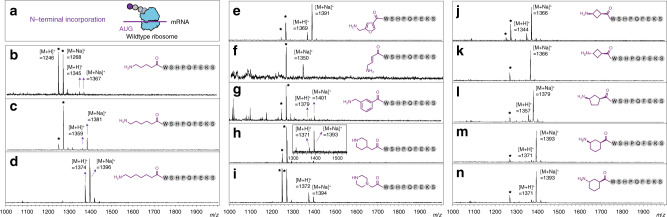
Ribosomal synthesis of N-terminal functionalized peptides with backbone-extended monomers. **a** All backbone-extended amino acids (**3**–**15**) charged to tRNA^fMet^(CAU) by Fx were incorporated into the N-terminus of a peptide by ribosome-mediated polymerization in the PURExpress^TM^ system. The peptides were purified via the Streptavidin tag (WSHPQFEK) and characterized by MALDI mass spectrometry. The observed mass of each peptide corresponds to the theoretical mass, which is **b** [M + H]^+^ = 1345; [M + Na]^+^ = 1367, **c** [M + H]^+^ = 1359; [M + Na]^+^ = 1381, **d** [M + H]^+^ = 1373; [M + Na]^+^ = 1395, **e** [M + H]^+^ = 1369; [M + Na]^+^ = 1391, **f** [M + Na]^+^ = 1351, **g** [M + H]^+^ = 1379; [M + Na]^+^ = 1401, **h** [M + H]^+^ = 1371; [M + Na]^+^ = 1393, **i** [M + H]^+^ = 1372; [M + Na]^+^ = 1394, **j** [M + H]^+^ = 1343; [M + Na]^+^ = 1365, **k** [M + Na]^+^ = 1365, **l** [M + H]^+^ = 1357; [M + Na]^+^ = 1379, **m** [M + H]^+^ = 1371; [M + Na]^+^ = 1393, **n** [M + H]^+^ = 1371; [M + Na]^+^ = 1393. The peaks denoted with an asterisk are a truncated peptide not bearing the target substrate at the N-terminus ([M + H]^+^ = 1246; [M + Na]^+^ = 1268). Data are representative of three independent experiments.

**Fig. 5:**
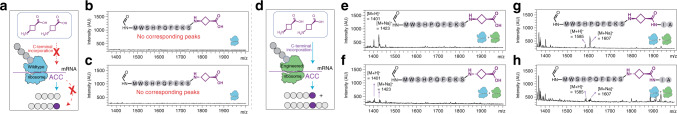
Ribosomal synthesis of peptides with aminocyclobutane-carboxylic acid (ACB). **a** Peptides were synthesized in the PURExpress^TM^ system using Fx-mediated tRNA^Pro1E2^(GGU), purified via the Streptavidin tag, and characterized by MALDI mass spectrometry. **b** and **c** *cis-*ACB and *trans*-ACB are not incorporated into the C-terminus of a peptide by the wild-type ribosome. **d** Engineered ribosomes facilitate C-terminal and mid-chain incorporation of *cis/trans*-ACB into peptides. **e** and **f** *cis-*ACB and *trans*-ACB. Peptides containing *cis/trans*-ACB at the C-terminus were observed when an engineered ribosome, developed by Maini et al.^24, 58^, was added into the protein translation reaction in vitro. **g** and **h** *cis* and *trans*-ACB. Additional amino acid residues (Ile and Ala) were elongated after the incorporation of *cis/trans*-ACB, demonstrating that the engineered ribosome enabled site-specific incorporation of ACB. Data are representative of three independent experiments. See Supplementary Fig. 2 for full spectrum.

We charged all 14 substrates onto tRNA^fMet^ (CAU) and tRNA^Pro1E2^ (GGU) to yield a set of acylated tRNAs, which were subsequently used in the PURExpress^TM^ translation reaction. The PURExpress^TM^ reaction was carried out in the presence of all *Escherichia coli* (>46) endogenous tRNAs, but only nine amino acids encoding the polypeptide Streptavidin tag (WSHPQFEK) and the non-canonical aminoacyl-tRNA substrate were used. Two different sets of amino acids (X + WSHPQFEK + T and M + WSHPQFEK + X) were used for the N- and C-terminus incorporation, respectively, where X indicates the position to which a Fx-charged backbone extended monomer is incorporated (Fig. 2a, see Supplementary Information in detail). Following translation (Fig. 4a), we found that every substrate that could be charged onto tRNAs was successfully incorporated into a peptide at the N-terminus, confirmed by a peak corresponding to a theoretical mass of peptide in MALDI spectra (Fig. 4b–n). However, attempts to produce a peptide containing these amino acids at the C-terminus were unsuccessful (Fig. 5a–c and Supplementary Fig. 2b, e). This is presumably because the C-terminal incorporation forming an amide bond with a nascent peptide requires more precise alignment of substrate in the PTC^46^ and the wild-type ribosome is not efficient at incorporation of non-canonical, backbone-extended substrates into polypeptides.

### Engineered ribosomes enhance incorporation of novel monomers

Recently, advances by the Hecht group showed that an engineered ribosome (termed 040329) enabled incorporation of dipeptides into a growing polymer chain by the ribosome^24,27^ in vivo and in vitro, where the ribosome forms an amide bond with the nascent peptide using the far-distance amine of a substrate. We hypothesized that this engineered ribosome would also be more permissive towards the backbone-extended monomers described here. To test this, we co-expressed the mutant ribosomes in cells using previously established protocols^47^ (see Supplementary Information for details). From these cells, we lysed and purified ribosomes through ultracentrifugation on a sucrose cushion (see Supplementary Information for details). The resulting ribosome sample contained a mixture of wild type and 040329 ribosomes, which were subsequently used in translation assays to determine their activity towards elongated backbone monomers. Based on previous literature, we expected the 040329 ribosomes to constitute around 25% of the purified ribosome population. To test the feasibility of incorporating long chain carbon amino acids into peptides with engineered ribosomes, we added the ribosome mixtures (Fig. 5d) into the PURExpress^TM^ system containing the substrates charged to tRNA^Pro1E2^(GGU) by Fx. In our MALDI mass spectrum, we observed a peak corresponding to the theoretical mass of the target peptide containing *cis*- and *trans*-3-aminocyclobutane-1-carboxylic acids (ACB, **11** and **12**, from Fig. 2, respectively) at the C-terminus (fMWSHPQFEKS**11/12** in Fig. 5e, f, and Supplementary Fig. 2c, f), which was not observed in the experiments with the wild-type ribosome alone (Fig. 5b, c, and Supplementary Fig. 2b, e). The relative percent yields of the target peptide containing *cis* and *trans*-ACB at the C-terminus were approximately 11% and 15%, respectively, based on the total of full-length and truncated peptide products (fMWSHPQFE, fMWSHPQFEK, and fMWSHPQFEKS, Supplementary Fig. 2).

We finally investigated whether additional amino acids can be elongated after the incorporation of *cis*-ACB and *trans*-ACB (**11** and **12**, Fig. 5g, h, and Supplementary Fig. 2d, g) at the C-terminus. We designed a new plasmid that encodes two additional amino acid residues, Ile (AUC) and Ala (GCC), and performed a PURExpress^TM^ reaction under the same reaction conditions, using a new set of 11 amino acids (M + WSHPQFEK + X + IA). While inefficient, we observed peaks corresponding to the theoretical mass of the target peptides (fMWSHPQFEKS**11/12**IA), demonstrating the engineered ribosome is capable of continuing to elongate following insertion of *cis*-ACB and *trans*-ACB.

## Discussion

In this work, we expanded the range of backbone-extended amino acid substrates for molecular translation. To do so, we investigated mechanistic aspects that limit the acylation step of γ-amino acids onto tRNAs by Fx. Then, through systematic and rational substrate design, we showed that a diverse repertoire of 15 amino acids with long chain carbon and cyclic structures could be acylated to tRNA by the Fx system in yields of 6–95%. Next, we demonstrated that these charged acylated tRNA-monomers could be used in ribosome-mediated polymerization, expanding the diversity of polyamides that can be produced by ribosomal synthesis.

While the field of genetic code expansion has incorporated hundreds of α-based non-canonical amino acids, until now, it was not known if the ribosome was capable of incorporating the backbone-extended (γ-, δ-, ε-, and ζ-) and cyclic (cyclobutane, cyclopentane, and cyclohexane) amino acid-based structures presented here. Our work shows that the ribosome is capable of polymerizing such structures using the genetic code reprogramming approach. Not surprisingly, the efficiency of incorporation, especially at the C-terminus or mid-chain, is low. This is likely because the shape, physiochemical, and dynamic properties of the ribosome have been evolved to work with canonical α-amino acids, or in the case of the modified ribosome 040329, β-amino acids^34^. It is likely that wild-type and 040329 ribosomes still discriminate against the backbone-extended stereoisomer monomers introduced here. Looking forward, the incorporation efficiency of such substrates could be improved by supplementing the combination of EF-P and engineered tRNAs^8,12,48^. In addition, in vitro ribosome assembly^49^ and selection^50^ platforms could evolve ribosomes with altered properties that increase incorporation efficiency of the backbone-extended monomers into peptides (i.e., form less truncated products) and facilitate the synthesis of polymers comprised solely of such monomers. Finally, extension to cellular systems with orthogonal engineered tethered, or stapled, ribosomes^51–55^ offers another exciting direction. However, the lack of aminoacyl tRNA-synthetases (aaRS) that charge the monomers into tRNA in the cell will need to be addressed.

By expanding the scope of long chain carbon and cyclic amino acids available for use in ribosome-mediated polymerization, we expect this work to motivate new directions in efforts to synthesize non-canonical sequence-defined polymers. For example, the monomers shown here could be directly used with in vitro screening and selection methods like mRNA or ribosome display to discover innovative peptide drugs^56^. In addition, future works could enable unique functional materials and polymers of defined atomic sequence, exact monodisperse length, and programmed stereochemistry.

## Methods

### General Fx-mediated acylation reaction

*Microhelix acylation*: 1 μL of 0.5 M HEPES (pH 7.5) or bicine (pH 8.8), 1 μL of 10 μM microhelix, and 3 μL of nuclease-free water were mixed in a PCR tube with 1 μL of 10 μM eFx, dFx, and aFx, respectively. The mixture was heated for 2 min at 95 °C and cooled down to room temperature over 5 min. 2 μL of 300 mM MgCl_2_ was added to the cooled mixture and incubated for 5 min at room temperature. Followed by the incubation of the reaction mixture on ice for 2 min, 2 μL of 25 mM activated ester substrate in DMSO was then added to the reaction mixture. The reaction mixture was further incubated for 16–120 h on ice in cold room.

*tRNA acylation*: 2 μL of 0.5 M HEPES (pH 7.5) or bicine (pH 8.8), 2 μL of 250 μM tRNA, 2 μL of 250 μM of a Fx selected on the microhelix experiment and 6 μL of nuclease-free water were mixed in a PCR tube. The mixture was heated for 2 min at 95 °C and cooled down to room temperature over 5 min. 4 μL of 300 mM MgCl_2_ was added to the cooled mixture and incubated for 5 min at room temperature. Followed by the incubation of the reaction mixture on ice for 2 min, 4 μL of 25 mM activated ester substrate in DMSO was then added to the reaction mixture. The reaction mixture was further incubated under the optimal reaction conditions determined by the microhelix experiment.

### In vitro synthesis of polyamides

*N-terminus incorporation*: As a reporter peptide, a T7 promoter-controlled DNA template (pJL1_StrepII) was designed to encode a streptavidin (Strep) tag and additional Ser and Thr codons (XWSHPQFEKST (Strep tag), where X indicates the position of the non-canonical amino acid substrate). The translation initiation codon AUG was used for N-terminal incorporation of the non-canonical amino acid substrate, X. Peptide synthesis was performed using only the 9 amino acids that decode the initiation codon AUG and the purification tag in the absence of the other 11 amino acids to prevent corresponding endogenous tRNAs from being aminoacylated and used in translation. The PURExpress^TM^ Δ (aa, tRNA) kit (NEB, E6840S) was used for polyamide synthesis reaction and the reaction mixtures were incubated at 37 °C for 3 h. The synthesized peptides were then purified using Strep-Tactin^®^-coated magnetic beads (IBA), denatured with SDS, and characterized by MALDI-TOF mass spectroscopy.

*C-terminus incorporation*: The same plasmid (pJL1-StrepII) encoding the same amino acids (MWSHPQFEKSX, where X indicates the position of the cyclic amino acid) was used for C-terminal incorporation and the cyclic amino acid was incorporated into the Thr codon (ACC) using a custom-made PURExpress^®^ Δ (aa, tRNA, ribosome) kit (NEB, E3315Z). For C-terminal incorporation, the wildtype ribosome provided in the kit was not used. 15 μM (final concentration) of the engineered ribosome was added to the reaction mixture only containing the 9 amino acids that decode the Strep tag and incubated at 37 °C for 3 h.

*Central-position incorporation*: A plasmid (pJL1-StrepII_TIA) designed to encode additional Ile and Ala downstream of Thr was used (see plasmid map for details) for incorporation of cyclic amino acid into the middle position of polyamide (MWSHPQFEKSXIA, where X indicates the position of the cyclic amino acid). The polyamide was produced using 11 amino acids in the PURExpress^TM^ Δ (aa, tRNA, ribosome) kit under the same reaction conditions used for C-terminal incorporation.

### Purification and characterization of polyamides

The polyamides containing a non-canonical amino acid were purified using an affinity tag purification technique and characterized by MALDI spectrometry as previously described^15^. For sample preparation, 1.5 μL of the purified peptide (0.1% SDS in water) was dried with 0.5 μL of the matrix (α-cyano-4-hydroxycinnamic acid in THF, 10 mg/mL). The dried sample was characterized on a Bruker rapifleX MALDI-TOF and processed using FlexControl v2.0 software (Bruker).

### Preparation of the cells containing 040329 ribosomes

A plasmid containing the rrnB operon under the pL promoter (pAM552) was used as the template for generating a modified rrnB gene with mutations 2057AGCGTGA2063 and 2502TGGCAG2507 in the 23S rDNA, referred to as the 040329 mutation. Plasmids harboring either the wild type (WT) or modified (040329) rrnB genes were transformed into POP2136 using electroporation and plated on LB-agar with 100 μg/mL of carbenicillin. The plates were incubated for 16–18 h at 30 °C (POP2136 harbors the cI repressor and thus represses expression of rRNA when grown at 30 °C). A single colony from the plate was used to inoculate 25 mL of LB-Miller containing 100 μg/mL of carbenicillin and the culture was grown for 16–18 h at 30 °C. When the culture had reached saturation, a 2 L culture of 2X YTP with 100 μg/mL of carbenicillin was pre-warmed to 42 °C, and inoculated with 20 mL of the overnight culture. Growth at 42 °C disrupts repression of the pL promoter and thus induces expression of the rrnB operon, which encodes for the 040329 mutant rRNA. Previous studies suggest the resulting ribosome population contains up to 20% of plasmid-encoded ribosomes. Optical density was measured regularly (every hour, then 15–30 min when close to the target OD) until the culture reached an OD between 0.4 and 0.6. Then, the cultures were pelleted via centrifugation at 8000 × *g* for 10 min. The resulting cell pellet was resuspended in Buffer A (see below for composition), and centrifuged again at 8000 × *g* for 10 min. Resuspension and centrifugation were repeated two more times for a total of three washes. After the final centrifugation, the cell pellet was flash frozen in liquid nitrogen and stored at −80 °C until further processing.

### Purification of ribosome mixtures

Frozen cell pellets were resuspended in Buffer A at a specified ratio (5 mL of Buffer A per 1 g of cell pellet) and lysed using homogenization at 20,000–25,000 psi. The resulting solution was centrifuged at 12,000 × *g* for 10 min to obtain clarified lysate. The clarified lysate was then layered onto a sucrose cushion at an even volumetric ratio (1 mL of cell lysate per 1 mL of Buffer B (see below for composition)) and ultracentrifuged at 90,000 × *g* for 18 h. This yielded a pellet on the bottom of the ultracentrifuge tube that contained ribosomes. The ribosome mixture was resuspended with Buffer C (see below for composition) with gentle shaking at 4 °C for 4–8 h, then diluted to obtain a concentration of 20–25 μM of ribosomes measured by absorbance at 260 nm on a spectrophotometer (1 A260 unit = 4.17 × 10^−5^ μM ribosomes). After complete resuspension and dilution, samples were aliquoted and flash frozen with liquid nitrogen, and stored at −80 °C until use in PURE reactions. Although further purification methods such as sucrose gradients could have been performed, the decision was made to use the crude mixture to maximize the absolute number of mutant ribosomes present in the ribosome mixture. *Reagents used—Buffer A*:* 20 mM Tris–HCl (pH 7.2), 100 mM NH_4_Cl, 10 mM MgCl_2_, 0.5 mM EDTA, 2 mM DTT; Buffer B: 20 mM Tris–HCl (pH 7.2), 500 mM NH_4_Cl, 10 mM MgCl_2_, 0.5 mM EDTA, 2 mM DTT, 37.7% (v/v) sucrose; Buffer C: 10 mM Tris–OAc, (pH 7.5), 500 mM NH_4_Cl, 7.5 mM Mg(OAc)_2_, 0.5 mM EDTA, 2 mM DTT. Oligos used for construction of 040329 ribosome plasmid: (1) To generate insert: 5′-AGTGTACCCGCGGCAAGACGAGCGTGACCCGTGAACCTTTACTATAGCTTGA-3′ and 5′-GCCCCAGGATGTGATGAGCCCTGCCAGAGGTGCCAAACACCGCCGTC-3′, 2) To generate backbone: 5′-GGCTCATCACATCCTGGGGCTG-3′ and 5′-CGTCTTGCCGCGGGTACACT-3′. Resulting PCR products were assembled together using isothermal DNA assembly^57^.

### Reporting summary

Further information on research design is available in the Nature Research Reporting Summary linked to this article.

## Supplementary information

Supplementary Information

Reporting Summary

## Data Availability

All characterization data (NMR and MS) generated or analyzed during this study are included in this article (and its Supplementary Information) or are available from the corresponding authors upon reasonable request. Source data are provided with this paper.

## Source data

Source Data
