# Supplementary material for: Ribosome-mediated polymerization of long chain carbon and cyclic amino acids into peptides in vitro
Source: Nat Commun. 2020 Aug 27;11:4304. doi: 10.1038/s41467-020-18001-x (PMC7452890; doi:10.1038/s41467-020-18001-x)
Supplement: Supplementary file 1 — Supplementary Information [file 41467_2020_18001_MOESM1_ESM.pdf]

Supplementary Information

Ribosome-mediated polymerization of long chain carbon and cyclic amino acids into peptides in vitro

Joongoo Lee[1,*], Kevin J. Schwarz[2,*], Do Soon Kim[1], Jeffrey S. Moore[2,3], Michael C. Jewett[1]

1. Department of Chemical and Biological Engineering, Northwestern University, Evanston, Illinois 60208
2. Department of Chemistry, University of Illinois at Urbana−Champaign, Urbana, Illinois 61801
3. The Beckman Institute for Advanced Science and Technology, University of Illinois at Urbana-Champaign, Urbana, IL 61801

*These authors contributed equally to this work.

To whom correspondence should be addressed. E-mail: jsmoore@illinois.edu or m-jewett@northwestern.edu

Table of Contents

Supplementary Methods.......... 4

Materials and Methods 4

General procedure A for formation of dinitrobenzyl esters & Boc deprotection.......... 4

General procedure B for formation of dinitrobenzyl esters & Boc deprotection.......... 4

General procedure C for formation of 4-((2-aminoethyl)carbamoyl)benzyl thioates & Boc deprotection.......... 5

Characterizations of Substrates 6

Preparation of DNA templates for RNAs 13

Preparation of Fx and tRNAs.......... 13

Supplementary Figures 14

Supplementary Figure 1. Acylation of microhelix with substrates 1-15 and 2i-2v. 14

Supplementary Figure 2. Characterization of the C-terminus functionalized peptide with *cis*- and *trans*-ACB (11-12). 15

Supplementary Figure 3. ^{1}H NMR (500 MHz, DMSO-d_6) of 1. 17

Supplementary Figure 4. ^{13}C NMR (125 MHz, DMSO-d_6) of 1. 17

Supplementary Figure 5. ^{1}H NMR (500 MHz, DMSO-d_6) of 2. 18

Supplementary Figure 6. ^{13}C NMR (125 MHz, DMSO-d_6) of 2. 18

Supplementary Figure 7. ^{1}H NMR (500 MHz, DMSO-d_6) of 2i. 19

Supplementary Figure 8. ^{13}C NMR (125 MHz, DMSO-d_6) of 2i. 19

Supplementary Figure 9. ^{1}H NMR (500 MHz, DMSO-d_6) of 2ii. 20

Supplementary Figure 10. ^{13}C NMR (125 MHz, DMSO-d_6) of 2ii. 20

Supplementary Figure 11. ^{1}H NMR (500 MHz, DMSO-d_6) of 2iii.......... 21

Supplementary Figure 12. ^{13}C NMR (125 MHz, DMSO-d_6) of 2iii. 21

Supplementary Figure 13. ^{1}H NMR (500 MHz, DMSO-d_6) of 2iv. 22

Supplementary Figure 14. ^{13}C NMR (125 MHz, DMSO-d_6) of 2iv. 22

Supplementary Figure 15. ^{1}H NMR (500 MHz, DMSO-d_6) of 2v. 23

Supplementary Figure 16. ^{13}C NMR (125 MHz, DMSO-d_6) of 2v. 23

Supplementary Figure 17. ^{1}H NMR (500 MHz, DMSO-d_6) of 3. 24

Supplementary Figure 18. ^{13}C NMR (125 MHz, DMSO-d_6) of 3. 24

Supplementary Figure 19. ^{1}H NMR (500 MHz, DMSO-d_6) of 4. 25

Supplementary Figure 20. ^{13}C NMR (125 MHz, DMSO-d_6) of 4. 25

Supplementary Figure 21. ^{1}H NMR (500 MHz, DMSO-d_6) of 5. 26

Supplementary Figure 22. ^{13}C NMR (125 MHz, DMSO-d_6) of 5. 26

Supplementary Figure 23. ^{1}H NMR (500 MHz, DMSO-d_6) of 6. 27
Supplementary Figure 24. ^{13}C NMR (125 MHz, DMSO-d_6) of 6. 27
Supplementary Figure 25. ^{1}H NMR (500 MHz, DMSO-d_6) of 7. 28
Supplementary Figure 26. ^{13}C NMR (125 MHz, DMSO-d_6) of 7. 28
Supplementary Figure 27. ^{1}H NMR (500 MHz, DMSO-d_6) of 8. 29
Supplementary Figure 28. ^{13}C NMR (125 MHz, DMSO-d_6) of 8. 29
Supplementary Figure 29. ^{1}H NMR (500 MHz, DMSO-d_6) of 9. 30
Supplementary Figure 30. ^{13}C NMR (125 MHz, DMSO-d_6) of 9. 30
Supplementary Figure 31. ^{1}H NMR (500 MHz, DMSO-d_6) of 10. 31
Supplementary Figure 32. ^{13}C NMR (125 MHz, DMSO-d_6) of 10. 31
Supplementary Figure 33. ^{1}H NMR (500 MHz, DMSO-d_6) of 11. 32
Supplementary Figure 34. ^{13}C NMR (125 MHz, DMSO-d_6) of 11. 32
Supplementary Figure 35. ^{1}H NMR (500 MHz, DMSO-d_6) of 12. 33
Supplementary Figure 36. ^{13}C NMR (125 MHz, DMSO-d_6) of 12. 33
Supplementary Figure 37. ^{1}H NMR (500 MHz, DMSO-d_6) of 13. 34
Supplementary Figure 38. ^{13}C NMR (125 MHz, DMSO-d_6) of 13. 34
Supplementary Figure 39. ^{1}H NMR (500 MHz, DMSO-d_6) of 14. 35
Supplementary Figure 40. ^{13}C NMR (125 MHz, DMSO-d_6) of 14. 35
Supplementary Figure 41. ^{1}H NMR (500 MHz, DMSO-d_6) of 15. 36
Supplementary Figure 42. ^{13}C NMR (125 MHz, DMSO-d_6) of 15. 36
Plasmid map 37
Supplementary References 38

Supplementary Methods

Materials and Methods

All reagents and solvents were commercial grade and purified prior to use when necessary. Dichloromethane was dried by passage through a column of activated alumina as described by Grubbs.[1]

Tert-butyl (2-(4-(mercaptomethyl)benzamido)ethyl) carbamate (ABT) was prepared according to the standard procedure.[2] All organic solutions were dried over $MgSO_4$. Thin layer chromatography (TLC) was performed using glass-backed silica gel (250 µm) plates. Flash chromatography was performed on a Biotage Isolera One automated purification system. UV light, and/or the use of $KMnO_4$ were used to visualize products.

Nuclear magnetic resonance spectra (NMR) were acquired on a Bruker Advance III-500 (500 MHz) or Varian Unity 500 (500 MHz) instrument and processed by ACD (v12.01) or Mnova (v14). Chemical shifts are measured relative to residual solvent peaks as an internal standard set to δ 7.26 and δ 77.0 ($CDCl_3$), and δ 2.50 and δ 39.5 (DMSO-d_6). Mass spectra were recorded on a Bruker AmaZon SL or Waters Q-TOF Ultima (ESI) and Impact-II or Waters 70-VSE (EI), spectrometers by use of the ionization method noted.

General procedure A for formation of dinitrobenzyl esters & Boc deprotection

To a glass vial with a stir bar was added carboxylic acid (1 equiv.), CH_2Cl_2 (1.0 M), triethylamine (1.5 equiv.), and 3,5-dinotrobenzyl chloride (1.2 equiv.). After stirring for 16 h at room temperature, the reaction mixture was diluted with EtOAc and washed with HCl (0.5 M aq.), $NaHCO_3$ (4 % (w/v) in water), brine, and dried over $MgSO_4$. The organic phase was concentrated to provide the crude product. The product was purified by flash column chromatography. The resulting fraction containing product was collected in a 100 mL flask and the solvent was removed under reduced pressure. 2 mL of HCl (4N in anhydrous dioxane) was added and let stir for 1h in room temperature. The resulting product was transferred to a 20 mL glass vial and dried under high vacuum overnight to give final product.

General procedure B for formation of dinitrobenzyl esters & Boc deprotection

To a flame-dried vial with septa and stir bar was added carboxylic acid (1.0 equiv.), 1-ethyl-3-(3-dimethylaminopropyl) carbodiimide hydrochloride (EDCI) (2.0 equiv.), dimethylamino pyridine

(2.0 equiv.), evacuated and flushed with $N_{2(g)}$ three times, then anhydrous CH_2Cl_2 (0.1 M) was added via syringe. The reaction was then let stir for 10 minutes before dinitrobenzyl alcohol (0.1M in anhydrous CH_2Cl_2) was added dropwise via syringe over 60 seconds. The reaction was then stirred at 22 °C for 16h. The reaction was diluted with DCM, added to a separatory funnel, rinsed with HCl (1.0 M aq.), H_2O, $NaHCO_3$ (3.0 M aq.), dried with $NaSO_4$, filtered, then silica (SiO_2) was added and condensed under reduced pressure. The compound/silica mixture was then dry loaded and purified by silica gel column chromatography [solvent system: hexanes-ethyl acetate; 9:1 – 2:8].

General procedure C for formation of 4-((2-aminoethyl)carbamoyl)benzyl thioates & Boc deprotection

To a flame-dried vial with septa and stir bar was added carboxylic acid (1.0 equiv.), 1-ethyl-3-(3-dimethylaminopropyl) carbodiimide hydrochloride (EDCI) (2.0 equiv.), dimethylamino pyridine (2.0 equiv.), evacuated and flushed with $N_{2(g)}$ three times, then anhydrous CH_2Cl_2 (0.1 M) was added via syringe. The reaction was then let stir for 10 minutes before *Tert*-butyl (2-(4-(mercaptomethyl)benzamido)ethyl) carbamate (0.1M in anhydrous CH_2Cl_2) was added dropwise via syringe over 60 seconds. The reaction was then stirred at 22 °C for 16h. The reaction was diluted with DCM, added to a separatory funnel, rinsed with HCl (1.0 M aq.), H_2O, $NaHCO_3$ (3.0 M aq.), dried with $NaSO_4$, filtered, then silica (SiO_2) was added and condensed under reduced pressure. The compound/Silica mixture was then dry loaded and purified by silica gel column chromatography [solvent system: hexanes-ethyl acetate; 8:3 – 1:9].

The resulting oil or solid was placed in a 20 mL scintillation vial with stir bar and 2 mL of HCl (4N in anhydrous dioxane) was added and let stir for 4h. The solution condensed under reduced pressure, then 5 mL of diethyl ether was added and the heterogeneous mixture was sonicated for 5 minutes. The mixture was filtered, and the filter cake rinsed with diethyl ether. The solid was collected and dried under vacuum to give final product.

Characterizations of Substrates

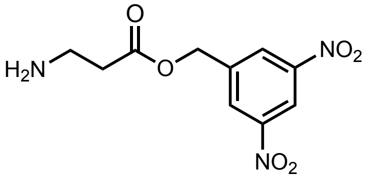

3,5-dinitrobenzyl 3-aminopropanoate (1). Prepared according to general procedure A using N-Boc-beta-alaine (62.4 mg, 0.33 mmol), triethylamine (70 µL, 0.50 mmol), 3,5-dinitrobenzyl chloride (86 mg, 0.40 mmol) and dichloromethane (0.5 mL). The product was obtained as a white powder (45 mg, 51 %). ^{1}H NMR (500 MHz, DMSO-*d*$_6$) δ 8.81 (t, *J* = 2.1 Hz, 1H), 8.70 (s, *J* = 2.1 Hz, 2H), 5.39 (s, 2H), 3.07 (t, *J* = 6.7 Hz, 2H), 2.80 (t, *J* = 7.2 Hz, 2H). ^{13}C NMR (125 MHz, DMSO-*d*$_6$) ppm 172.3, 148.6, 148.5, 142.3, 129.7 (2C), 118.8, 61.6, 35.2, 31.9; HRMS (m/z): [M]$^+$ calcd. for $C_{10}H_{11}N_3O_6$ 270.2107, found 270.2238.

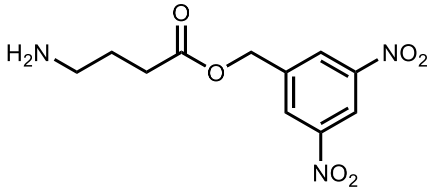

3,5-dinitrobenzyl-amino-4-butanoate (2). Prepared according to general procedure A using N-Boc-4-aminobutanoic acid (71.6 mg, 0.33 mmol), triethylamine (70 µL, 0.50 mmol), 3,5-dinitrobenzyl chloride (86 mg, 0.40 mmol) and dichloromethane (0.5 mL). The product was obtained as a white powder (65 mg, 70 %). ^{1}H NMR (500 MHz, 500 MHz, DMSO-*d*$_6$) δ 8.80 (t, *J* = 2.3 Hz, 1H), 8.59 (d, *J* = 2.1 Hz, 2H), 7.98 (s, 3H), 5.37 (s, 2H), 2.86-2.79 (m, 2H), 2.58 (t, *J* = 7.5 Hz, 2H), 1.85 (q, *J* = 7.6, 7.7, 2H); ^{13}C NMR (125 MHz, DMSO-*d*$_6$) ppm 172.4, 148.5 (2C), 141.0, 128.7 (2C), 118.6, 64.2, 38.4, 30.6, 22.7; HRMS (m/z): [M]$^+$ calcd. For $C_{11}H_{13}N_3O_6$ 204.24, found 204.12.

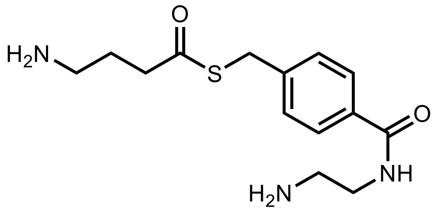

S-(4-((2-aminoethyl)carbamoyl)benzyl) 4-aminobutanethioate (2i). Prepared according to general procedure C using 7-((tert-butoxycarbonyl)amino) butanoic acid (50.8 mg, 0.25 mmol), 1-ethyl-3-(3-dimethylaminopropyl) carbodiimide hydrochloride (EDCI) (95.9 mg, 0.50 mmol), dimethylamino pyridine (61.1 mg, 0.50 mmol), t*ert*-butyl (2-(4-(mercaptomethyl)benzamido)ethyl) carbamate (84.6 mg, 0.25 mmol). The product was obtained as a white powder (40.7 mg, 55%). Silica gel column chromatography [Solvent System: Hexanes-Ethyl Acetate; 1:1, Rf= 0.1]. ^{1}H NMR (500 MHz, DMSO-*d*$_6$) δ 8.76 (s, 1H), 8.15 (s, 3H), 8.06 (s, 3H), 7.79 (d, *J* = 6.8 Hz, 2H), 7.29 (d, *J* = 7.1 Hz, 2H), 4.09 (s, 2H), 3.43 (s, 3H), 2.88 (s, 2H), 2.42 (s, 1H), 1.78 (s, 2H). ^{13}C NMR (126 MHz, DMSO-*d*$_6$) δ 197.20, 166.13, 141.15, 132.63, 128.39, 127.57, 39.87, 38.41, 37.77, 36.96, 31.80, 22.63. HRMS (m/z): [M]$^+$ calcd. for $C_{14}H_{22}N_3O_2S$ 297.1511, found 297.1511.

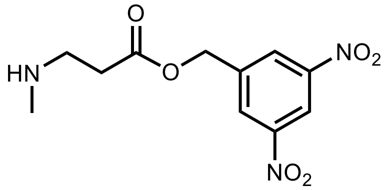

3,5-dinitrobenzyl 4-(methylamino)butanoate (2ii). Prepared according to general procedure A using 4-((boc-(methyl)amino)butanoic acid (67 mg, 0.33 mmol), triethylamine (70 µL, 0.50 mmol), 3,5-dinitrobenzyl chloride (86 mg, 0.40 mmol) and dichloromethane (0.5 mL). The product was obtained as a yellow powder (70 mg, 72%). ^{1}H NMR (500 MHz, DMSO-d_6) δ 8.86 (s, 2H), 8.72 (s, 1H), 8.59 (s, 2H), 4.76 (s, 2H), δ 2.86 (dq, *J* = 12.4, 6.9 Hz, 2H), 2.34 (t, *J* = 7.3 Hz, 2H), 1.81 (p, *J* = 7.5 Hz, 2H). ^{13}C NMR (125 MHz, DMSO-d_6) ppm 173.9, 148.4, 147.9, 128.6, 126.7 (2C), 117.4, 61.5, 47.9, 32.7 30.9, 21.3; HRMS (m/z): $[M]^+$ calcd. for $C_{12}H_{15}N_3O_6$ 298.10, found 298.14.

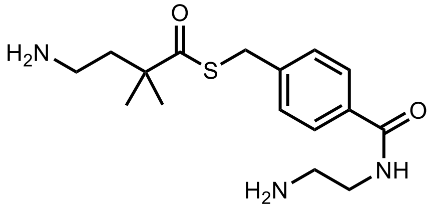

S-(4-((2-aminoethyl)carbamoyl)benzyl) 4-amino-2,2-dimethylbutanethioate (2iii). Prepared according to general procedure C using 4-((tert-butoxycarbonyl)amino)-2,2-dimethylbutanoic acid (57.8 mg, 0.25 mmol), 1-ethyl-3-(3-dimethylaminopropyl) carbodiimide hydrochloride (EDCI) (95.9 mg, 0.50 mmol), dimethylamino pyridine (61.1 mg, 0.50 mmol), t*ert*-butyl (2-(4-(mercaptomethyl)benzamido)ethyl) carbamate (84.6 mg, 0.25 mmol). The product was obtained as a white powder (51.7 mg, 64%). Silica gel column chromatography [Solvent System: Hexanes-Ethyl Acetate; 1:1, Rf= 0.1]. ^{1}H NMR (500 MHz, DMSO-d_6) δ 8.77 (s, 1H), 8.13 (s, 4H), 8.03 (s, 3H), 7.82 (d, *J* = 7.2 Hz, 2H), 7.33 (d, *J* = 7.4 Hz, 2H), 4.11 (s, 2H), 3.47 (s, 3H), 2.92 (s, 2H), 2.61 (s, 2H), 1.90 – 1.70 (m, 2H), 1.14 (s, 6H). ^{13}C NMR (126 MHz, DMSO-d_6) δ 204.04, 166.23, 141.10, 132.71, 128.42, 127.62, 47.78, 38.47, 36.99, 34.93, 31.71, 24.53. HRMS (m/z): $[M]^+$ calcd. for $C_{16}H_{25}N_3O_2S$ 325.1824, found 325.1825.

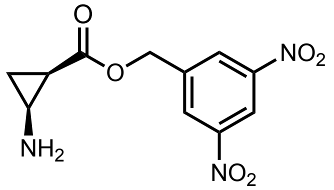

rac-*cis*-3,5-dinitrobenzyl-2-aminocyclopropane-1-carboxylate (2iv). Prepared according to general procedure A using *cis*-2-Boc-aminocyclopropane-1-carboxylic acid (66.4 mg, 0.33 mmol), triethylamine (70 µL, 0.50 mmol), 3,5-dinitrobenzyl chloride (86 mg, 0.40 mmol) and dichloromethane (0.5 mL). The product was obtained as a white powder (48.2 mg, 52 %). ^{1}H NMR (500 MHz, DMSO-d_6) δ 8.82 (t, *J* = 2.0 Hz, 1H), 8.73 (d, *J* = 0.9 Hz, 2H), 5.42 (dd, *J* = 44.2, 13.0 Hz, 2H), 2.34-2.26 (m, 2H), 2.22-2.09 (m, 2H). ^{13}C NMR (126 MHz, DMSO-d_6) δ 171.53, 148.54 (2C), 140.65, 129.08 (2C), 118.70, 64.70, 45.95, 25.44, 20.29. HRMS (m/z): $[M]^+$ calcd. for $C_{11}H_{11}N_3O_6$ 282.0726, found 282.0733.

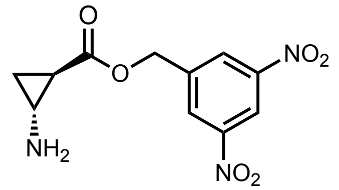

rac-*trans*-3,5-dinitrobenzyl-2-aminocyclopropane-1-carboxylate (2v). Prepared according to general procedure A using *trans*-2-Boc-aminocyclopropane-1-carboxylic acid (66.4 mg, 0.33 mmol), triethylamine (70 µL, 0.50 mmol), 3,5-dinitrobenzyl chloride (86 mg, 0.40 mmol) and dichloromethane (0.5 mL). The product was obtained as a white powder (35.3 mg, 38 %). ^{1}H NMR (500 MHz, DMSO-d_6) δ 8.79 (s, 1H), 8.767 (broad, 2H), 5.36 (broad, 2H), 3.66 (t, J = 22.6 Hz, 1H), 2.74 (t, J = 47.9 Hz, 1H), 1.6-1.2 (m, 2H). ^{13}C NMR (126 MHz, DMSO-d_6) δ 172.44, 148.53 (2C), 141.09, 128.61 (2C), 118.57, 64.14, 44.10, 29.51, 26.62. HRMS (m/z): [M]$^+$ calcd. for $C_{11}H_{11}N_3O_6$ 282.0726, found 282.0729.

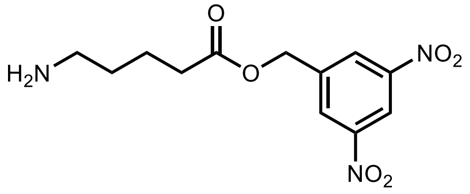

3,5-dinitrobenzyl 5-aminopentanoate (3). Prepared according to general procedure A using Boc-5-Ava-OH (72 mg, 0.33 mmol), triethylamine (70 µL, 0.50 mmol), 3,5-dinitrobenzyl chloride (86 mg, 0.40 mmol) and dichloromethane (0.5 mL). The product was obtained as a yellow oil (51 mg, 53 %). ^{1}H NMR (500 MHz, DMSO-d_6) δ 8.80 (t, J = 2.1 Hz, 1H), 8.67 (d, J = 2.0 Hz, 2H), 7.89 (s, 3H), 5.36 (s, 2H), 2.82-2.77 (m, 2H), 2.49 (t, J = 7.2 Hz, 2H), 1.66-1.54 (m, 4H); ^{13}C NM R (125 MHz, DMSO-d_6) ppm 172.8, 148.5 (2C), 141.0, 128.6 (2C), 118.5, 64.0, 38.8, 33.0, 26.8, 21.7; HRMS (m/z): [M]$^+$ calcd. for $C_{12}H_{16}N_3O_6$ 298.27, found 298.11

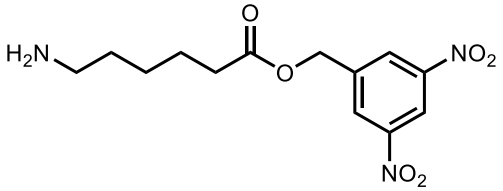

3,5-dinitrobenzyl 6-aminohexanoate (4). Prepared according to general procedure A using Boc-5-Ahx-OH (76 mg, 0.33 mmol), triethylamine (70 µL, 0.50 mmol), 3,5-dinitrobenzyl chloride (86 mg, 0.40 mmol) and dichloromethane (0.5 mL). The product was obtained as a white solid (64 mg, 62 %). ^{1}H NMR (500 MHz, $CDCl_3$) δ 8.80 (t, J = 2.1 Hz, 1H), 8.66 (d, J = 2.0 Hz, 2H), 7.87 (s, 3H), 5.36 (s, 2H), 2.78-2.72 (m, 2H), 2.45 (t, J = 7.6 Hz, 2H), 1.62-1.53 (m, 4H), 1.38-1.31 (m, 2H); ^{13}C NMR (125 MHz, DMSO-d_6) ppm 173.0, 148.5 (2C), 141.9 , 128.5 (2C), 118.5, 63.9, 38.9, 33.5, 27.0, 25.7, 24.2; HRMS (m/z): [M]$^+$ calcd. for $C_{13}H_{17}N_3O_6$ 312.29, found 312.13.

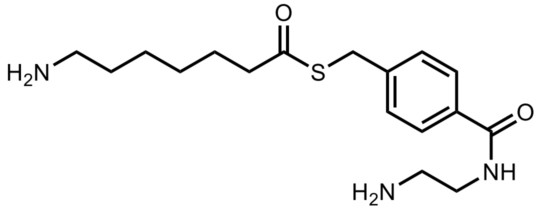

S-(4-((2-aminoethyl)carbamoyl)benzyl) 7-aminoheptanethioate (5). Prepared according to general procedure C using 7-((tert-butoxycarbonyl)amino) heptanoic acid (105.5 mg, 0.43 mmol), 1-ethyl-3-(3-dimethylaminopropyl) carbodiimide hydrochloride (EDCI) (165.1 mg, 0.86 mmol), dimethylamino pyridine (105.2 mg, 0.86 mmol), t*ert*-butyl (2-(4-(mercaptomethyl)benzamido)ethyl) carbamate (145 mg, 0.43 mmol). The product was obtained as a white powder (133.7 mg, 92%). Silica gel column chromatography [Solvent System: Hexanes-Ethyl Acetate; 1:1, Rf= 0.1]. ^{1}H NMR (500 MHz, DMSO-d_6) δ 8.85 (t, *J* = 5.5 Hz, 1H), 8.22 (s, 3H), 8.04 (s, 3H), 7.89 (d, *J* = 8.2 Hz, 2H), 7.37 (d, *J* = 8.1 Hz, 2H), 4.16 (s, 2H), 2.98 (q, *J* = 5.5 Hz, 2H), 2.72 (q, *J* = 6.6 Hz, 2H), 2.61 (t, *J* = 7.3 Hz, 2H), 2.51 (t, *J* = 1.9 Hz, 1H), 1.55 (dp, *J* = 15.9, 7.9, 7.5, 7.3 Hz, 4H), 1.38 – 1.21 (m, 4H). ^{13}C NMR (126 MHz, DMSO-d_6) δ 198.11, 166.32, 141.50, 132.74, 128.48, 127.72, 42.95, 38.62, 38.54, 37.10, 31.82, 27.64, 26.67, 25.44, 24.81. HRMS (m/z): $[M]^+$ calcd. for $C_{17}H_{27}N_3O_2S$ 339.1980, found 339.1982.

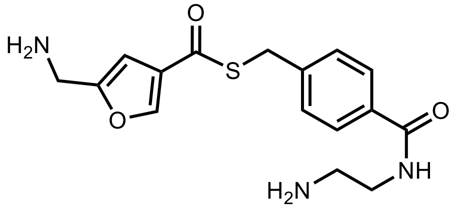

S-(4-((2-aminoethyl)carbamoyl)benzyl) 5-(aminomethyl)furan-3-carbothioate (6). Prepared according to general procedure C using 5-(((tert-butoxycarbonyl)amino)methyl)furan-3-carboxylic acid (60.3 mg, 0.25 mmol), 1-ethyl-3-(3-dimethylaminopropyl) carbodiimide hydrochloride (EDCI) (95.9 mg, 0.50 mmol), dimethylamino pyridine (61.1 mg, 0.50 mmol), t*ert*-butyl (2-(4-(mercaptomethyl)benzamido)ethyl) carbamate (84.6 mg, 0.25 mmol). The product was obtained as a yellow powder (68.5 mg, 82%). Silica gel column chromatography [Solvent System: Hexanes-Ethyl Acetate; 1:1, Rf= 0.1]. ^{1}H NMR (500 MHz, DMSO-d_6) δ 8.78 (s, 1H), 8.60 (m, 4H), 8.14 (s, 3H), 7.84 (d, *J* = 7.6 Hz, 2H), 7.39 (d, *J* = 7.5 Hz, 2H), 6.88 (s, 1H), 4.29 (s, 2H), 4.05 (s, 2H), 3.47 (d, *J* = 5.6 Hz, 2H), 2.93 (s, 2H). ^{13}C NMR (126 MHz, DMSO-d_6) δ 183.42, 166.23, 150.18, 147.34, 141.06, 132.82, 128.57, 127.67, 126.52, 108.03, 38.48, 37.03, 34.70, 31.45. HRMS (m/z): $[M]^+$ calcd. for $C_{16}H_{21}N_3O_3S$ 335.1304, found 335.1304.

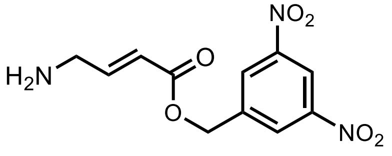

3,5-dinitrobenzyl (E/Z)-4-aminobut-2-enoate (7). Prepared according to general procedure A using (E)-4-((tert-butoxycarbonyl)amino)but-2-enoic acid (66.4 mg, 0.33 mmol), triethylamine (70 µL, 0.50 mmol), 3,5-dinitrobenzyl chloride (86

mg, 0.40 mmol) and dichloromethane (0.5 mL). The product was obtained as a yellow powder (24.1 mg, 26%). Silica gel column chromatography [Solvent System: Hexanes-Ethyl Acetate; 1:1, Rf= 0.1]. ^{1}H NMR (500 MHz, DMSO-d_6) δ 8.81 (t, J = 2.2 Hz, 1H), 8.69 (d, J = 2.0 Hz, 2H), 8.39 (s, 3H), 6.97 (m, 1H), 6.34 – 6.19 (m, 1H), 5.47 (s, 2H), 3.71 (d, J = 5.4 Hz, 2H). ^{13}C NMR (126 MHz, DMSO-d_6) δ 164.53, 148.08, 141.86, 140.32, 130.69, 128.28, 122.76, 118.25, 63.95. HRMS (m/z): $[M]^+$ calcd. for $C_{11}H_{12}N_3O_6$ 282.0726, found 282.0728.

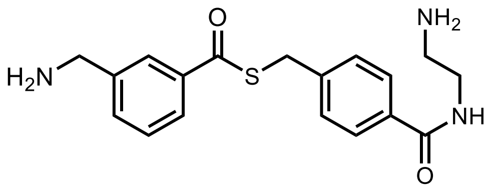

S-(4-((2-aminoethyl)carbamoyl)benzyl) 3-(aminomethyl)benzothioate (8). Prepared according to general procedure C using 3-(((tert-butoxycarbonyl)amino)methyl)benzoic acid (108.1 mg, 0.43 mmol), 1-ethyl-3-(3-dimethylaminopropyl) carbodiimide hydrochloride (EDCI) (165.1 mg, 0.86 mmol), dimethylamino pyridine (105.2 mg, 0.86 mmol), t*ert*-butyl (2-(4-(mercaptomethyl)benzamido)ethyl) carbamate (145 mg, 0.43 mmol). The product was obtained as a white powder (98.9 mg, 67%). Silica gel column chromatography [Solvent System: Hexanes-Ethyl Acetate; 1:1, Rf= 0.1]. ^{1}H NMR (500 MHz, DMSO-d_6) δ 8.79 (t, J = 5.5 Hz, 1H), 8.52 (s, 3H), 8.17 (s, 3H), 7.99 (s, 1H), 7.83 (d, J = 8.2 Hz, 3H), 7.75 (d, J = 8.0 Hz, 1H), 7.50 (t, J = 7.8 Hz, 1H), 7.39 (d, J = 8.1 Hz, 2H), 4.31 (s, 2H), 4.02 (q, J = 5.8 Hz, 2H), 3.44 (q, J = 6.0 Hz, 2H), 2.89 (q, J = 5.9 Hz, 2H). ^{13}C NMR (126 MHz, DMSO-d_6) δ 190.32, 166.31, 141.10, 136.24, 135.28, 134.78, 132.92, 129.43, 128.71, 127.78, 127.62, 126.90, 41.66, 38.54, 37.12, 32.16. HRMS (m/z): $[M]^+$ calcd. for $C_{18}H_{23}N_3O_2S$ 345.1511, found 345.1511.

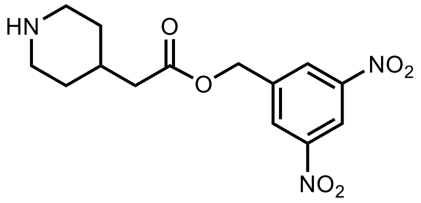

3,5-dinitrobenzyl 2-(piperidin-4-yl)acetate (9). Prepared according to general procedure A using N-Boc-4-piperidineacetic acid (80 mg, 0.33 mmol), triethylamine (70 µL, 0.50 mmol), dinitrobenzyl chloride (86 mg, 0.40 mmol) and dichloromethane (0.3 mL). The product was obtained as a yellow oil (66 mg, 62%). ^{1}H NMR (500 MHz, DMSO-d_6) δ; 8.72 (t, J = 2.0 Hz, 1H), 8.59 (d, J = 1.7 Hz, 2H), 3.15 (d, J = 12.4 Hz, 2H), 2.79 (td, J = 12.7, 2.8 Hz, 2H), 2.37 (d, 2H), 1.99-1.90 (m, 1H), 1.74 (d, J = 14.0 Hz, 2H), 1.33 (qd, J = 12.8, 4.1 Hz, 2H); ^{13}C NMR (125 MHz, DMSO-d_6) ppm 171.7, 148.5 (2C), 141.0, 128.5 (2C), 118.5, 64.0, 43.2 (2C), 30.6, 28.4 (2C); HRMS (m/z): $[M]^+$ calcd. for $C_{14}H_{17}N_3O_6$ 324.31, found 324.09.

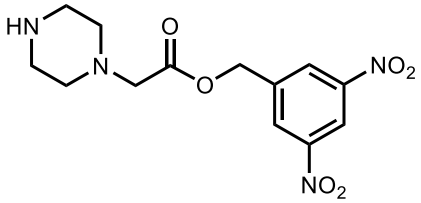

3,5-dinitrobenzyl 2-(piperazin-1-yl)acetate (10). Prepared according to general procedure A using 2-(4-Boc-1-piperazinyl)acetic acid (80 mg, 0.33 mmol), triethylamine (70 μL, 0.50 mmol), 3,5-dinitrobenzyl chloride (86 mg, 0.40 mmol) and dichloromethane (0.3 mL). The product was obtained as a white powder (87 mg, 82%). ^{1}H NMR (500 MHz, DMSO-d_6) δ; 2.69 (t, *J* = 4.9 Hz, 4H), 2.98 (t, *J* = 5.1 Hz, 4H), 3.41 (s, 2H), 5.31 (s, 2H), 8.61 (d, *J* = 1.1 Hz, 2H), 8.73 (t, *J* = 2.1, 1H); ^{13}C NMR (125 MHz, DMSO-d_6) 170.0, 148.5 (2C), 140.9, 128.8 (2C), 118.8, 64.0, 57.9, 49.1 (2C), 43.3 (2C); HRMS (m/z): $[M]^+$ calcd. for $C_{13}H_{16}N_4O_6$ 325.11, found 325.22.

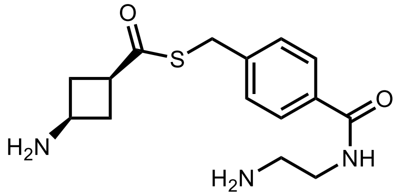

S-(4-((2-aminoethyl)carbamoyl)benzyl) (1s,3s)-3-aminocyclobutane-1-carbothioate (11). Prepared according to general procedure C using (1s,3s)-3-((tert-butoxycarbonyl)amino)cyclobutane-1-carboxylic acid (92.5 mg, 0.43 mmol), 1-ethyl-3-(3-dimethylaminopropyl) carbodiimide hydrochloride (EDCI) (165.1 mg, 0.86 mmol), dimethylamino pyridine (105.2 mg, 0.86 mmol), t*ert*-butyl (2-(4-(mercaptomethyl)benzamido)ethyl) carbamate (145 mg, 0.43 mmol). The product was obtained as a white powder (103.3 mg, 78%). Silica gel column chromatography [Solvent System: ^{1}H NMR (500 MHz, Methanol-d_4) δ 7.81 (d, *J* = 7.3 Hz, 2H), 7.40 (d, *J* = 7.3 Hz, 2H), 4.19 (s, 2H), 3.74 (d, *J* = 10.5 Hz, 1H), 3.65 (s, 2H), 3.29 – 3.22 (m, 1H), 3.16 (s, 2H), 2.59 (s, 2H), 2.38 (s, 2H). ^{13}C NMR (126 MHz, Methanol-d_4) δ 199.70, 170.55, 143.56, 133.69, 130.04, 128.84, 42.36, 41.06, 40.33, 38.77, 33.30, 32.37. HRMS (m/z): $[M]^+$ calcd. for $C_{15}H_{21}N_3O_2S$ 309.1511, found 309.1512.

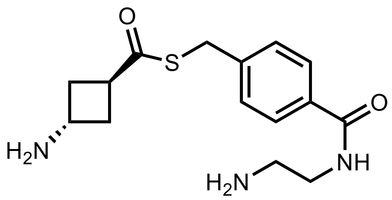

S-(4-((2-aminoethyl)carbamoyl)benzyl) (1r,3r)-3-aminocyclobutane-1-carbothioate (12). Prepared according to general procedure C using (1r,3r)-3-((tert-butoxycarbonyl)amino)cyclobutane-1-carboxylic acid (92.9 mg, 0.43 mmol), 1-ethyl-3-(3-dimethylaminopropyl) carbodiimide hydrochloride (EDCI) (165.6 mg, 0.86 mmol), dimethylamino pyridine (105.4 mg, 0.86 mmol), t*ert*-butyl (2-(4-(mercaptomethyl)benzamido)ethyl) carbamate (145 mg, 0.43 mmol). The product was obtained as a white powder (100.7 mg, 76%). Silica gel column chromatography [Solvent System: Hexanes-Ethyl Acetate; 1:1, Rf= 0.1]. ^{1}H NMR (500 MHz, Methanol-d_4) δ 8.72 (s, 1H), 7.82 (d, *J* = 8.0 Hz, 2H), 7.43 (d, *J* = 8.0 Hz, 2H), 4.23 (s, 2H), 3.90 (t, *J* = 7.7 Hz, 1H), 3.65 (q, *J* = 5.7 Hz,

2H), 3.50 (dp, J = 10.0, 5.2, 4.2 Hz, 1H), 3.16 (t, J = 5.9 Hz, 2H), 2.69 – 2.56 (m, 2H), 2.45 (q, J = 9.7 Hz, 2H). ^{13}C NMR (126 MHz, DMSO-d_6) δ 199.63, 166.26, 141.13, 132.76, 128.49, 127.66, 42.81, 40.90, 38.50, 37.04, 31.92, 29.97. HRMS (m/z): $[M]^+$ calcd. for $C_{15}H_{21}N_3O_2S$ 309.1511, found 309.1512.

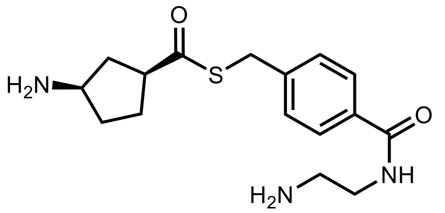

S-(4-((2-aminoethyl)carbamoyl)benzyl) (1S,3R)-3-aminocyclopentane-1-carbothioate (13). Prepared according to general procedure C using (1S,3R)-3-((tert-butoxycarbonyl)amino)cyclopentane-1-carboxylic (98.6 mg, 0.43 mmol), 1-ethyl-3-(3-dimethylaminopropyl) carbodiimide hydrochloride (EDCI) (165.1 mg, 0.86 mmol), dimethylamino pyridine (105.2 mg, 0.86 mmol), t*ert*-butyl (2-(4-(mercaptomethyl)benzamido)ethyl) carbamate (145 mg, 0.43 mmol). The product was obtained as a white powder (91.4 mg, 66%). Silica gel column chromatography [Solvent System: Hexanes-Ethyl Acetate; 1:1, Rf= 0.1]. ^{1}H NMR (500 MHz, Methanol-d_4) δ 7.81 (d, J = 8.3 Hz, 2H), 7.41 (d, J = 8.2 Hz, 2H), 4.20 (s, 2H), 3.23 (p, J = 8.0 Hz, 1H), 3.15 (t, J = 6.0 Hz, 3H), 2.35 (dt, J = 13.5, 7.8 Hz, 1H), 2.16 – 2.08 (m, 1H), 2.08 – 2.02 (m, 1H), 2.02 – 1.93 (m, 1H), 1.89 (dt, J = 13.6, 7.8 Hz, 2H), 1.78 – 1.66 (m, 1H), 1.40 (d, J = 9.6 Hz, 2H). ^{13}C NMR (126 MHz, DMSO-d_6) δ 199.88, 166.31, 141.31, 132.80, 128.53, 127.76, 50.54, 50.35, 38.51, 37.09, 34.12, 31.91, 29.70, 27.39. HRMS (m/z): $[M]^+$ calcd. for $C_{16}H_{25}N_3O_2S$ 323.1667, found 322.1669.

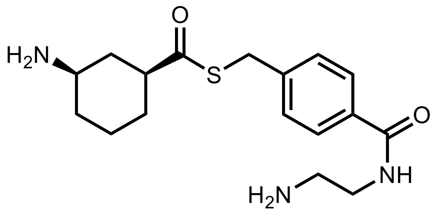

S-(4-((2-aminoethyl)carbamoyl)benzyl) (1S,3R)-3-aminocyclohexane-1-carbothioate (14). Prepared according to general procedure C using (1S,3R)-3-((tert-butoxycarbonyl)amino)cyclohexane-1-carboxylic acid (104.6 mg, 0.43 mmol), 1-ethyl-3-(3-dimethylaminopropyl) carbodiimide hydrochloride (EDCI) (165.1 mg, 0.86 mmol), dimethylamino pyridine (105.2 mg, 0.86 mmol), t*ert*-butyl (2-(4-(mercaptomethyl)benzamido)ethyl) carbamate (145 mg, 0.43 mmol). The product was obtained as a white powder (99.7 mg, 69%). Silica gel column chromatography [Solvent System: Hexanes-Ethyl Acetate; 1:1, Rf= 0.1]. ^{1}H NMR (500 MHz, Methanol-d_4) δ 7.80 (d, J = 8.3 Hz, 2H), 7.39 (d, J = 8.3 Hz, 2H), 4.17 (s, 2H), 3.64 (t, J = 5.9 Hz, 2H), 3.15 (t, J = 5.9 Hz, 3H), 2.73 (tt, J = 3.4 Hz, 1H), 2.20 (d, J = 12.4 Hz, 1H), 2.09 – 1.86 (m, 3H), 1.62 – 1.24 (m, 4H). ^{13}C NMR (126 MHz, DMSO-d_6) δ 201.22, 166.83, 141.85, 133.30, 129.00, 128.23, 46.49,

46.37, 39.04, 37.59, 32.23, 31.04, 28.78, 28.00, 19.92. HRMS (m/z): $[M]^+$ calcd. for $C_{17}H_{27}N_3O_2S$ 337.1824, found 337.1824.

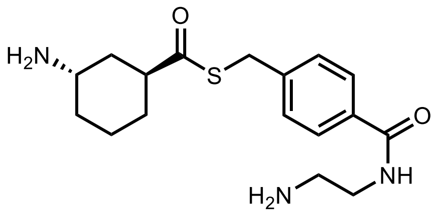

S-(4-((2-aminoethyl)carbamoyl)benzyl) (1S,3S)-3-aminocyclohexane-1-carbothioate (15). Prepared according to general procedure C using (1S,3S)-3-((tert-butoxycarbonyl)amino)cyclohexane-1-carboxylic acid 104.1 mg, 0.43 mmol), 1-ethyl-3-(3-dimethylaminopropyl) carbodiimide hydrochloride (EDCI) (165.1 mg, 0.86 mmol), dimethylamino pyridine (105.2 mg, 0.86 mmol), t*ert*-butyl (2-(4-(mercaptomethyl)benzamido)ethyl) carbamate (145 mg, 0.43 mmol). The product was obtained as a yellow powder (95.4 mg, 62%). Silica gel column chromatography [Solvent System: Hexanes-Ethyl Acetate; 1:1, Rf= 0.1]. ^{1}H NMR (500 MHz, DMSO-d_6) δ 8.77 (t, *J* = 5.5 Hz, 1H), 8.18 (s, 6H), 7.82 (d, *J* = 8.4 Hz, 2H), 7.31 (d, *J* = 8.3 Hz, 2H), 4.11 (s, 2H), 3.46 (q, *J* = 6.0 Hz, 2H), 3.26 – 3.18 (m, 1H), 3.08 (t, *J* = 5.7 Hz, 1H), 2.92 (t, *J* = 6.1 Hz, 2H), 2.44 (p, *J* = 1.8 Hz, 1H), 1.96 (ddd, *J* = 13.5, 7.0, 4.0 Hz, 1H), 1.71 (dtd, *J* = 12.8, 8.2, 4.2 Hz, 1H), 1.61 (d, *J* = 5.6 Hz, 2H), 1.46 (qt, *J* = 7.9, 2.9 Hz, 1H), 1.33 (dtt, *J* = 12.8, 8.8, 4.2 Hz, 1H). ^{13}C NMR (126 MHz, DMSO-d_6) δ 200.74, 166.35, 141.37, 132.82, 128.52, 127.75, 46.01, 45.89, 38.56, 37.11, 31.75, 30.56, 28.30, 27.52, 19.44. HRMS (m/z): $[M]^+$ calcd. for $C_{17}H_{27}N_3O_2S$ 337.1824, found 337.1824.

Preparation of DNA templates for RNAs

The DNA templates for flexizyme and tRNAs preparation were synthesized by using the following primers as previously described[3].

- Sequence of the final DNA templates used for *in vitro* transcription by the T7 RNA polymerase

fMet<br>(CAU)	GTAATACGACTCACTATAGGCGGGGTGGAGCAGCCTGGTAGCTCGTCGGGCTC<br>ATAACCCGAAGATCGTCGGTTCAAATCCGGCCCCCGCAACCA
Pro1E2<br>(GGU)	GTAATACGACTCACTATAGGGTGATTGGCGCAGCCTGGTAGCGCACTTCGTTGG<br>TAACGAAGGGGTCAGGGGTTCGAATCCCCTATCACCCGCCA

*Note that the underlined sequences are the T7 promoter sequence.

Preparation of Fx and tRNAs

Flexizymes and tRNAs were prepared using the HiScribeTM T7 High yield RNA synthesis kit (NEB, E2040S) and purified by the previously reported methods[3].

Supplementary Figures

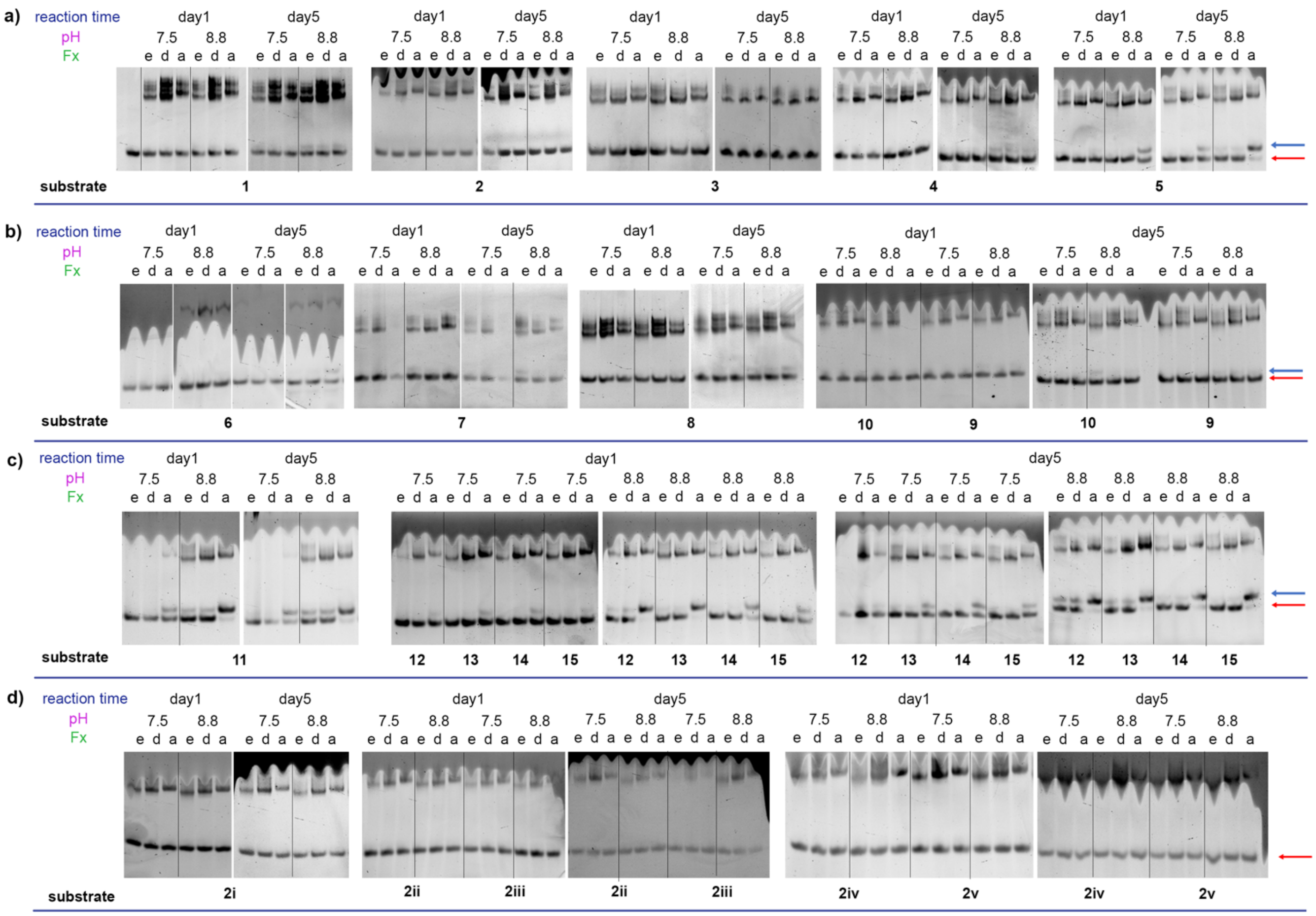

Supplementary Figure 1. Acylation of microhelix with substrates 1-15 and 2i-2v. (**a-d**) The Fx-catalyzed acylation reaction using the 20 substrates were monitored at two different pH (7.5 and 8.8) over 120 h with three different flexizymes (eFx, dFx, and aFx). Fx: Flexizyme (43-45 nt), mihx: microhelix (22 nt). The yield of each reaction was determined by quantifying the relative band intensity of unacylated (red arrow) and acylated microhelix (blue arrow) on the gel using ImageJ software. Substrate structures for **1-15** and **2i-2v** are shown in the characterization data above. Data are representative of multiple (n=1-3) independent experiments.

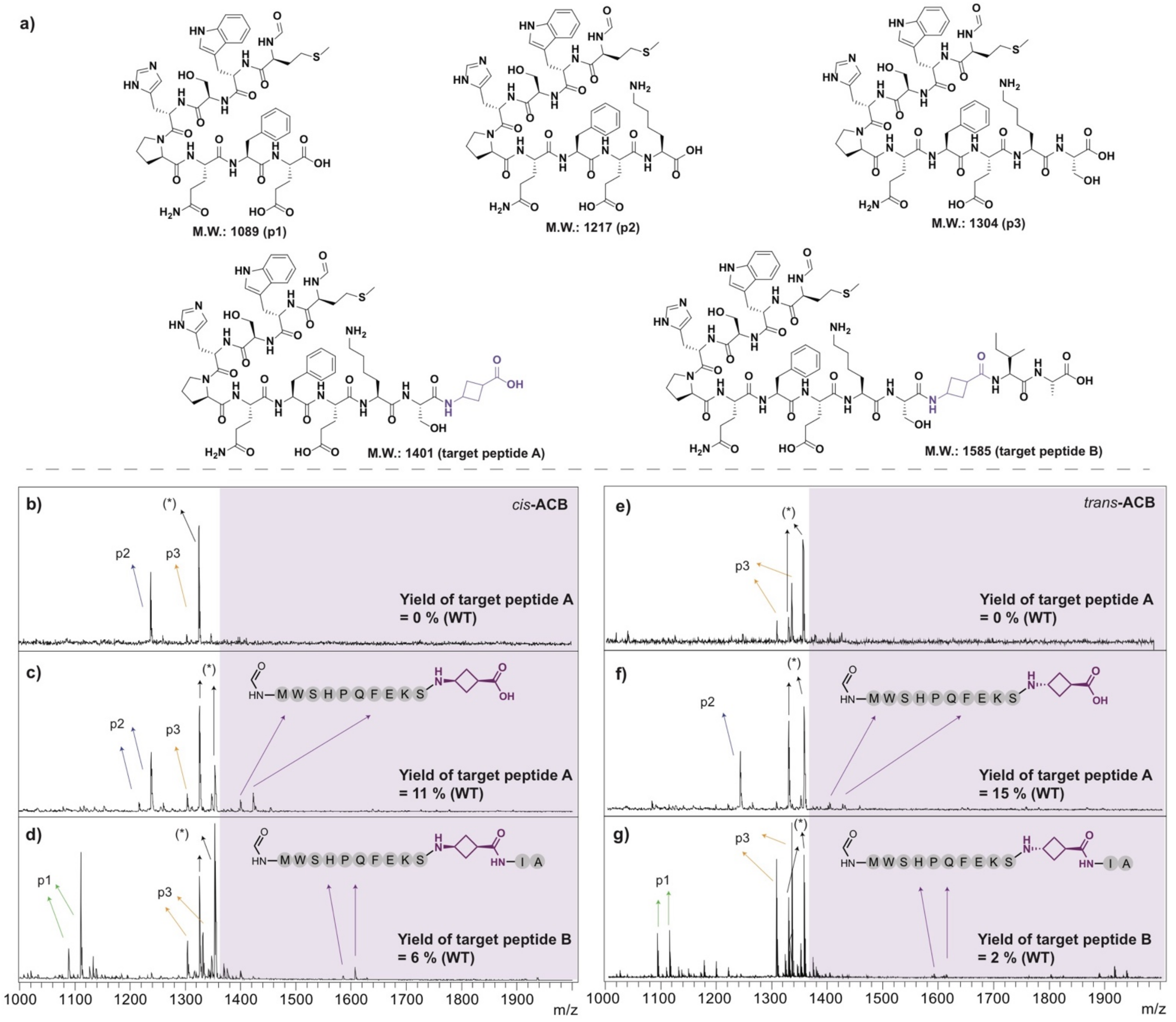

Supplementary Figure 2. Characterization of the C-terminus functionalized peptide with *cis*- and *trans*-ACB (11-12).

(**a**) Structure and molecular weight of target and byproduct truncated peptides in the PURExpress^{TM} translation reaction that are produced. (**b**) MALDI-TOF mass spectrometry data from attempt to incorporate *cis*-ACB (**11**) with wild-type ribosomes. (**c**) Addition of the Hecht ribosomes (040329) under the same PURExpress^{TM} reaction conditions carried out in **b** yielded a peak that corresponds to the theoretical mass of a target peptide containing *cis*-ACB into the C-terminus. (**d**) Additional amino acids, Ile and Ala, can be elongated after the incorporation of **11**, suggesting the engineered ribosome enabled site-specific incorporation. (**e**) MALDI-TOF data from attempt to incorporate *trans*-ACB (1**2**) with wild-type ribosomes. (**f**) Addition of the Hecht ribosomes under the same conditions carried out in **e** yielded a peak corresponding to the theoretical mass of a target peptide containing **12** into the C-terminus. (**g**) The same additional amino acid residues (Ile and Ala) are elongated after the incorporation of **12**.

The theoretical mass of the truncated peptides is $[M+H]^+$ = 1089; $[M+Na]^+$ = 1111 (green arrows) for **p1,** $[M+H]^+$ = 1217; $[M+Na]^+$ = 1239 (blue arrows) for **p2**, and $[M+H]^+$ = 1304; $[M+Na]^+$ = 1326 (orange arrows) for **p3**. The

marked peaks by an asterisk ($[M+H]^+$ = 1334; $[M+Na]^+$ = 1356, black arrows) were unidentified. The highlighted (purple) area was used to produce **Fig. 5b, c** and **e-h.** The percent yield of the target peptide was determined based on the relative peak area (PA) of a target polypeptide over a total amount of the truncated and target polypeptides (i.e., relative yield (%) = Σ of PA (target peptide) / Σ of PA (**P1** + **P2** + **P3** + target peptide) × 100). Data are representative of three independent experiments.

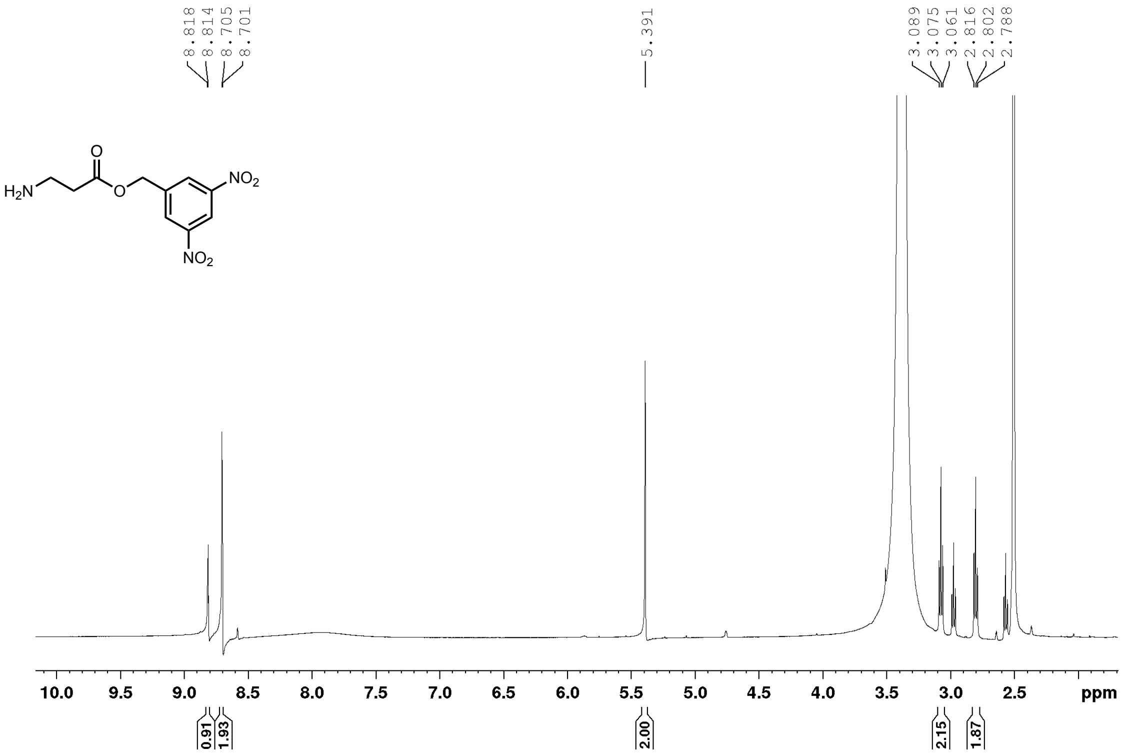

Supplementary Figure 3. ^{1}H NMR (500 MHz, DMSO-*d$_6$*) of 1.

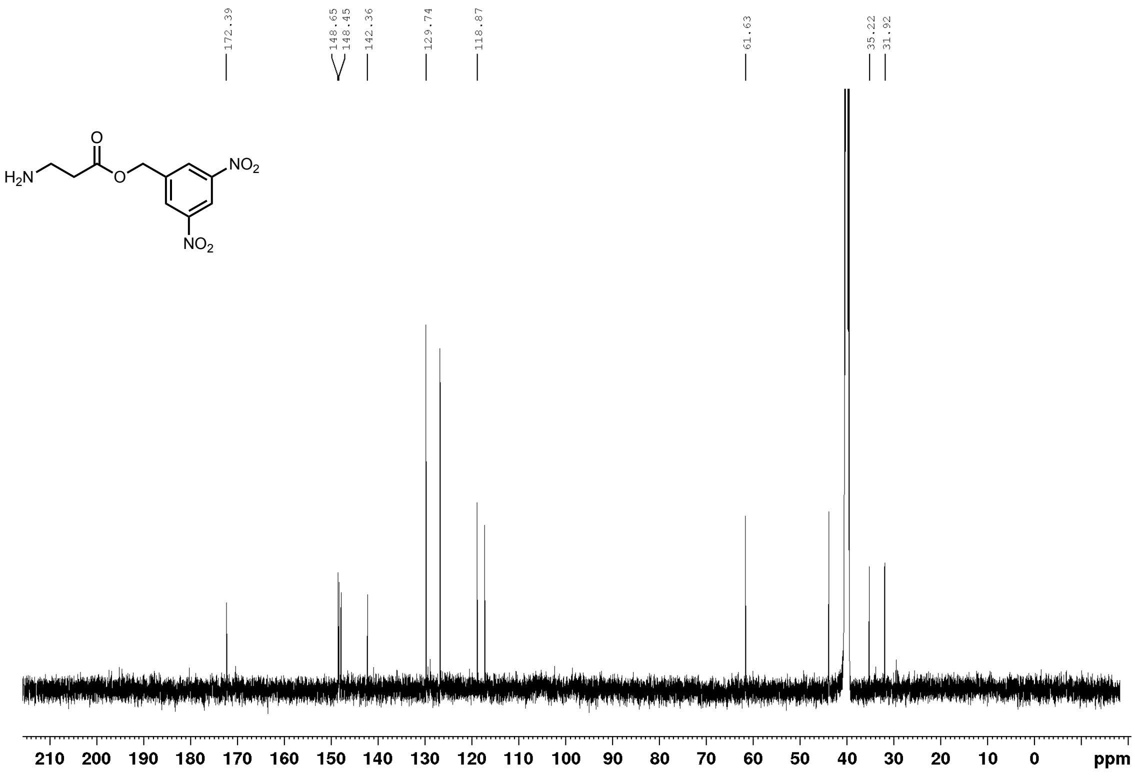

Supplementary Figure 4. ^{13}C NMR (125 MHz, DMSO-*d$_6$*) of 1.

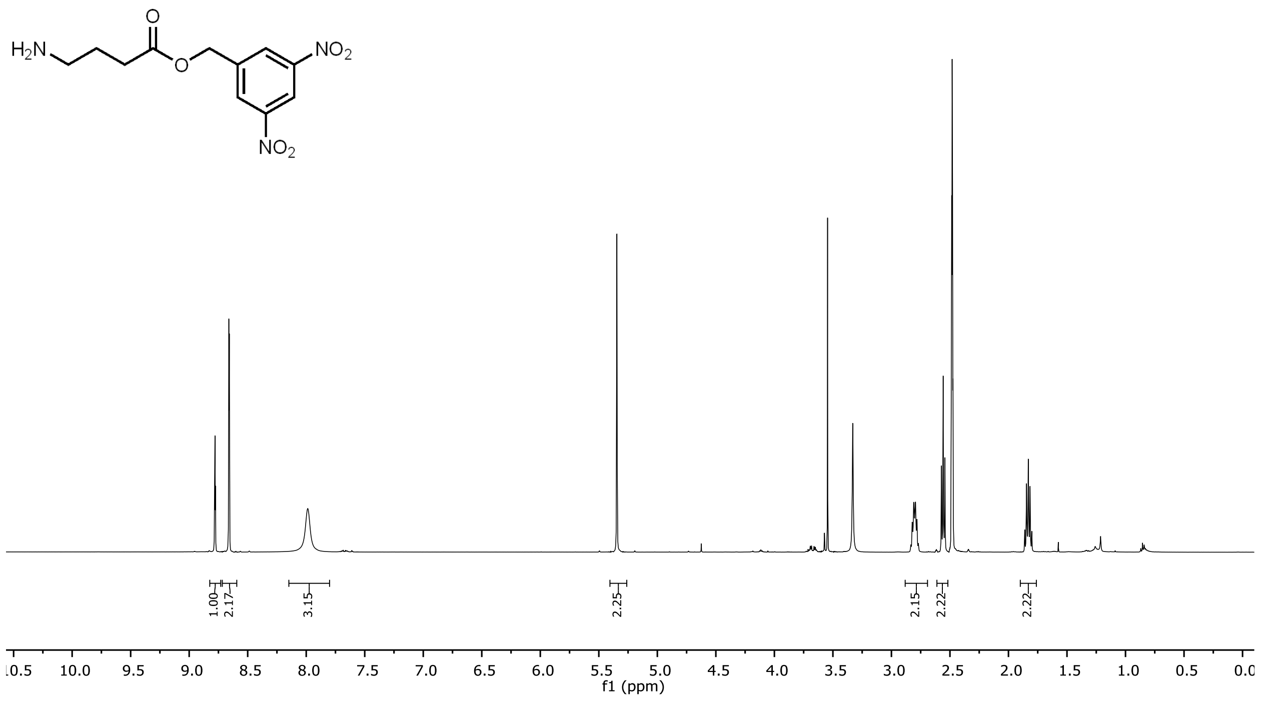

Supplementary Figure 5. ^{1}H NMR (500 MHz, DMSO-*d*$_6$) of 2.

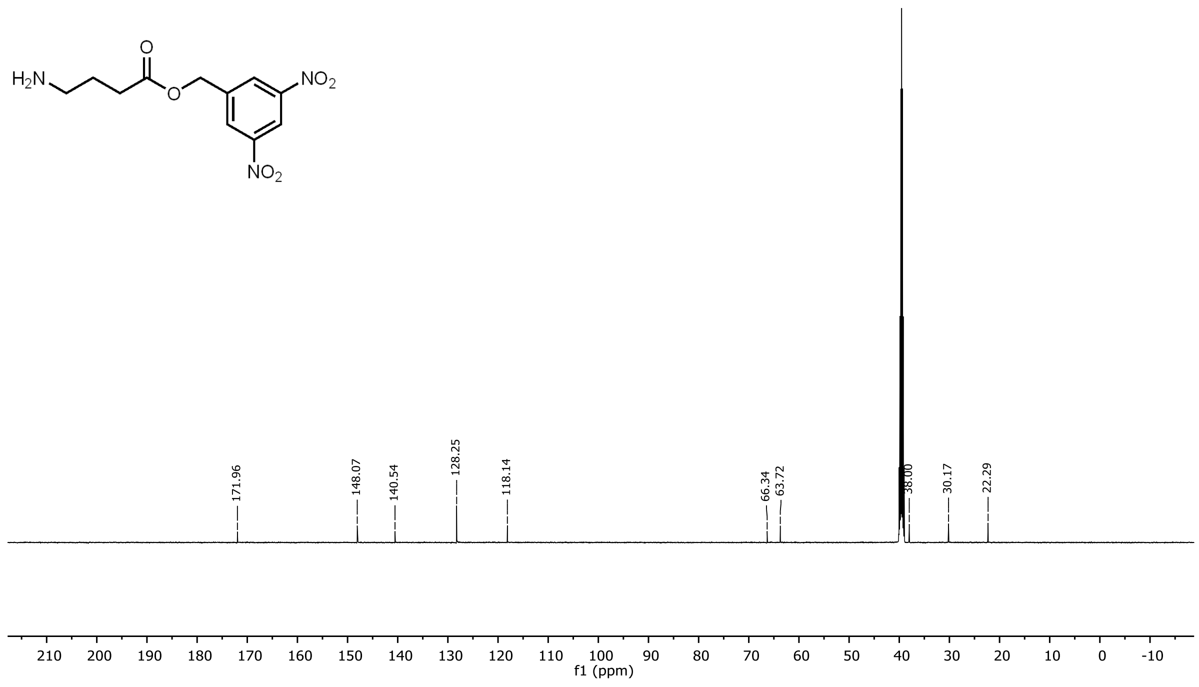

Supplementary Figure 6. ^{13}C NMR (125 MHz, DMSO-*d*$_6$) of 2.

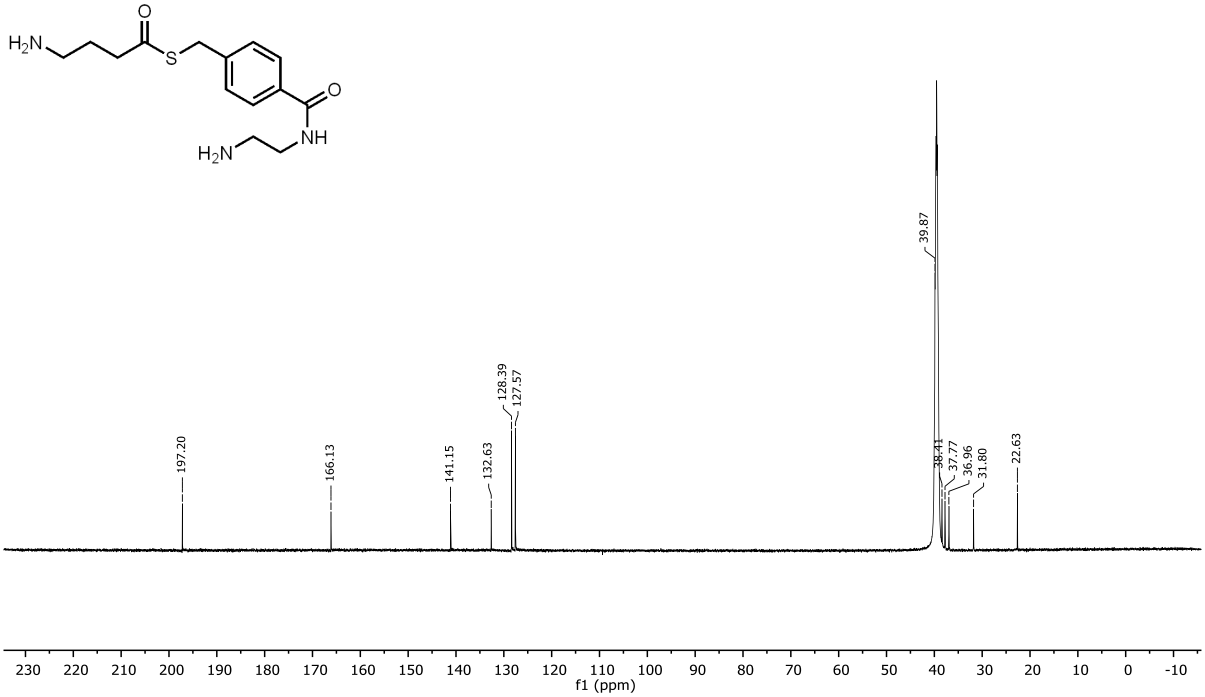

Supplementary Figure 7. ^{1}H NMR (500 MHz, DMSO-d_6) of 2i.

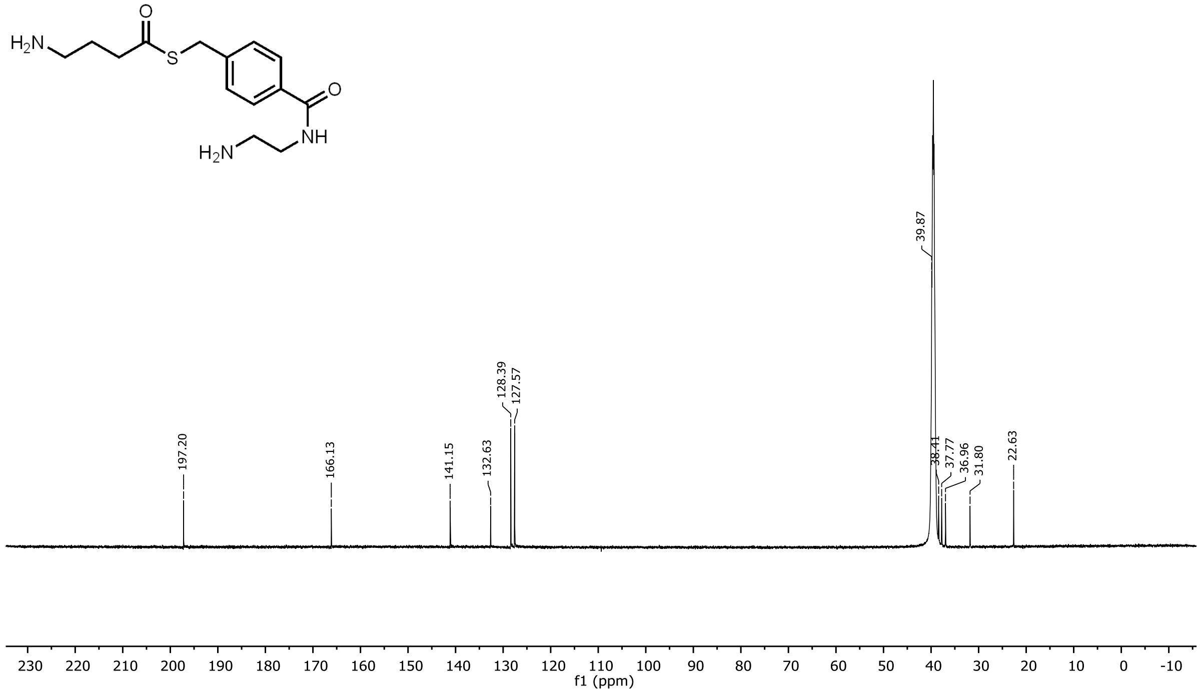

Supplementary Figure 8. ^{13}C NMR (125 MHz, DMSO-d_6) of 2i.

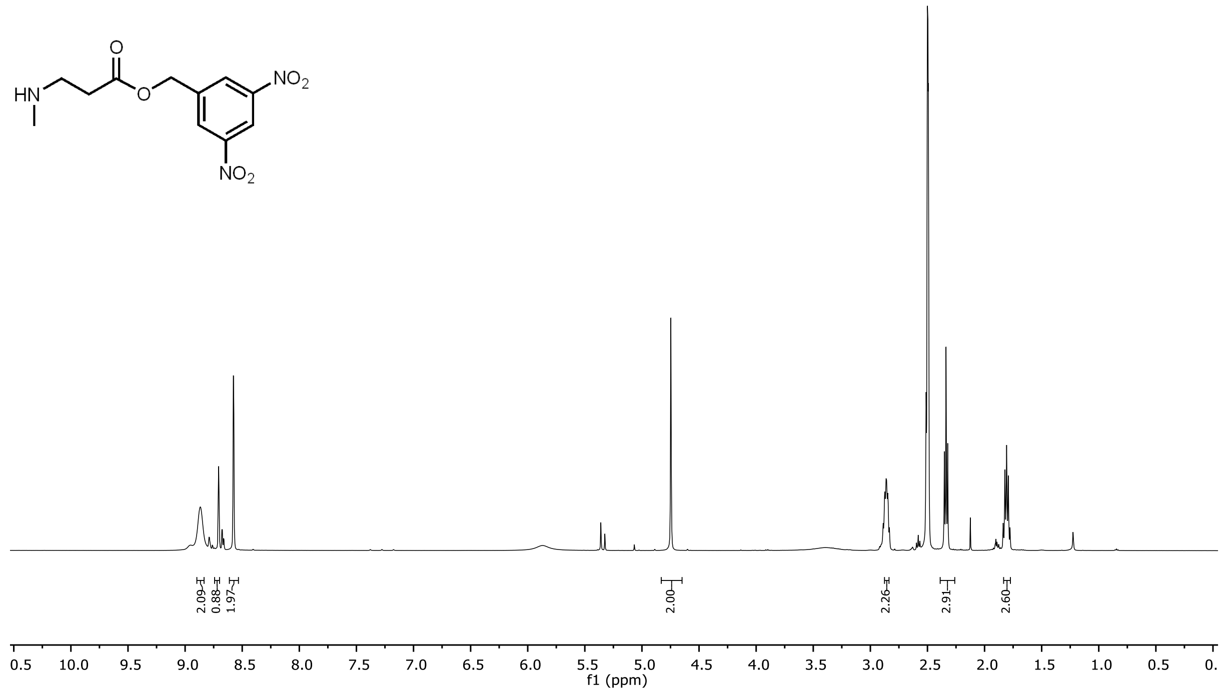

Supplementary Figure 9. ^{1}H NMR (500 MHz, DMSO-*d$_6$*) of 2ii.

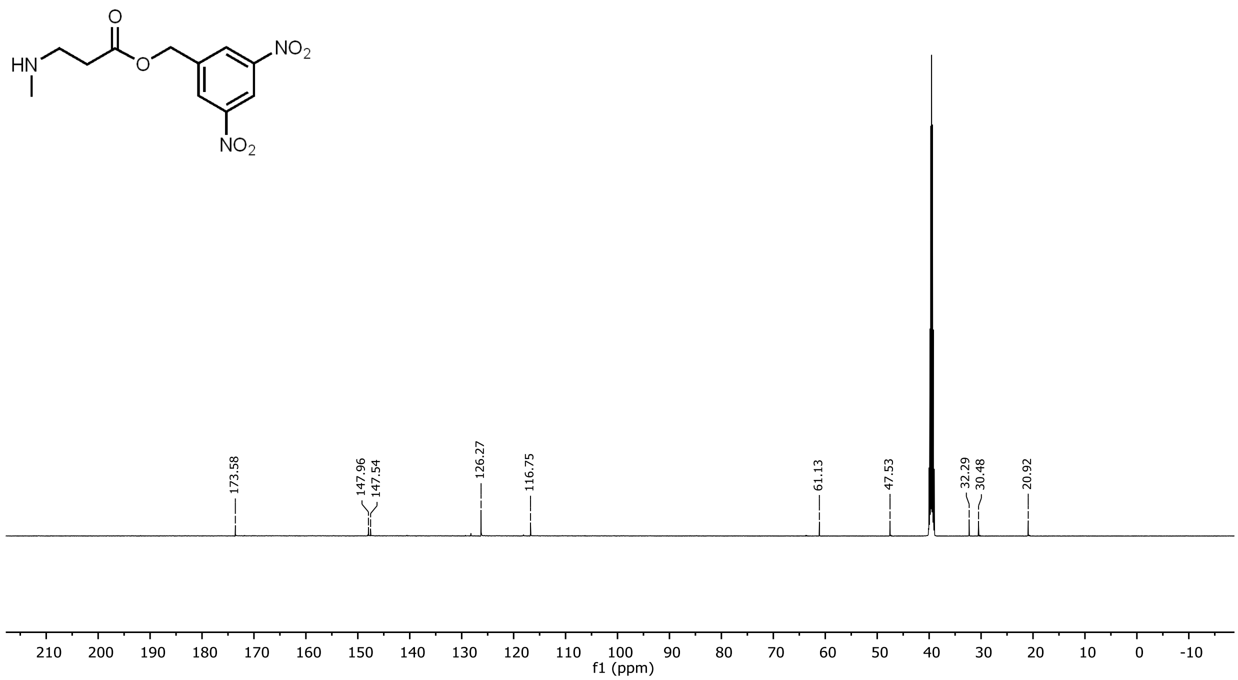

Supplementary Figure 10. ^{13}C NMR (125 MHz, DMSO-*d$_6$*) of 2ii.

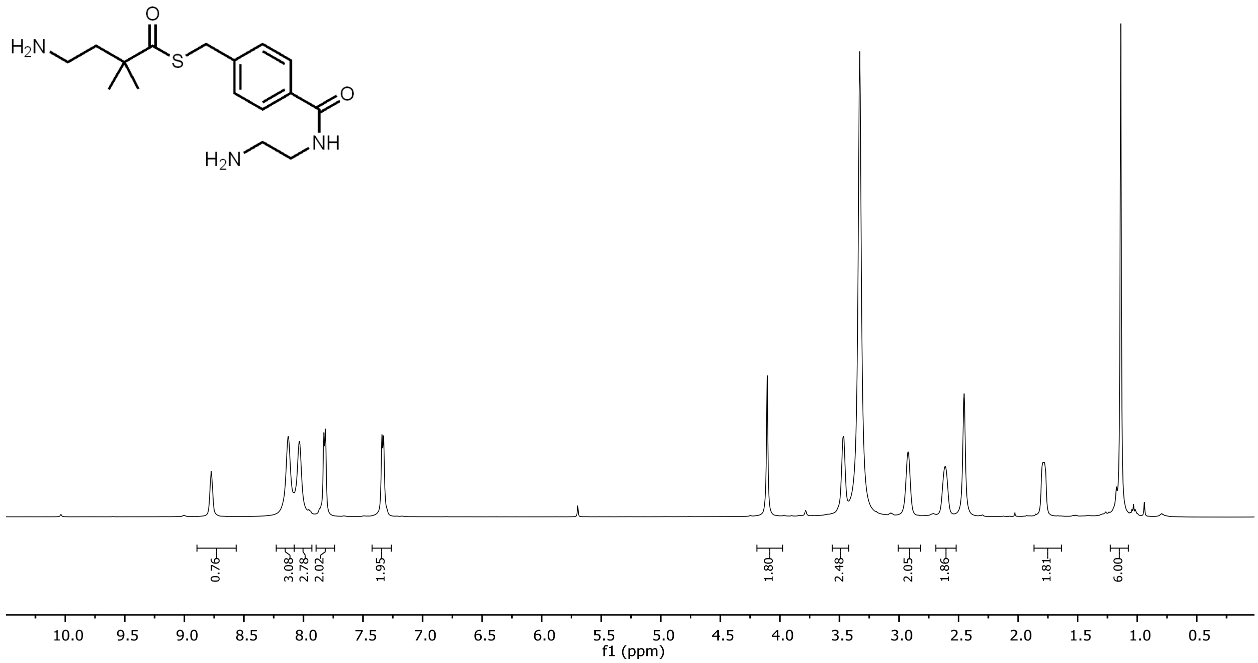

Supplementary Figure 11. ^{1}H NMR (500 MHz, DMSO-*d*$_6$) of 2iii.

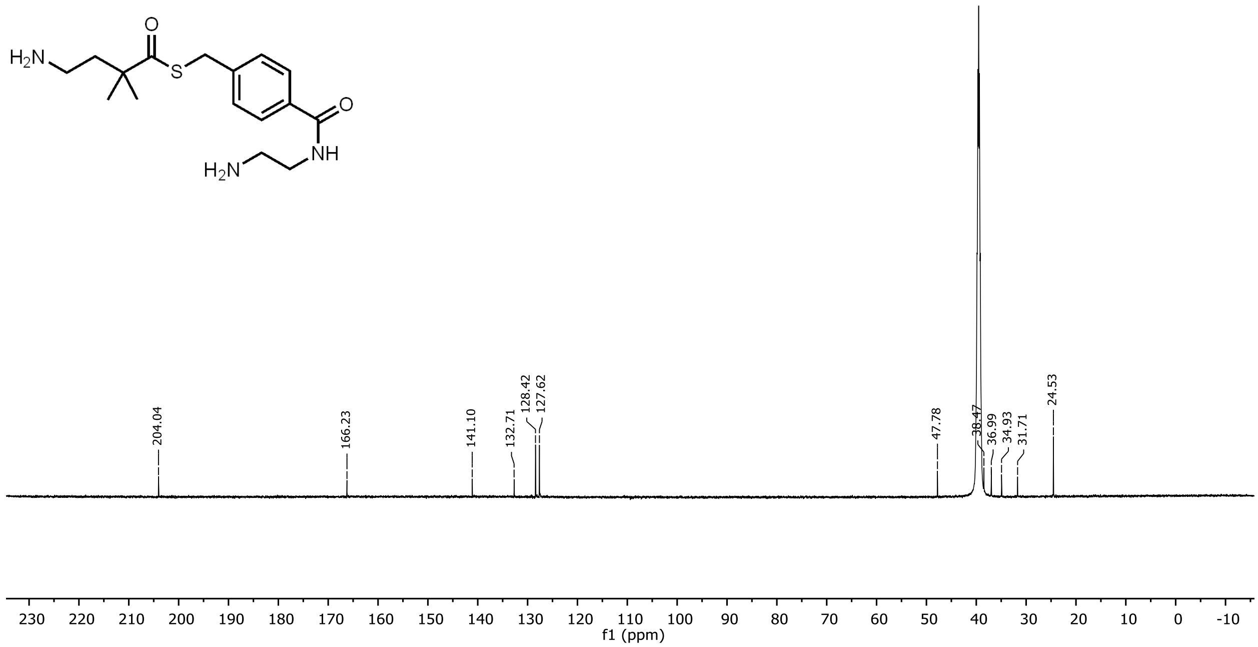

Supplementary Figure 12. ^{13}C NMR (125 MHz, DMSO-*d*$_6$) of 2iii.

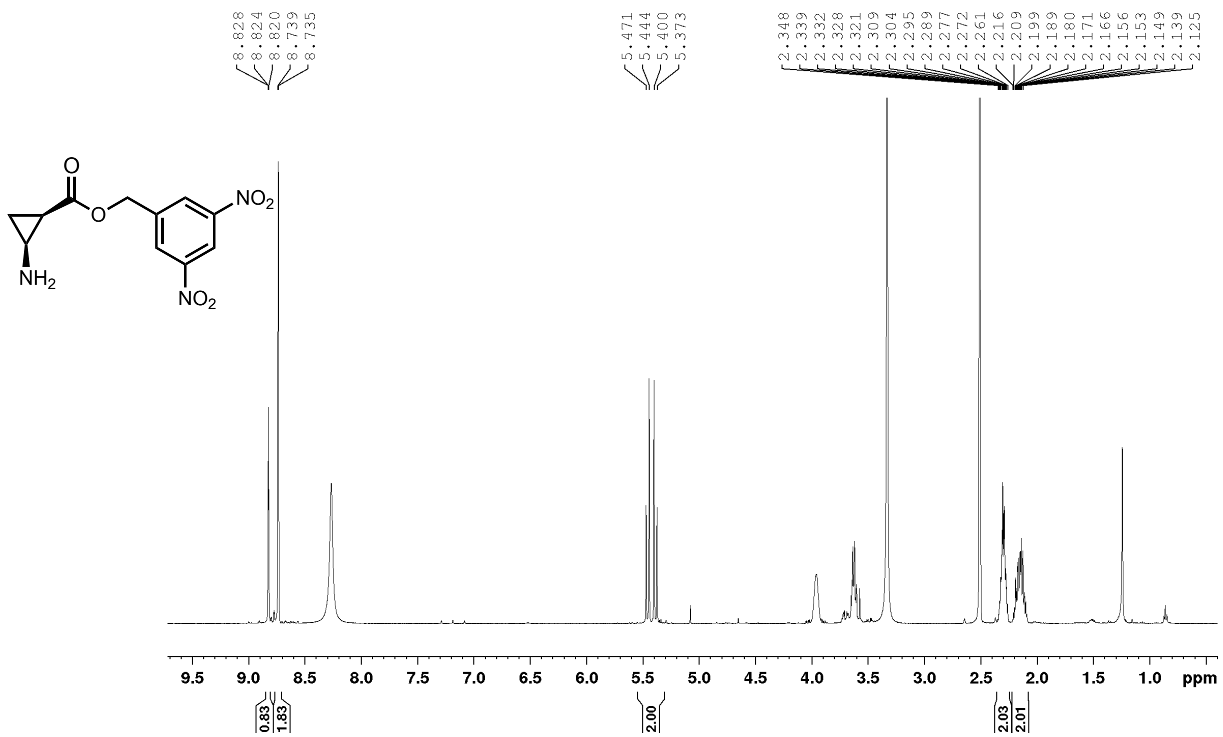

Supplementary Figure 13. ^{1}H NMR (500 MHz, DMSO-d_6) of 2iv.

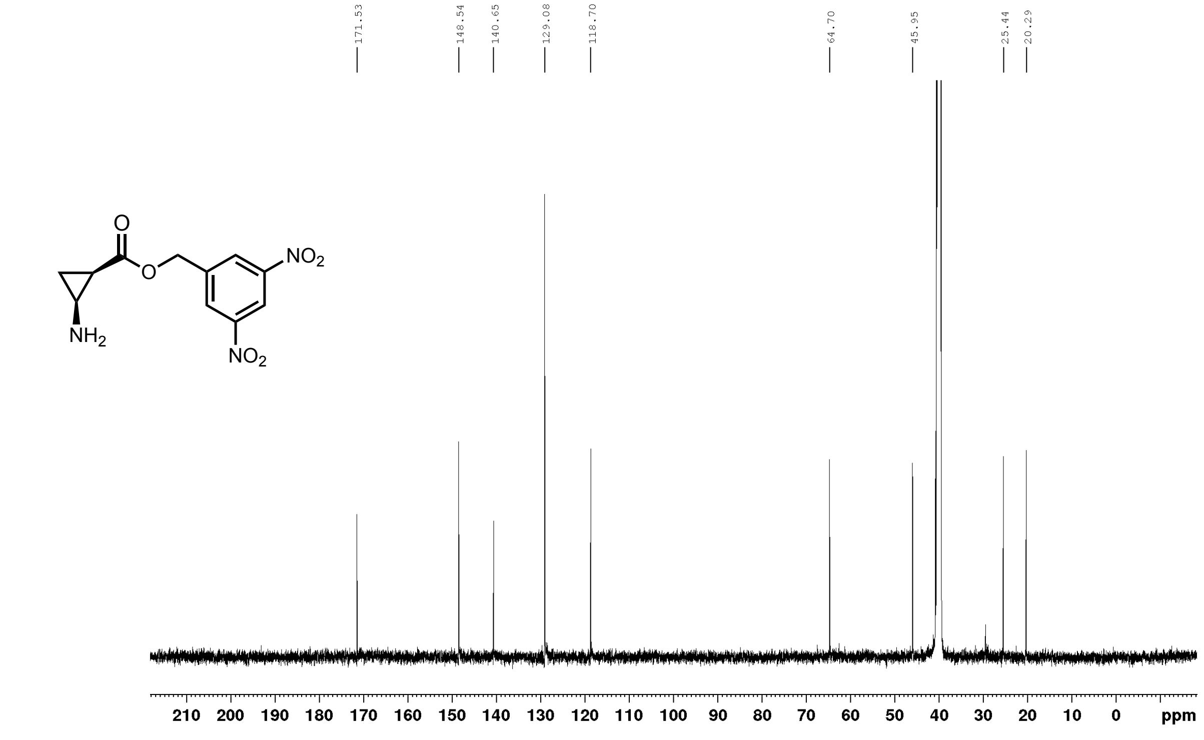

Supplementary Figure 14. ^{13}C NMR (125 MHz, DMSO-d_6) of 2iv.

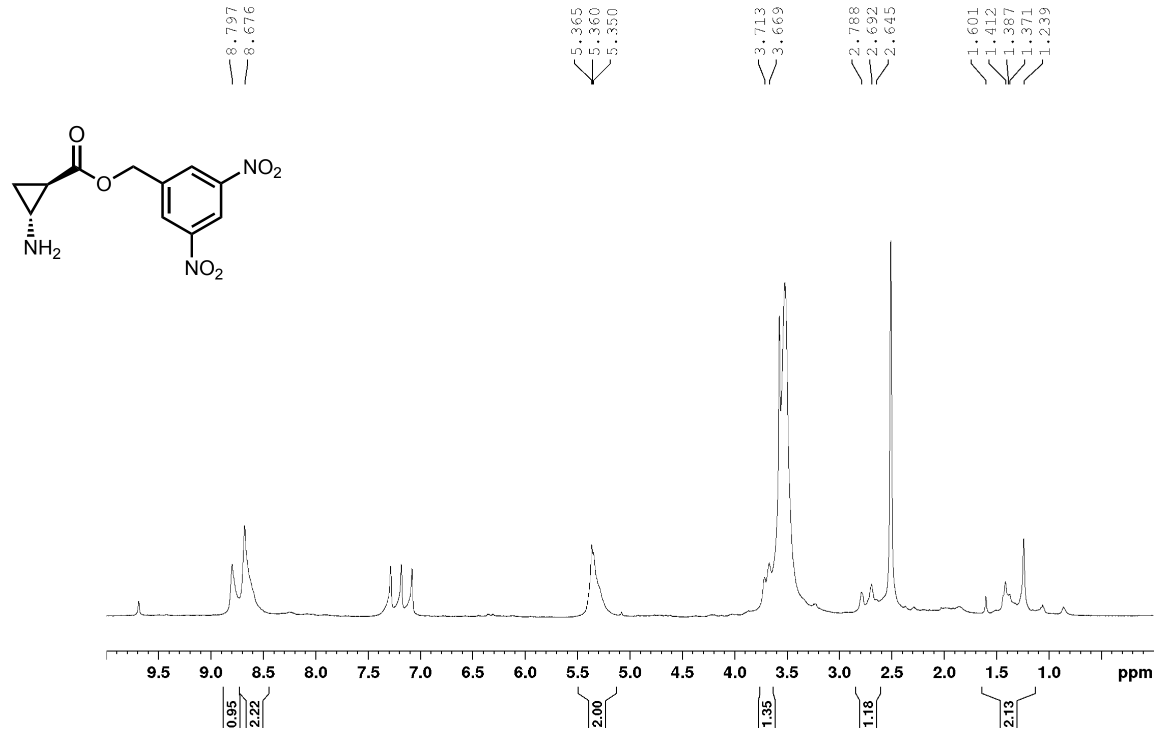

Supplementary Figure 15. **^{1}H NMR (500 MHz, DMSO-*d*$_6$) of 2v.**

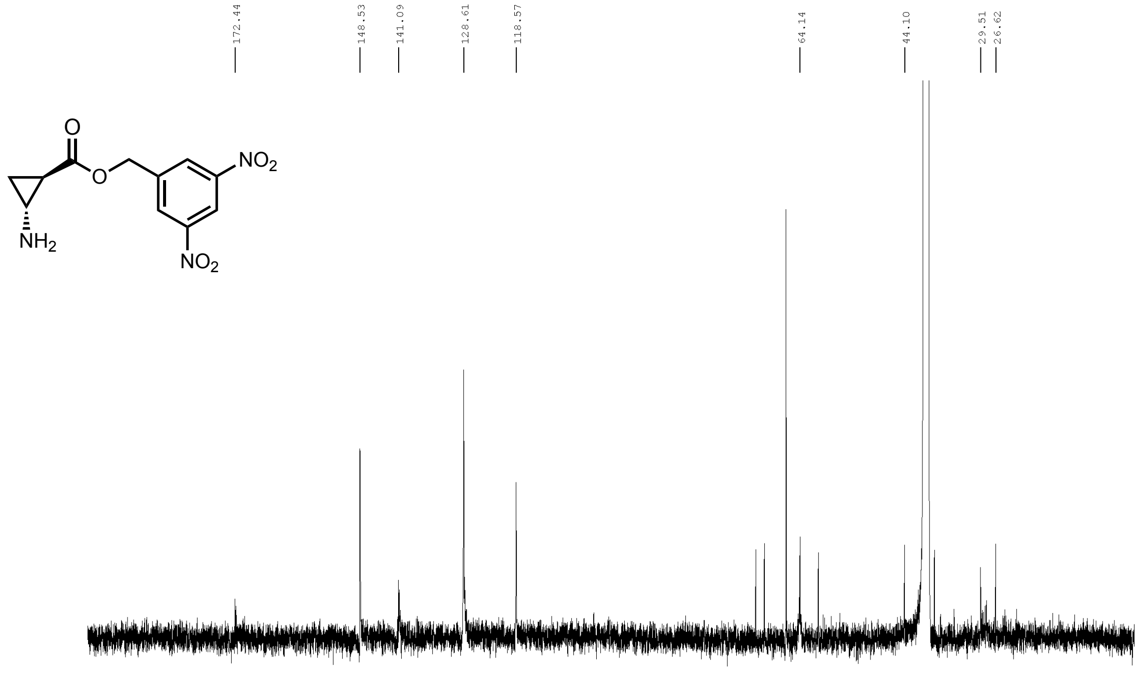

Supplementary Figure 16. **^{13}C NMR (125 MHz, DMSO-*d*$_6$) of 2v.**

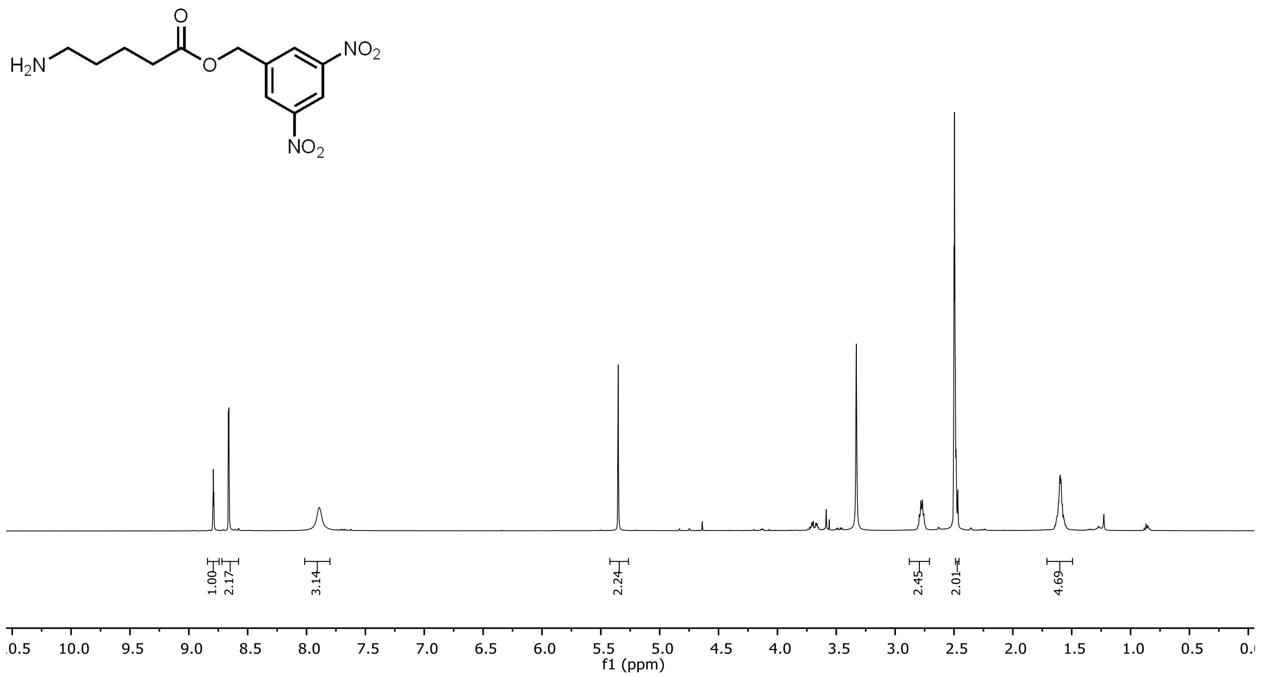

Supplementary Figure 17. ^{1}H NMR (500 MHz, DMSO-*d$_6$*) of 3.

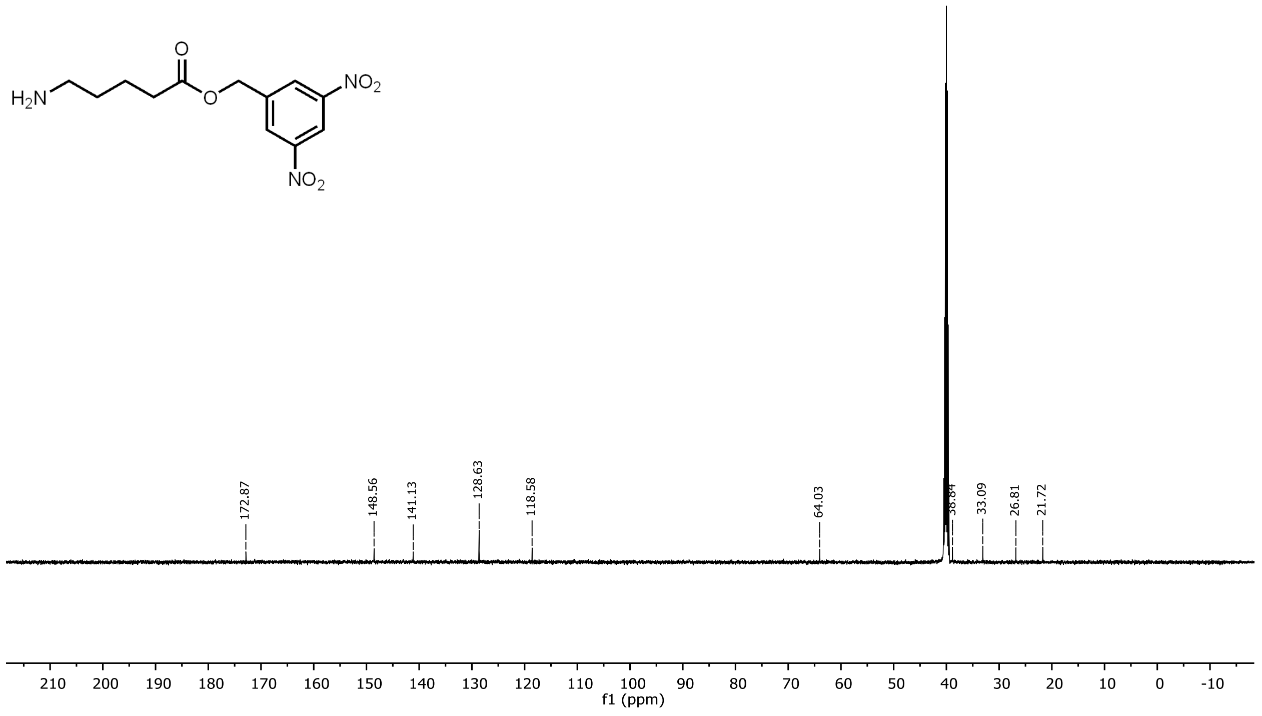

Supplementary Figure 18. ^{13}C NMR (125 MHz, DMSO-*d$_6$*) of 3.

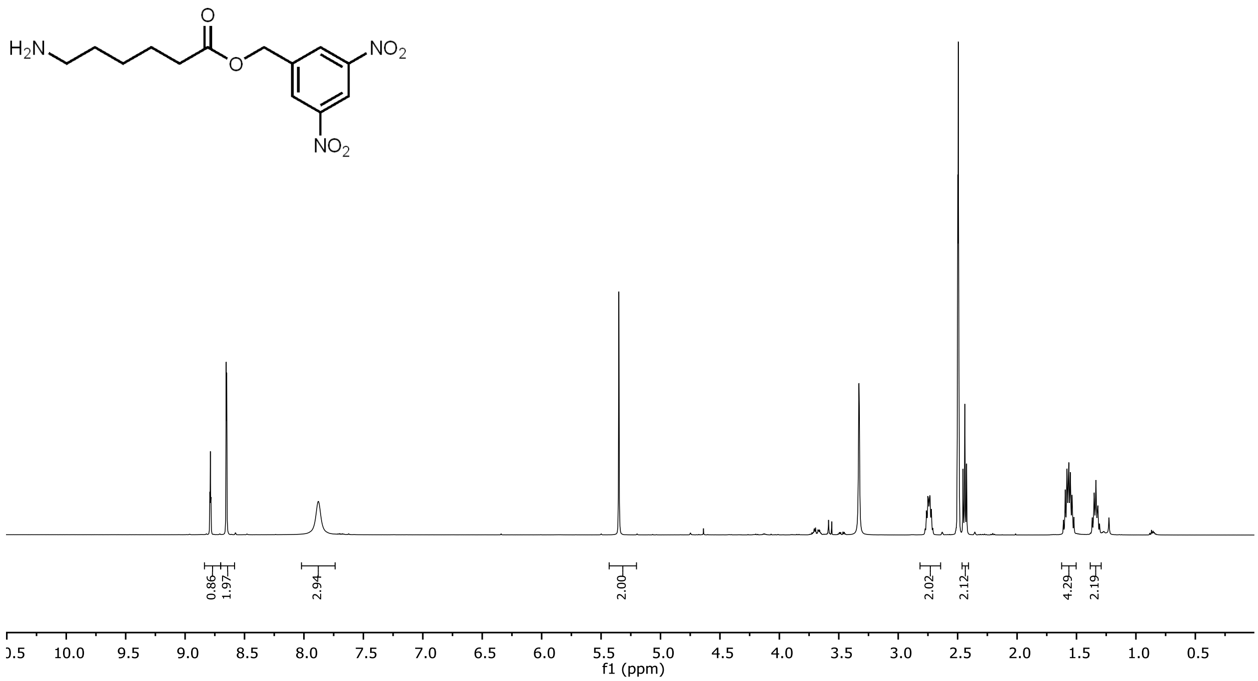

Supplementary Figure 19. ^{1}H NMR (500 MHz, DMSO-d_6) of 4.

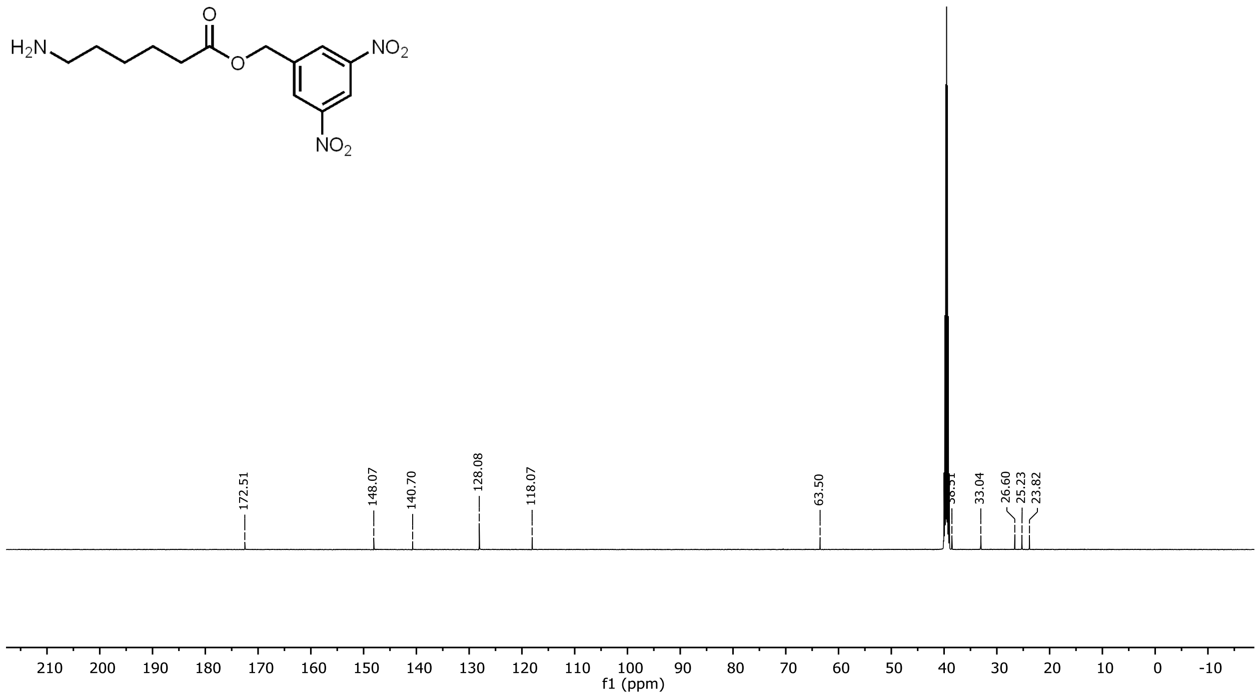

Supplementary Figure 20. ^{13}C NMR (125 MHz, DMSO-d_6) of 4.

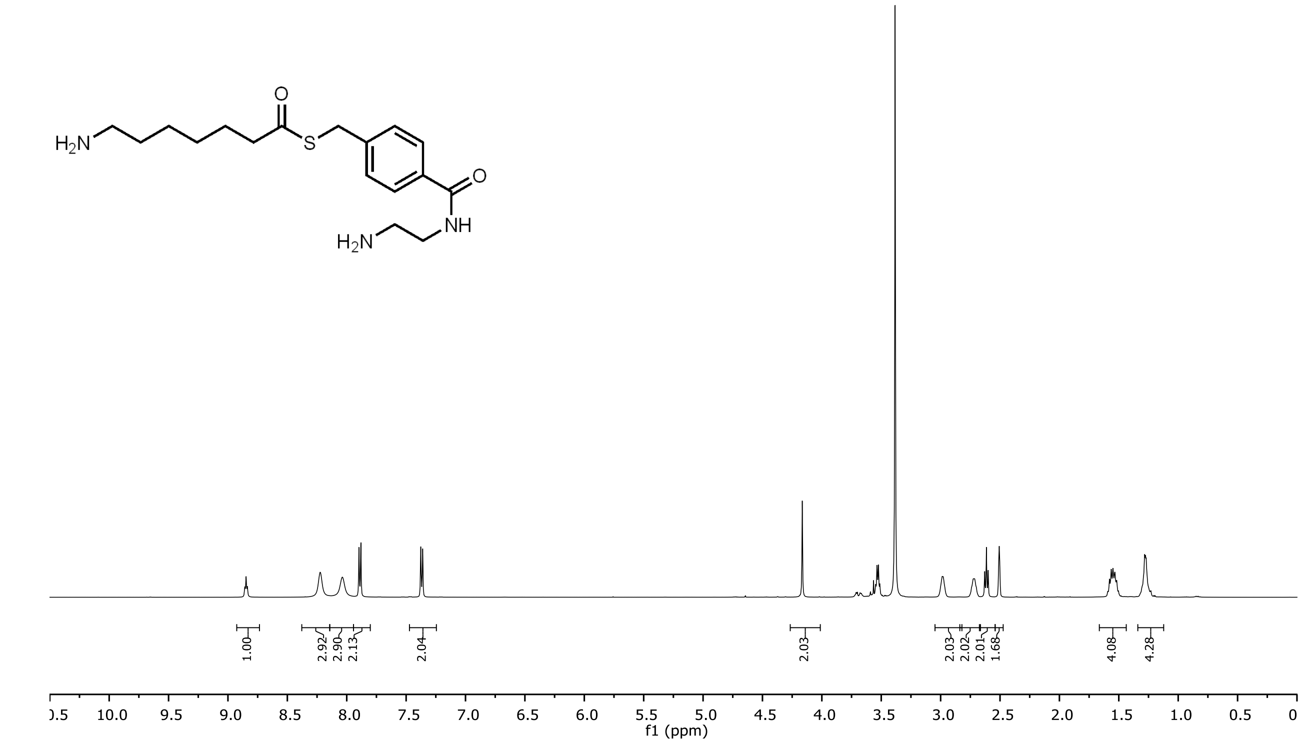

Supplementary Figure 21. ^{1}H NMR (500 MHz, DMSO-*d$_6$*) of 5.

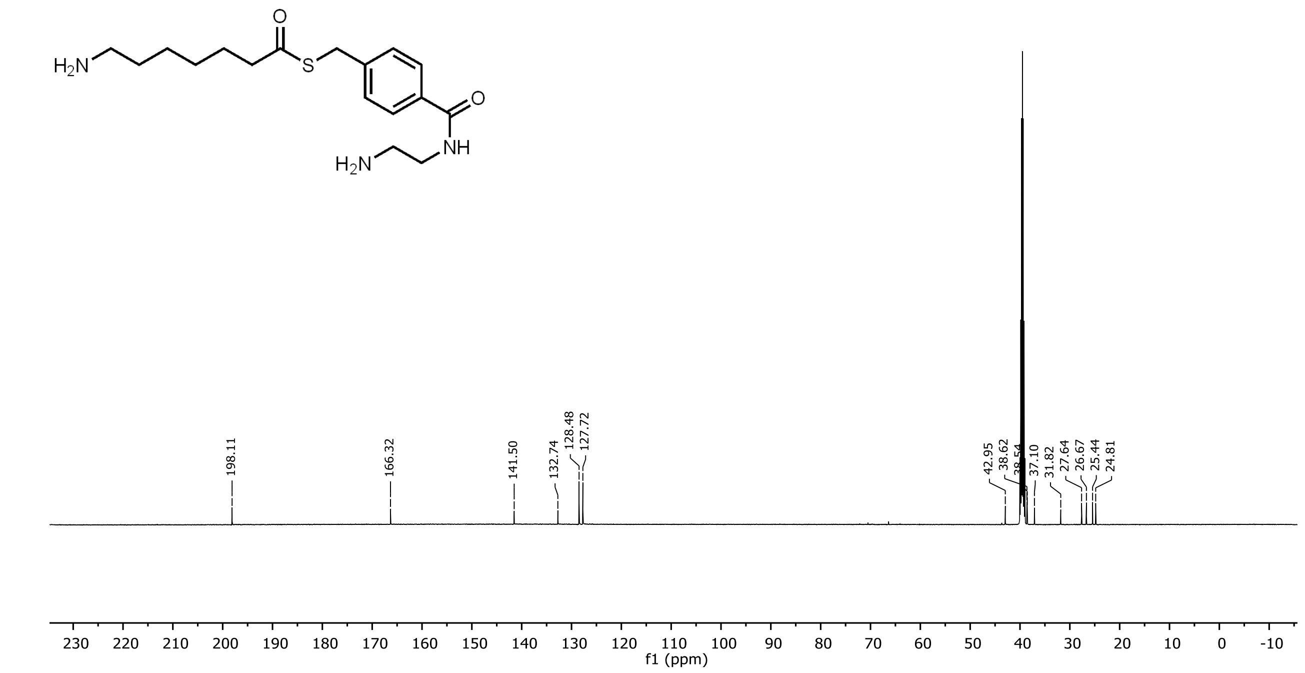

Supplementary Figure 22. ^{13}C NMR (125 MHz, DMSO-*d$_6$*) of 5.

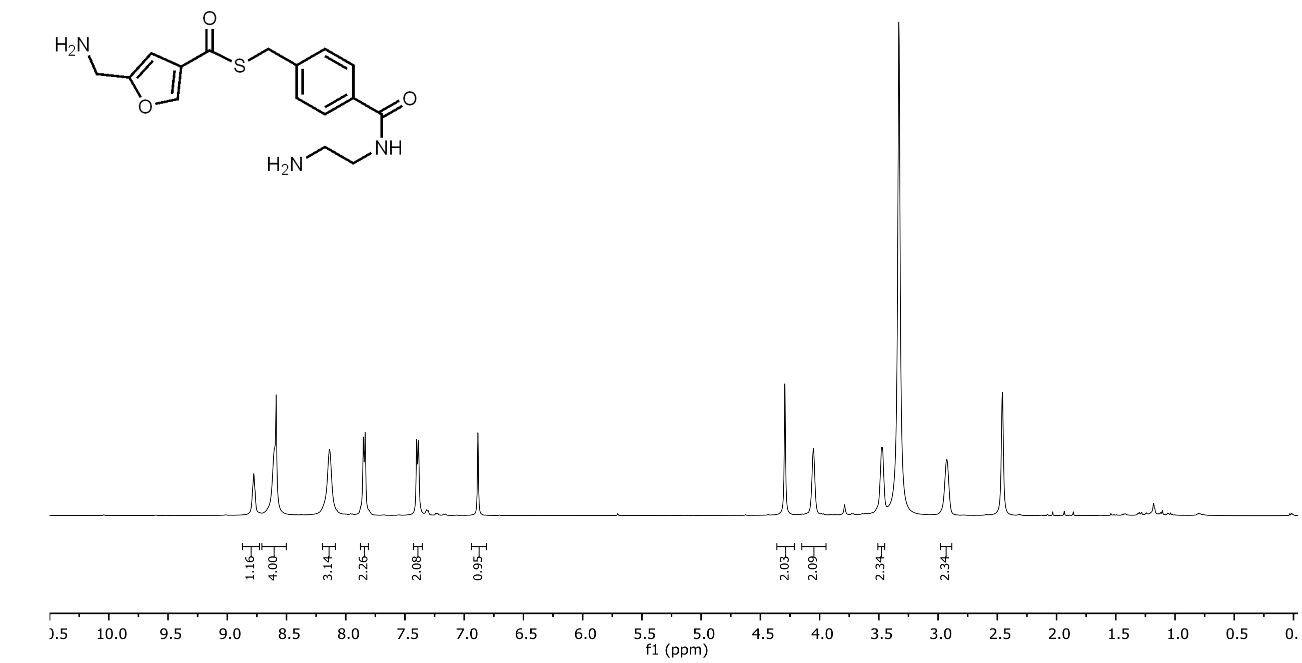

Supplementary Figure 23. ^{1}H NMR (500 MHz, DMSO-*d$_6$*) of 6.

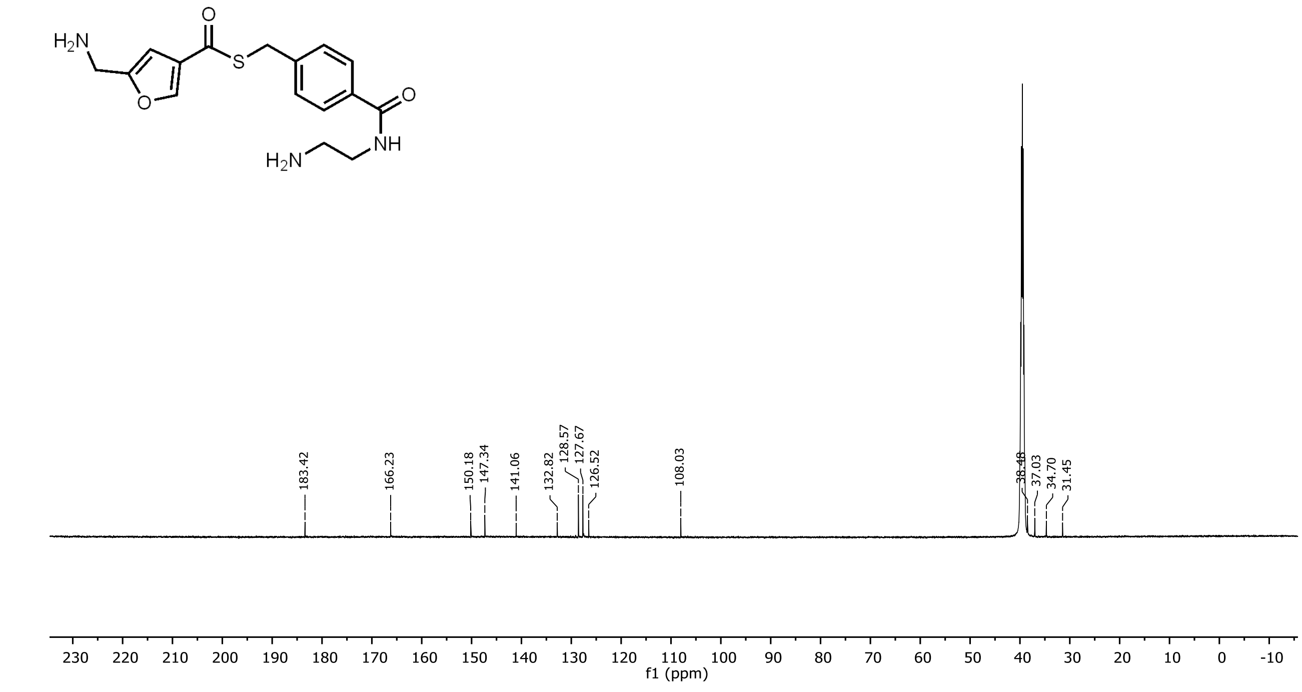

Supplementary Figure 24. ^{13}C NMR (125 MHz, DMSO-*d$_6$*) of 6.

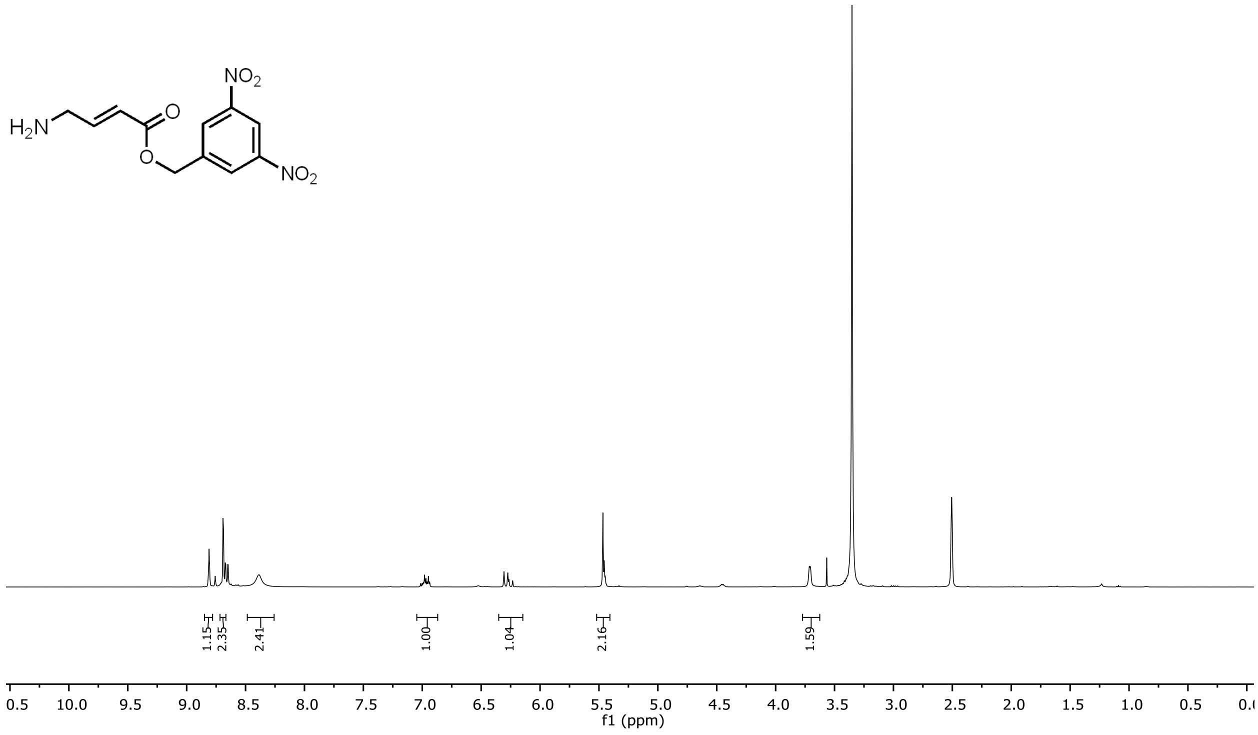

Supplementary Figure 25. ^{1}H NMR (500 MHz, DMSO-*d*$_6$) of 7.

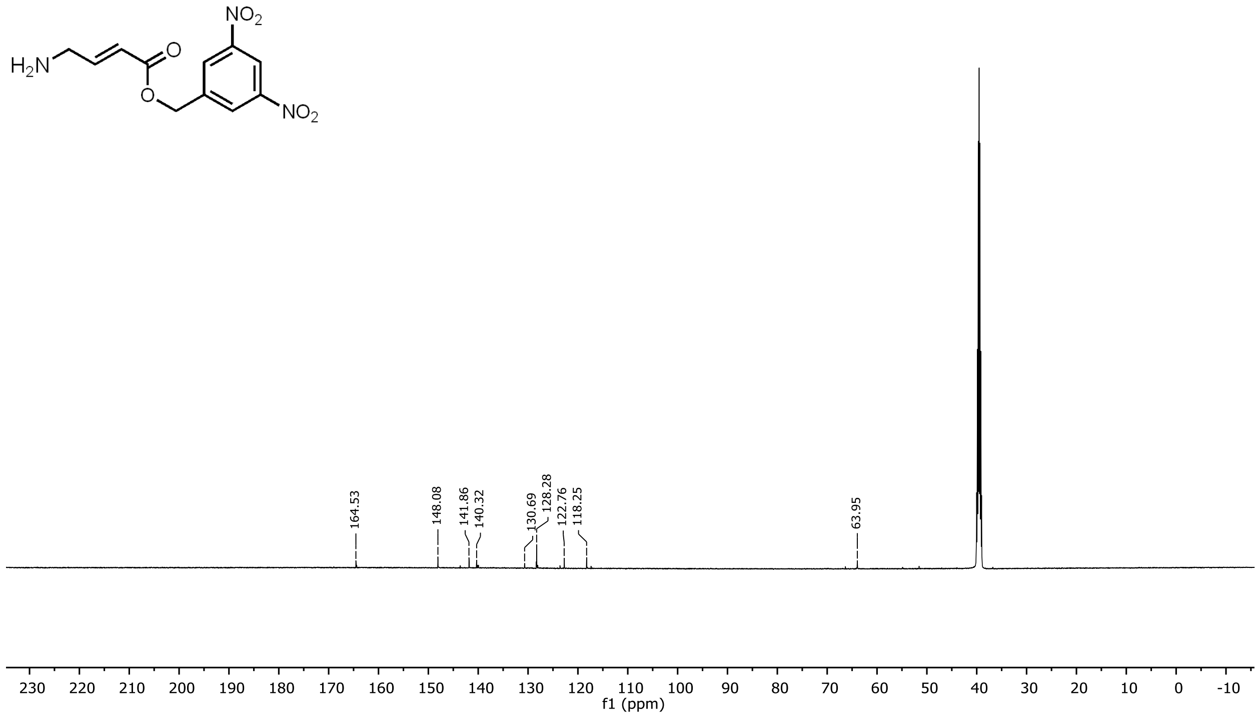

Supplementary Figure 26. ^{13}C NMR (125 MHz, DMSO-*d*$_6$) of 7.

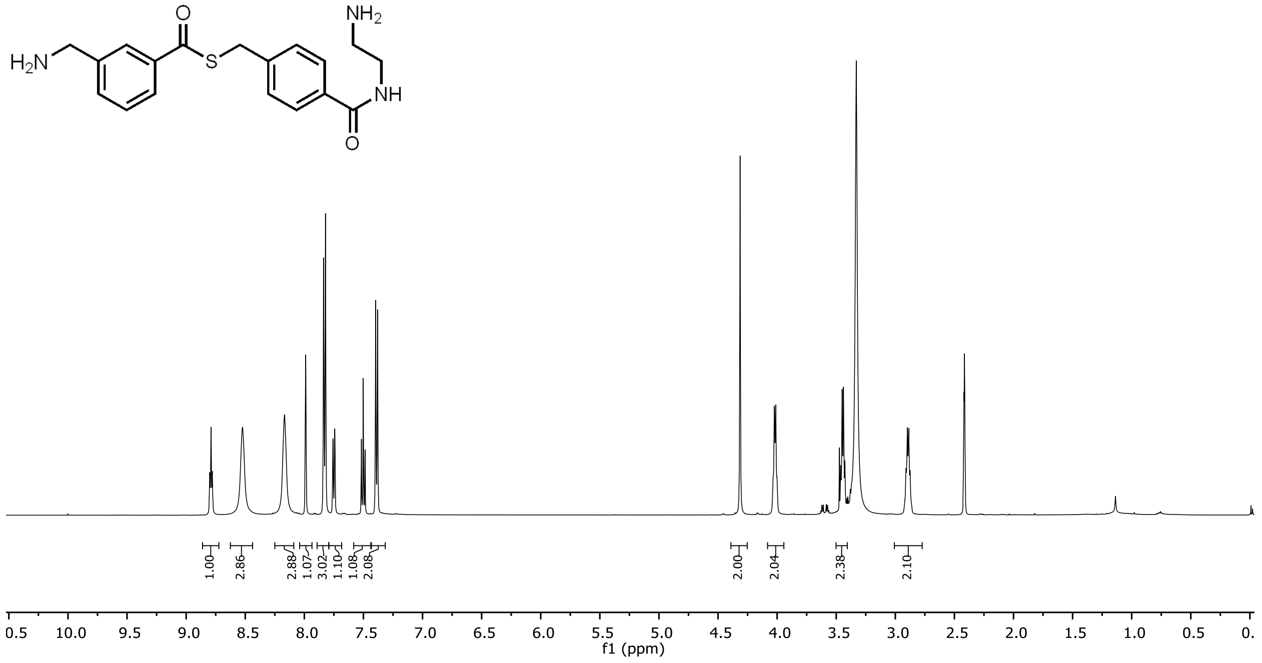

Supplementary Figure 27. ^{1}H NMR (500 MHz, DMSO-d_6) of 8.

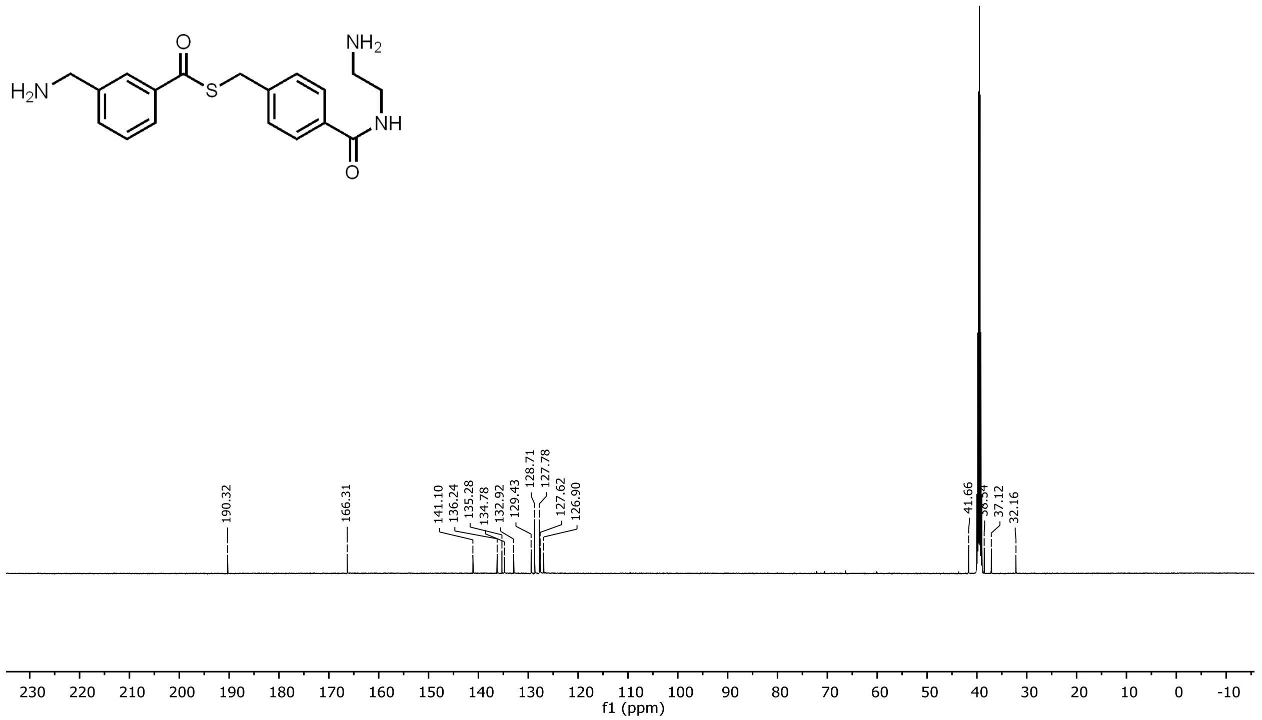

Supplementary Figure 28. ^{13}C NMR (125 MHz, DMSO-d_6) of 8.

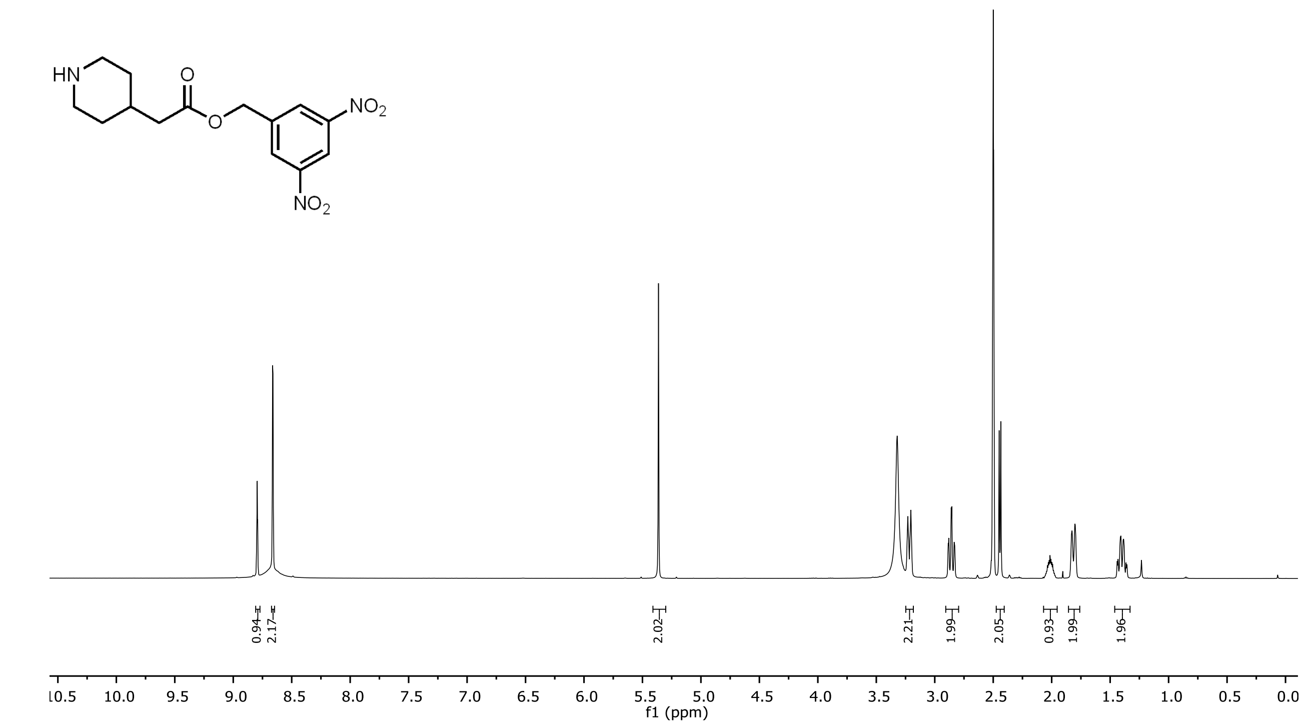

Supplementary Figure 29. ^{1}H NMR (500 MHz, DMSO-*d$_6$*) of 9.

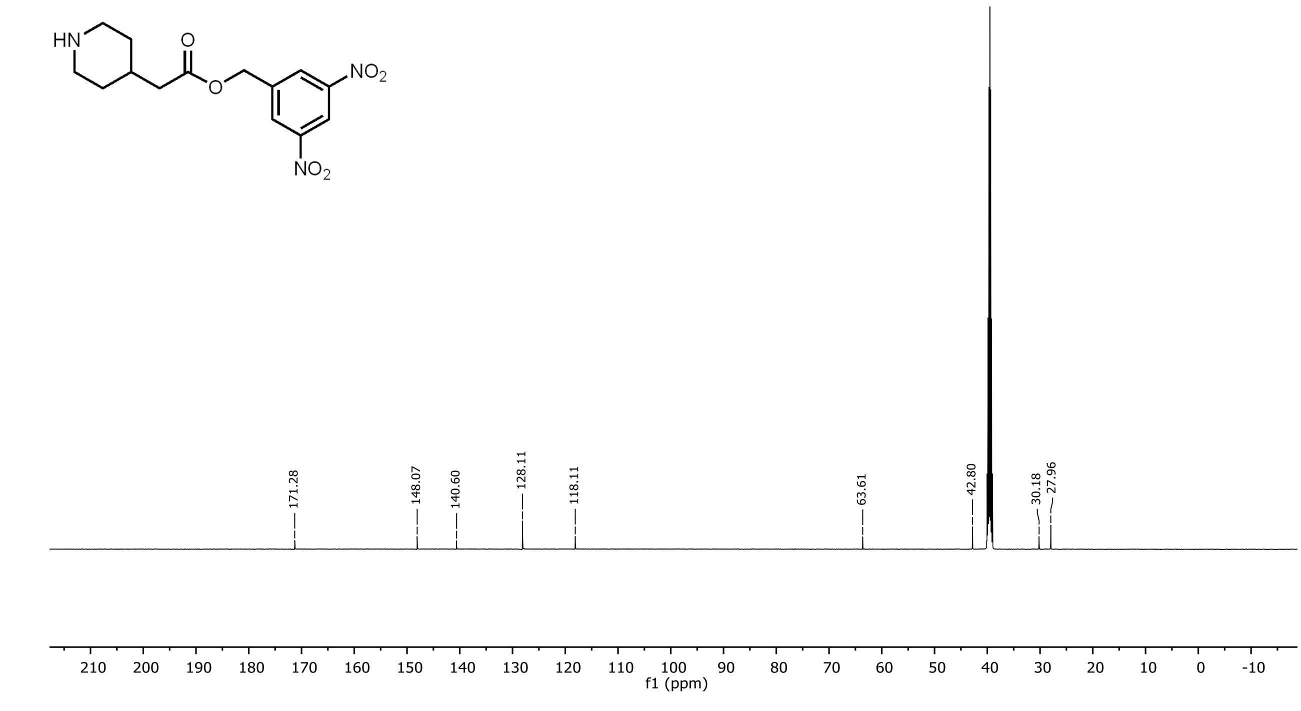

Supplementary Figure 30. ^{13}C NMR (125 MHz, DMSO-*d$_6$*) of 9.

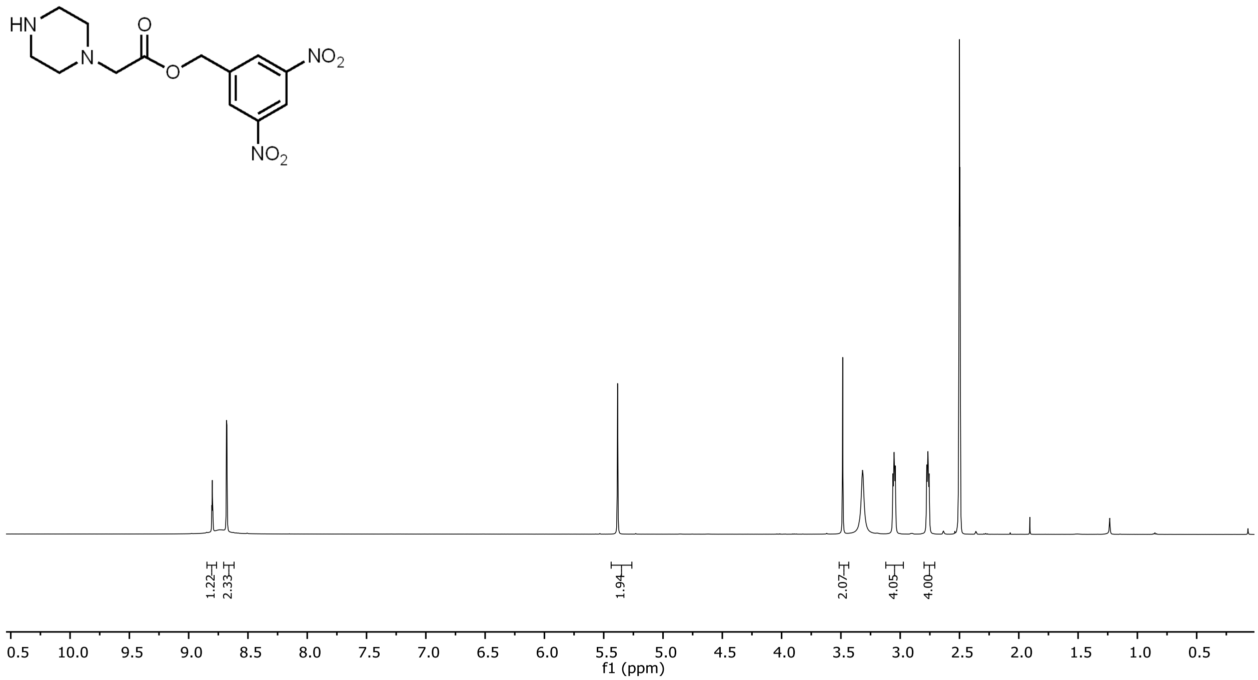

Supplementary Figure 31. ^{1}H NMR (500 MHz, DMSO-*d*$_6$) of 10.

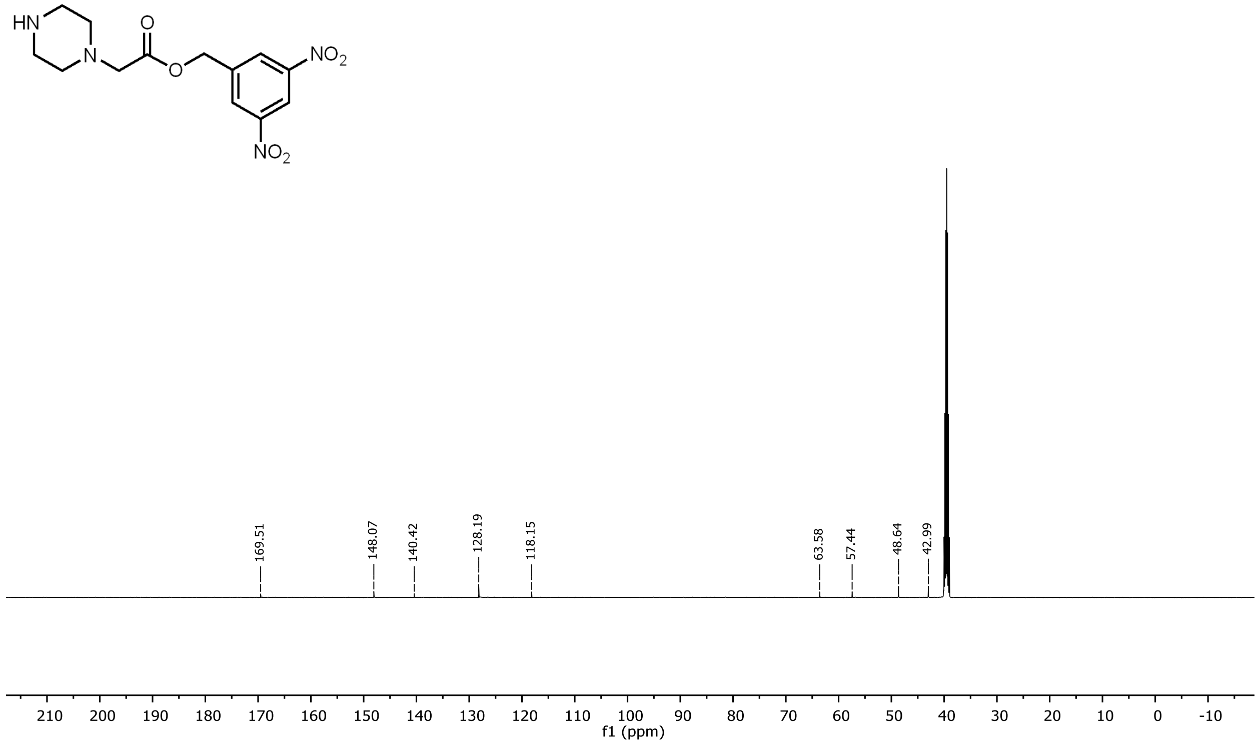

Supplementary Figure 32. ^{13}C NMR (125 MHz, DMSO-*d*$_6$) of 10.

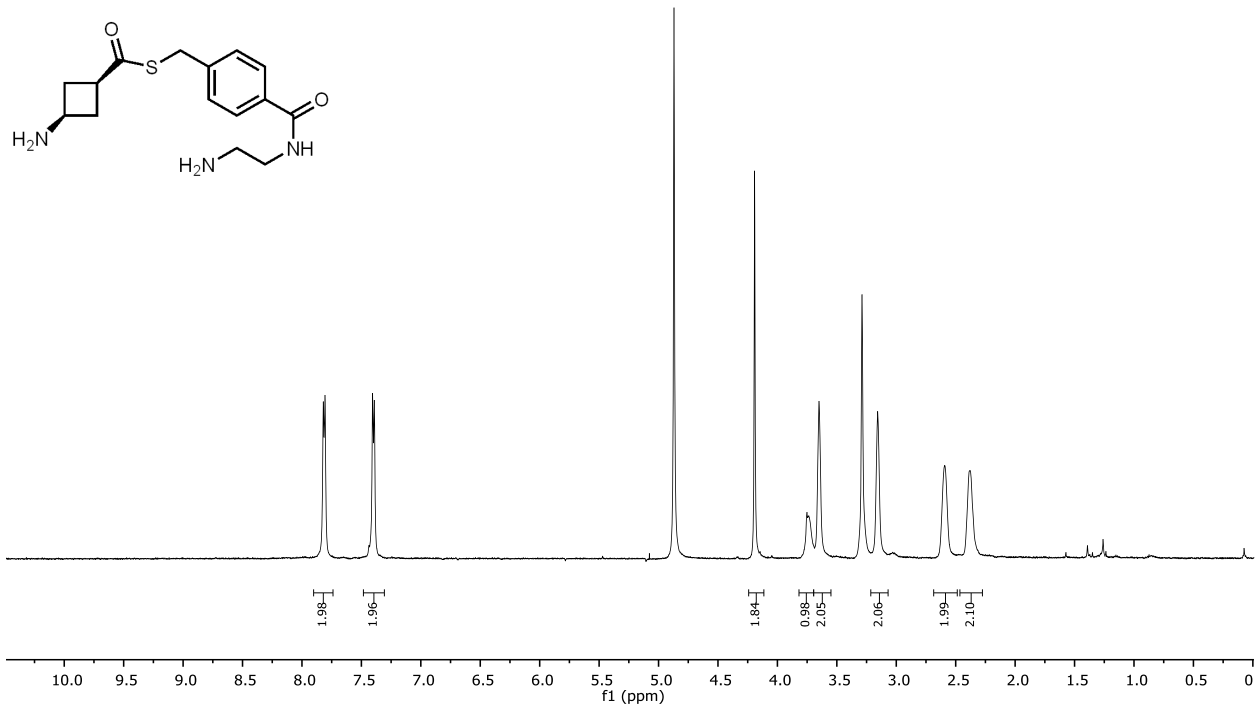

Supplementary Figure 33. ^{1}H NMR (500 MHz, DMSO-d_6) of 11.

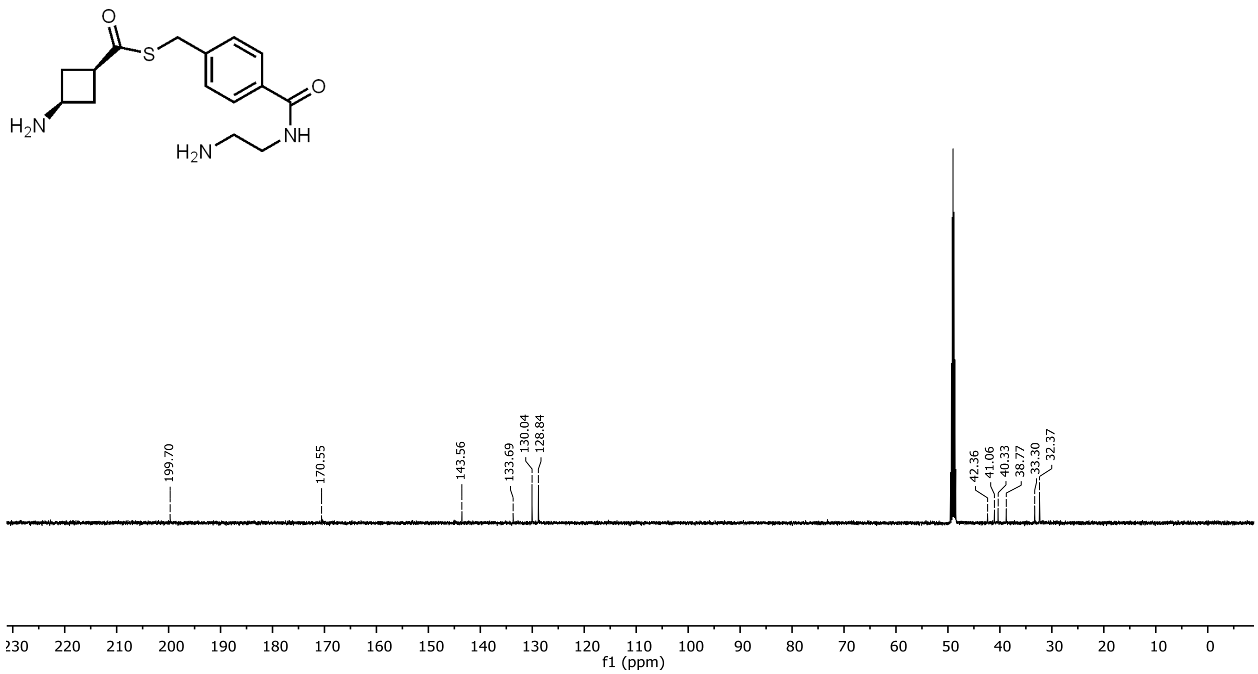

Supplementary Figure 34. ^{13}C NMR (125 MHz, DMSO-d_6) of 11.

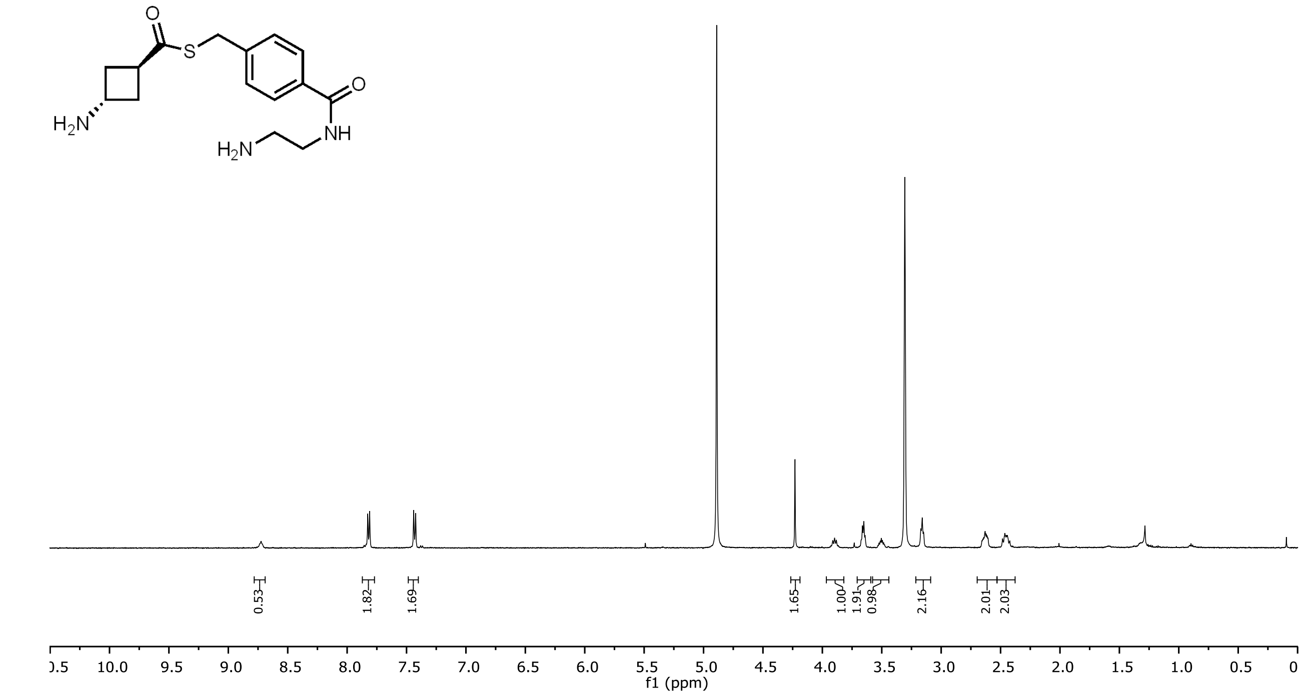

Supplementary Figure 35. ^{1}H NMR (500 MHz, DMSO-d_6) of 12.

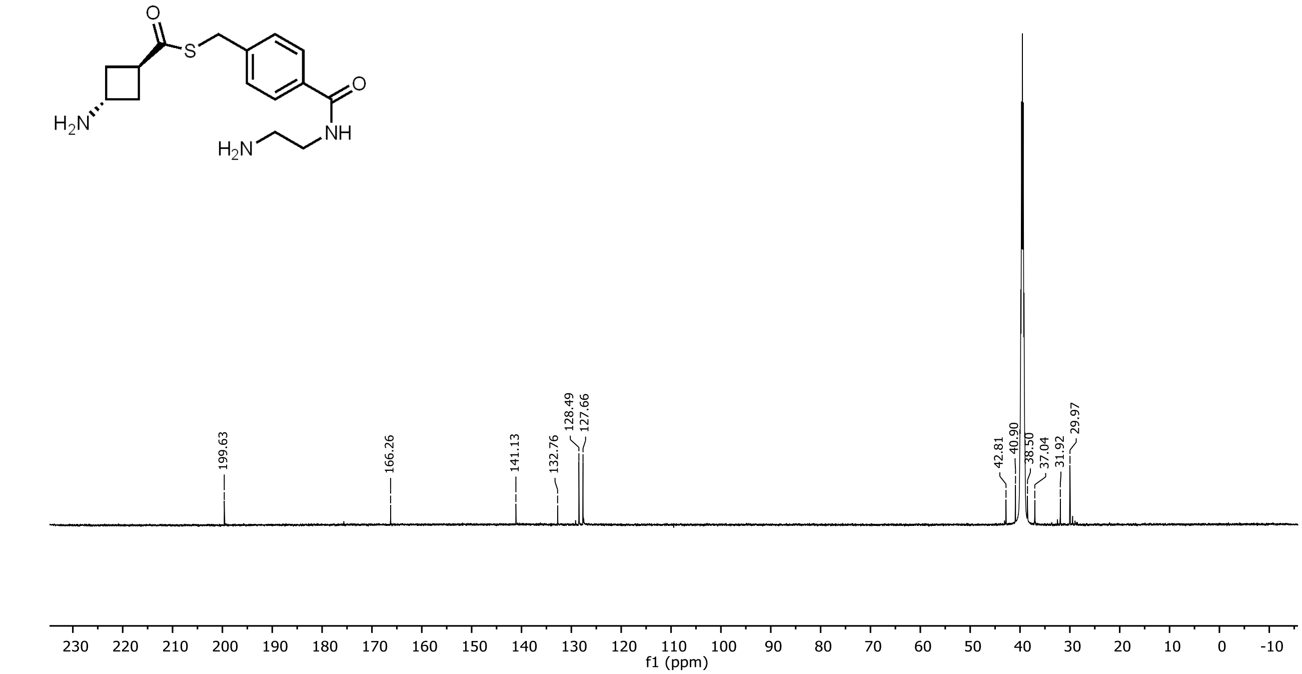

Supplementary Figure 36. ^{13}C NMR (125 MHz, DMSO-d_6) of 12.

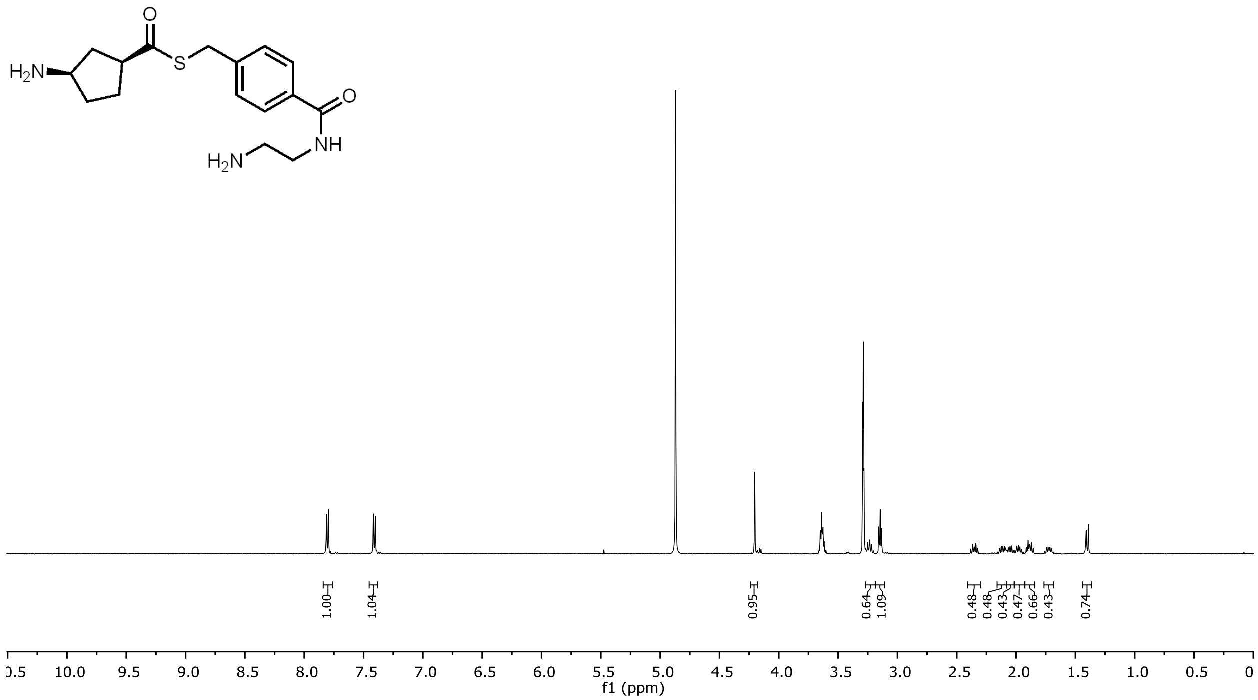

Supplementary Figure 37. ^{1}H NMR (500 MHz, DMSO-*d6*) of 13.

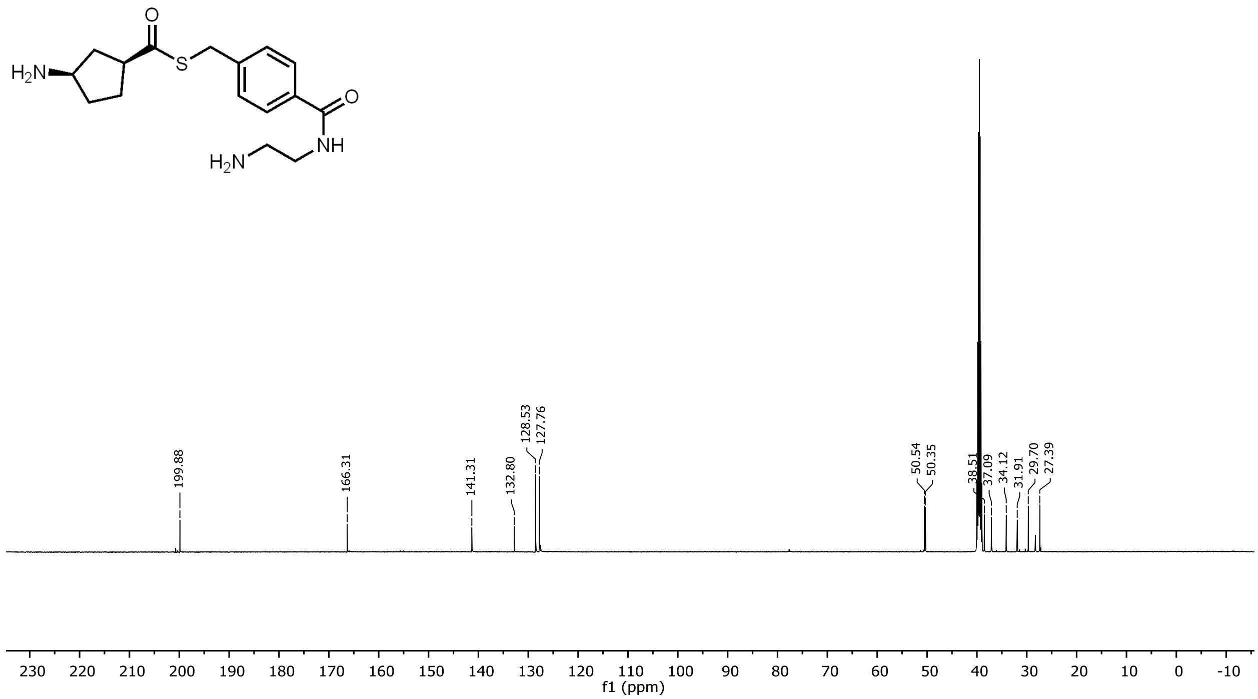

Supplementary Figure 38. ^{13}C NMR (125 MHz, DMSO-*d6*) of 13.

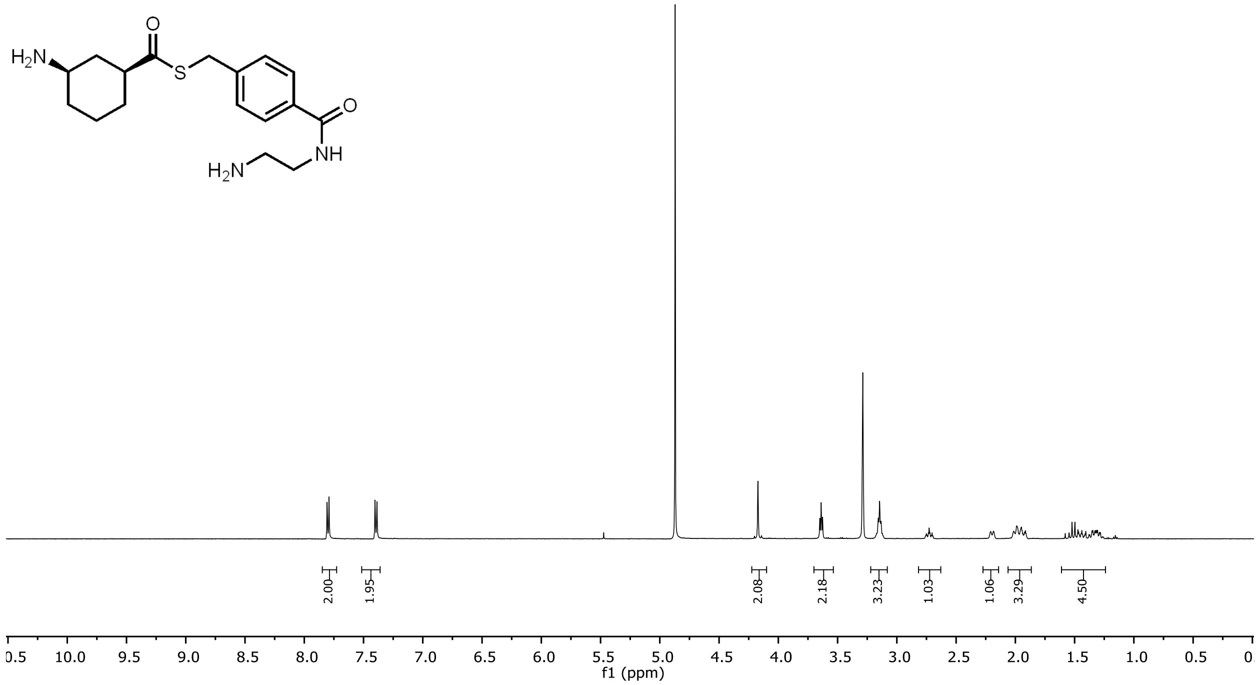

Supplementary Figure 39. ^{1}H NMR (500 MHz, DMSO-*d*$_6$) of 14.

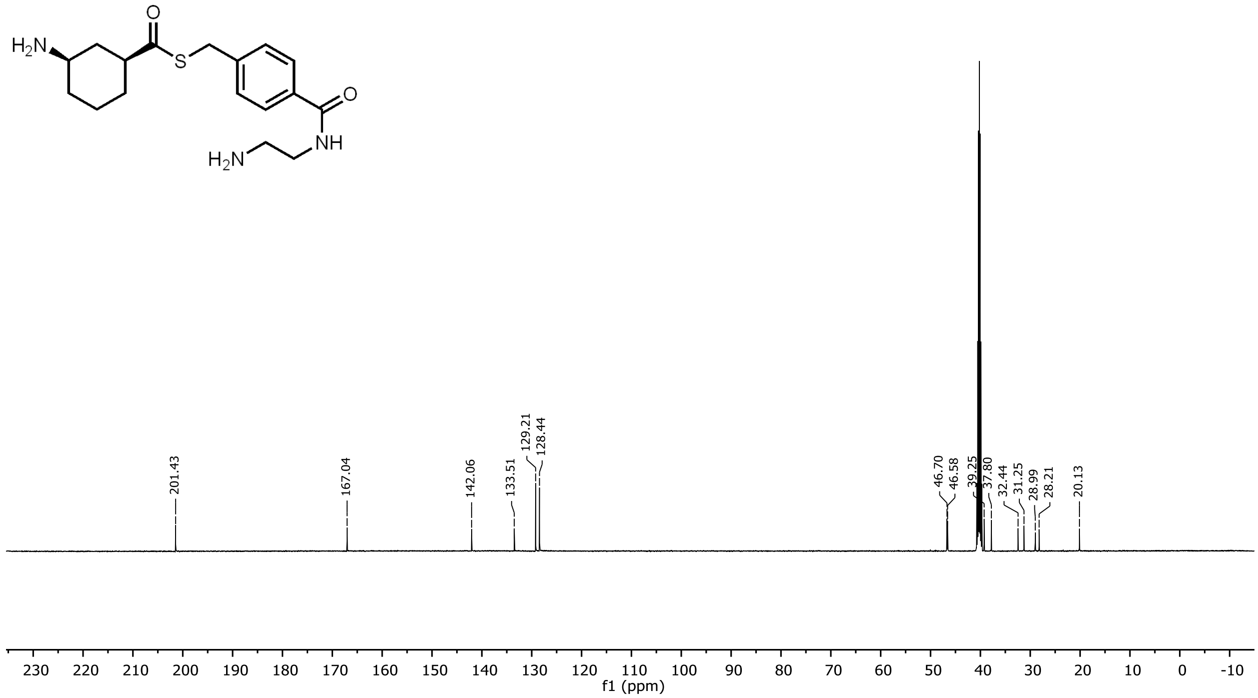

Supplementary Figure 40. ^{13}C NMR (125 MHz, DMSO-*d*$_6$) of 14.

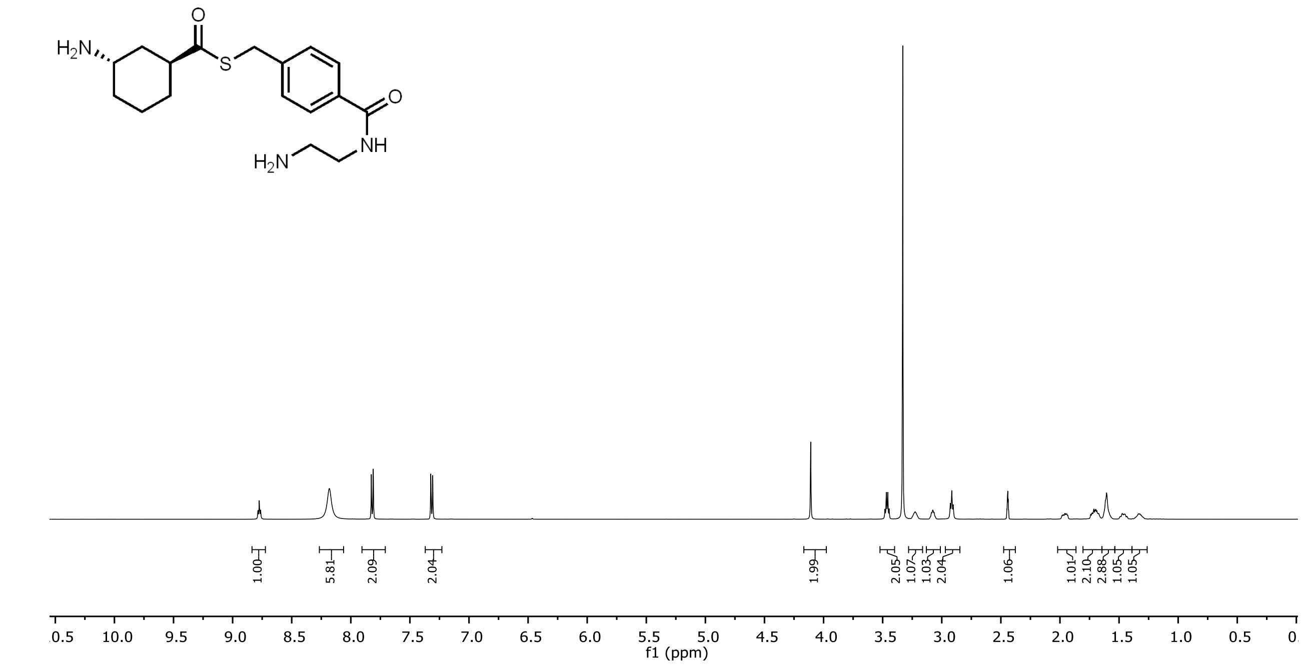

Supplementary Figure 41. ^{1}H NMR (500 MHz, DMSO-d_6) of 15.

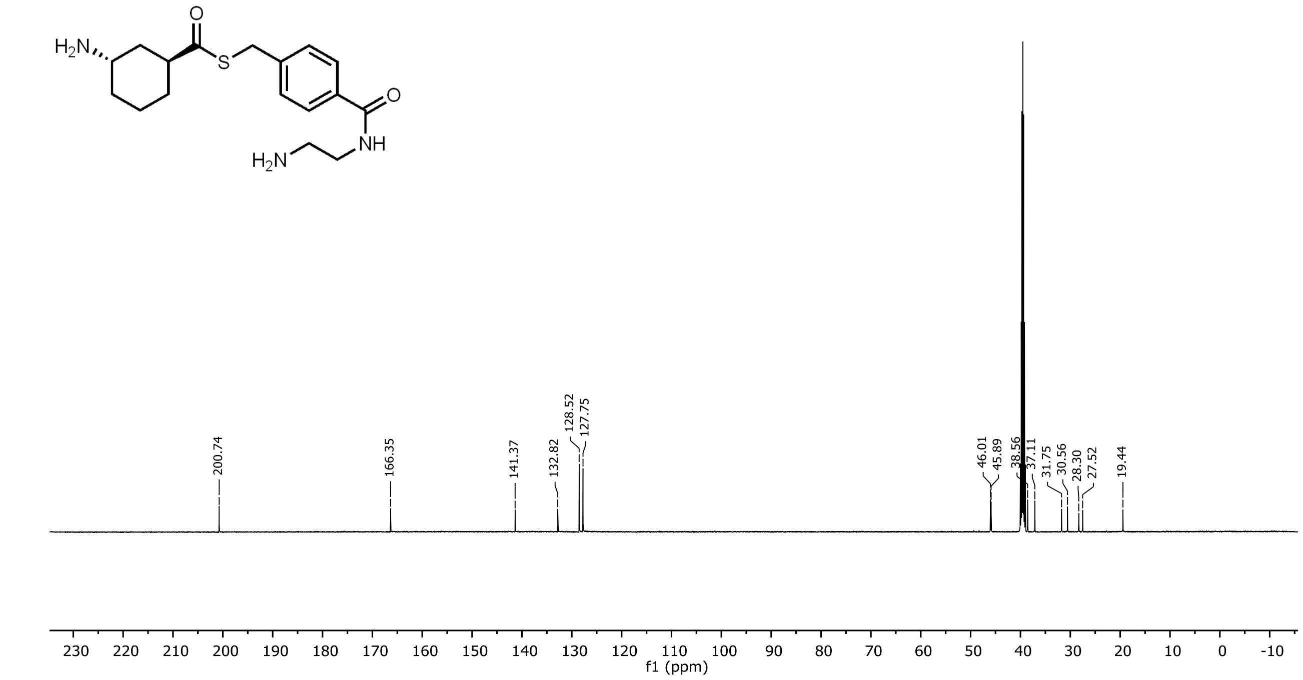

Supplementary Figure 42. ^{13}C NMR (125 MHz, DMSO-d_6) of 15.

Plasmid map

GGATCCTGCAGTTGAGATCCTTTTTTTCTGCGCGTAATCTGCTGCTTGCAAACAAAAAAACCACCGCTACCAGCGGTGGTTTGTTTGCCGGAT
CAAGAGCTACCAACTCTTTTTCCGAAGGTAACTGGCTTCAGCAGAGCGCAGATACCAAATACTGTCCTTCTAGTGTAGCCGTAGTTAGGCCAC
CACTTCAAGAACTCTGTAGCACCGCCTACATACCTCGCTCTGCTAATCCTGTTACCAGTGGCTGCTGCCAGTGGCGATAAGTCGTGTCTTACC
GGGTTGGACTCAAGACGATAGTTACCGGATAAGGCGCAGCGGTCGGGCTGAACGGGGGGTTCGTGCACACAGCCCAGCTTGGAGCGAACGACC
TACACCGAACTGAGATACCTACAGCGTGAGCATTGAGAAAGCGCCACGCTTCCCGAAGGGAGAAAGGCGGACAGGTATCCGGTAAGCGGCAGG
GTCGGAACAGGAGAGCGCACGAGGGAGCTTCCAGGGGGAAACGCCTGGTATCTTTATAGTCCTGTCGGGTTTCGCCACCTCTGACTTGAGCGT
CGATTTTTGTGATGCTCGTCAGGGGGGCGGAGCCTATGGAAACGAATTCAGATCTCGATCCCGCGAAATTAATACGACTCACTATAGGGAGAC
CACAACGGTTTCCCTCTAGAAATAATTTTGTTTAACTTTAAGAAGGAGATATA[***CATATG*****TGGTCTCATCCGCAGTTCGAAAAATCCACCTAG**
TAA***GTCGAC***]CGGCTGCTAACAAAGCCCGAAAGGAAGCTGAGTTGGCTGCTGCCACCGCTGAGCAATAACTAGCATAACCCCTTGGGGCCTCT
AAACGGGTCTTGAGGGGTTTTTTGCTGAAAGCCAATTCTGATTAGAAAAACTCATCGAGCATCAAATGAAACTGCAATTTATTCATATCAGGA
TTATCAATACCATATTTTTGAAAAAGCCGTTTCTGTAATGAAGGAGAAAACTCACCGAGGCAGTTCCATAGGATGGCAAGATCCTGGTATCGG
TCTGCGATTCCGACTCGTCCAACATCAATACAACCTATTAATTTCCCCTCGTCAAAAATAAGGTTATCAAGTGAGAAATCACCATGAGTGACG
ACTGAATCCGGTGAGAATGGCAAAAGCTTATGCATTTCTTTCCAGACTTGTTCAACAGGCCAGCCATTACGCTCGTCATCAAAATCACTCGCA
TCAACCAAACCGTTATTCATTCGTGATTGCGCCTGAGCGAGACGAAATACGCGATCGCTGTTAAAAGGACAATTACAAACAGGAATCGAATGC
AACCGGCGCAGGAACACTGCCAGCGCATCAACAATATTTTCACCTGAATCAGGATATTCTTCTAATACCTGGAATGCTGTTTTCCCGGGGATC
GCAGTGGTGAGTAACCATGCATCATCAGGAGTACGGATAAAATGCTTGATGGTCGGAAGAGGCATAAATTCCGTCAGCCAGTTTAGTCTGACC
ATCTCATCTGTAACATCATTGGCAACGCTACCTTTGCCATGTTTCAGAAACAACTCTGGCGCATCGGGCTTCCCATACAATCGATAGATTGTC
GCACCTGATTGCCCGACATTATCGCGAGCCCATTTATACCCATATAAATCAGCATCCATGTTGGAATTTAATCGCGGCTTCGAGCAAGACGTT
TCCCGTTGAATATGGCTCATAACACCCCTTGTATTACTGTTTATGTAAGCAGACAGTTTTATTGTTCATGATGATATATTTTTATCTTGTGCA
ATGTAACATCAGAGATTTTGAGACACAACGT

***CATATG*: NdeI**
***GTCGAC*: SalI**
TGGTCTCATCCGCAGTTCGAAAAA: strep tag
TAGTAA: stop

>pJL1_StrepII
[*CATATG*TGGTCTCATCCGCAGTTCGAAAAATCCACCTAGTAA*GTCGAC*]
fMetTrpSerHisProGlnPheGluLysSerThr

>pJL1_StrepII_TIA
[*CATATG*TGGTCTCATCCGCAGTTCGAAAAATCCACCATCGCCTAGTAA*GTCGAC*]
fMetTrpSerHisProGlnPheGluLysSerThrIleAla

Supplementary References

1. Pangborn, A.B., Giardello, M.A., Grubbs, R.H., Rosen, R.K. & Timmers, F.J. Safe and convenient procedure for solvent purification. *Organometallics* **15**, 1518-1520 (1996).
2. Niwa, N., Yamagishi, Y., Murakami, H. & Suga, H. A flexizyme that selectively charges amino acids activated by a water-friendly leaving group. *Bioorg Med Chem Lett* **19**, 3892-3894 (2009).
3. Lee, J. et al. Expanding the limits of the second genetic code with ribozymes. *Nat Commun* **10**, 5097 (2019).
