# Supplementary material for: Ribosome-mediated polymerization of long chain carbon and cyclic amino acids into peptides in vitro
Source: Nat Commun. 2020 Aug 27;11:4304. doi: 10.1038/s41467-020-18001-x (PMC7452890; doi:10.1038/s41467-020-18001-x)
Supplement: Supplementary file 2 — Reporting Summary [file 41467_2020_18001_MOESM2_ESM.pdf]

nature research

Corresponding author(s): Michael Jewett

Last updated by author(s): Jul 22, 2020

Reporting Summary

Nature Research wishes to improve the reproducibility of the work that we publish. This form provides structure for consistency and transparency in reporting. For further information on Nature Research policies, see Authors & Referees and the Editorial Policy Checklist .

Statistics

For all statistical analyses, confirm that the following items are present in the figure legend, table legend, main text, or Methods section.

n/a	Confirmed	
☐	☒	The exact sample size (*n*) for each experimental group/condition, given as a discrete number and unit of measurement
☐	☒	A statement on whether measurements were taken from distinct samples or whether the same sample was measured repeatedly
☒	☐	The statistical test(s) used AND whether they are one- or two-sided<br>*Only common tests should be described solely by name; describe more complex techniques in the Methods section.*
☒	☐	A description of all covariates tested
☒	☐	A description of any assumptions or corrections, such as tests of normality and adjustment for multiple comparisons
☐	☒	A full description of the statistical parameters including central tendency (e.g. means) or other basic estimates (e.g. regression coefficient) AND variation (e.g. standard deviation) or associated estimates of uncertainty (e.g. confidence intervals)
☒	☐	For null hypothesis testing, the test statistic (e.g. *F*, *t*, *r*) with confidence intervals, effect sizes, degrees of freedom and *P* value noted<br>*Give P values as exact values whenever suitable.*
☒	☐	For Bayesian analysis, information on the choice of priors and Markov chain Monte Carlo settings
☒	☐	For hierarchical and complex designs, identification of the appropriate level for tests and full reporting of outcomes
☒	☐	Estimates of effect sizes (e.g. Cohen's *d*, Pearson's *r*), indicating how they were calculated

Our web collection on statistics for biologists contains articles on many of the points above.

Software and code

Policy information about availability of computer code

Data collection	All data were collected using stated instruments and associated commercially available software. The nuclear magnetic resonance spectra (NMR) were processed using TopSpin v4.0.5 (Bruker), ACD v12.01 (ACD/Labs), or Mnova (v14). ESI mass spectra were obtained using HyStar v4.1 (Bruker) or MassLynx 4.1 (Waters). MALDI mass spectra was obtained using FlexControl v2.0 (Bruker). No in-house algorithms or softwares were used to collect the data.
Data analysis	All data were analyzed using stated instruments and associated commercially-available softwares. For NMR spectroscopic analysis, the Jewett group in Northwestern University used TopSpin v4.0.5 (Bruker), and the Moore group in the University of Illinois used ACD v12.01 (ACD/Labs) or Mnova (v14). For mass spectrometric analysis, the Jewett HyStar v4.1 (Bruker) and FlexControl v2.0 (Bruker) and Moore group used and MassLynx 4.1 (Waters). The Jewett group used Image for densitometric analysis. No custom algorithms or softwares were used for analysis.

For manuscripts utilizing custom algorithms or software that are central to the research but not yet described in published literature, software must be made available to editors/reviewers. We strongly encourage code deposition in a community repository (e.g. GitHub). See the Nature Research guidelines for submitting code & software for further information.

Data

Policy information about availability of data

All manuscripts must include a data availability statement. This statement should provide the following information, where applicable:

- Accession codes, unique identifiers, or web links for publicly available datasets
- A list of figures that have associated raw data
- A description of any restrictions on data availability

All data generated or analyzed in this study are included in this published article (and its supplementary files) or are available from the corresponding authors upon reasonable request.

Field-specific reporting

Please select the one below that is the best fit for your research. If you are not sure, read the appropriate sections before making your selection.

☒ Life sciences ☐ Behavioural & social sciences ☐ Ecological, evolutionary & environmental sciences

For a reference copy of the document with all sections, see nature.com/documents/nr-reporting-summary-flat.pdf

Life sciences study design

All studies must disclose on these points even when the disclosure is negative.

Sample size	The sample size (n=20) we provide in the manuscript for noncanonical chemical substrates was determined based on the number of carbons on the amino acid backbone chain that give different kinetics on the lactam formation. The sample size we used was sufficient enough to show the classical trend that the amino acids with a longer and bulkier cyclic carbon chain are cyclized in a significantly slower rate.<br>This sample size is standard practice for deducing a trend in biochemistry or organic chemistry, and the sample size did not impact the result of our study.
Data exclusions	No data were excluded from the methods analysis, all analyses were performed as described in the Materials and Methods.
Replication	For the experiments not described in this manuscript, we also validate the reproducibility of the experiment by performing multiple experiments (n>2). The experiments are validated by performing the experiment by different researcher and/or using the same materials prepared by different person.<br>For the experiments described in the manuscript, we designed 5 replications to validate a sample-to-sample variation. For example, all the different five gamma amino acids with a linear carbon chain showed the fastest lactam formation rate and were not charged by Fx, indicating the replication were successful.
Randomization	We provided systematically expanded the scaffold of non-canonical chemical substrates in which the substrates were incorporated into the N- or C-terminus of a peptide. All peptides containing non-canonical chemical substrate were analyzed by mass spectrometry. Therefore, no randomization was used. No animals or human participants were used in this study.
Blinding	In this method development and descriptive study, no blinding of the data was applied or appropriate as no novel comparative group effects were determined.

Reporting for specific materials, systems and methods

We require information from authors about some types of materials, experimental systems and methods used in many studies. Here, indicate whether each material, system or method listed is relevant to your study. If you are not sure if a list item applies to your research, read the appropriate section before selecting a response.

Materials & experimental systems

n/a	Involved in the study	
☐	☒	Antibodies
☒	☐	Eukaryotic cell lines
☒	☐	Palaeontology
☒	☐	Animals and other organisms
☒	☐	Human research participants
☒	☐	Clinical data

Methods

n/a	Involved in the study	
☒	☐	ChIP-seq
☒	☐	Flow cytometry
☒	☐	MRI-based neuroimaging

Antibodies

Antibodies used	MagStrep type3 XT Beads 5% suspension (iba GmbH)
Validation	We used commercially available Strep-Tactin protein coated ferri-magnetic beads for purification of produced peptides containing a StrepII tag (WSHPQFEK) and a non-canonical chemical substrate. This product was validated by the vendor. No secondary antibodies were used.<br>MagStrep "type3" XT Beads (Strep-Tactin®XT coated magnetic beads, 5 % (v/v) suspension) have a high binding capacity combined with very low non-specific protein binding. Strep-Tactin XT is a mutein of streptavidin, an extracellular protein of Streptomyces avidinii that binds biotin with high affinity.Strep-tag® II fusion proteins: up to 0.85 nmol/µl beads (e.g. 25.5 µg of a 30 kDa protein).
